# Artificial Intelligence‐Driven Inverse Design of Singlet Fission Candidates in the Acene family

**DOI:** 10.1002/advs.76828

**Published:** 2026-09-27

**Authors:** Rafael G. Uceda, Boris Pérez‐Cañedo, Sandra Míguez‐Lago, Carlos M. Cruz, Joaquín J. Torres, Omar Núñez, Luis Álvarez de Cienfuegos, Antonio J. Mota, Delia Miguel, Juan M. Cuerva

**Affiliations:** ^1^ Departamento de Química Orgánica, Facultad De Ciencias Universidad De Granada (UGR) Granada Spain; ^2^ Unidad de Excelencia de Química Aplicada a la Biomedicina y Medioambiente (UEQ) Universidad De Granada Granada Spain; ^3^ Cátedra de Energía y Tecnologías Cuánticas Universidad De Granada Granada Spain; ^4^ Instituto ‘Carlos I’ De Física Teórica y Computacional. Departamento de Electromagnetismo y Física de La Materia Universidad De Granada (UGR) Granada Spain; ^5^ Instituto De Investigación Biosanitaria Avda Granada Madrid Spain; ^6^ Departamento de Química Inorgánica Facultad De Ciencias Granada Spain; ^7^ Departamento de Fisicoquímica, UEQ, UGR Facultad De Farmacia Avda. Profesor Clavera s/n Granada Spain

**Keywords:** acenes, artificial intelligence, computational chemistry, singlet fission

## Abstract

In this work, we describe the successful application of an AI‐driven inverse design protocol to identify singlet fission (SF) candidates within the acene family, exploring a chemical space of ≈10^19^ molecules. Substituent effects are encoded via Hammett σ constants, providing a chemically interpretable and generalizable descriptor for unseen functional groups. A Gated Recurrent Unit (GRU)‐based Recurrent Neural Network (RNN) model is used to accurately predict excited singlet (S_1_) and triplet (T_1_) states energies. The model is coupled with optimization algorithms, including Genetic Algorithms (GA) and Particle Swarm Optimization (PSO), to efficiently navigate chemical space, optimize S_1_ and T_1_ energies, and reveal recurring substituent patterns. This approach not only identifies well‐known tetracene and pentacene candidates but also uncovers viable benzene, naphthalene, and anthracene derivatives that satisfy SF criteria, which are typically inaccessible via intuition. The methodology, accessible at https://alba.ugr.es/acene/, establishes a versatile and interpretable platform for rational molecular design, enabling the exploration of large chemical spaces and the discovery of compounds with tailored excited‐state properties.

## Graphical abstract

In this work, we describe an AI‐driven inverse design platform predicting S_1_ and T_1_ energies across acenes with high accuracy. Using Hammett σ constants as descriptors and RNN models coupled with optimization algorithms, it efficiently explores ≈10^14^ structures, uncovering novel SF candidates from benzene to pentacene. Freely accessible at https://alba.ugr.es/acene/, it offers a versatile, interpretable tool for rational molecular design

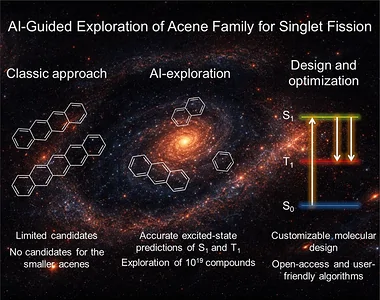

## 1 Introduction

Using chemical rules, an almost endless number of molecules can be nowadays prepared. However, the rational discovery of molecules with tailored properties within this immense chemical space remains a significant challenge and is considered one of the 33 unresolved questions in nanoscience and nanotechnology in 2025 [1]. Within this context, inverse design provides a solution, targeting the property and working backward to find a suitable structure that achieves it. Consequently, a protocol able to navigate across the chemical space searching for the optimized property is the first requirement [2, 3, 4, 5]. Two key elements are then essential: a model able to give a reliable value of the property at demand [6, 7, 8, 9], and an optimization algorithm for the searching process [10]. In this way, the chemical space can be distilled off to only provide the desired compounds. Nevertheless, it is a colossal task owing to the immense complexity of the chemical space consisting of up to 10^200^ potential structures [11, 12, 13, 14, 15]. Previously, our group has addressed this type of problem in the context of chiroptical properties, studying [6]helicenes and how substitution affects their properties [16, 17]. In this work, we show that inverse design is successful in searching for singlet fission (SF) candidates within the acene family, coupling a machine learning model with optimization algorithms in a chemical space of ≈10^19^ (Figure 1).

**FIGURE 1:**
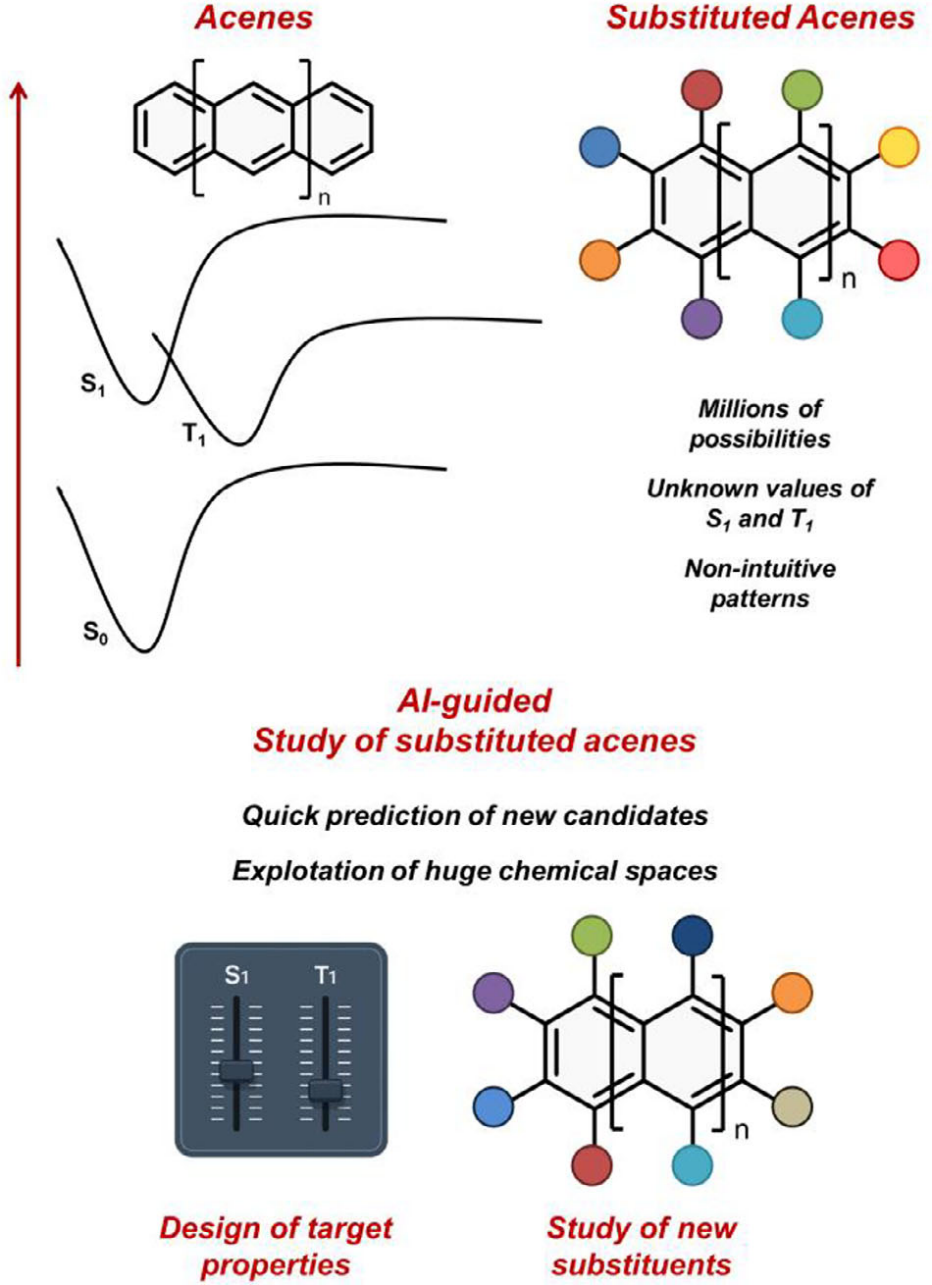
Representation of the S_0_, S_1_ and T_1_ levels and the substitution representation in the acene family used in the AI‐based methodology for the prediction of candidates.

The acene family constitutes one of the most iconic series of conjugated hydrocarbons [18, 19, 20], rooted in the discovery of benzene two hundred years ago [21, 22]. Since then, benzene and its higher homologues have served as model systems for understanding aromaticity [23, 24], π‐conjugation [25], and their impact on molecular electronic properties [26]. Moreover, some of the members present a remarkable property: singlet fission [27, 28, 29, 30, 31], with pentacene standing out as the most extensively studied example [32, 33, 34, 35]. In this process, one photoexcited singlet exciton splits into two lower‐energy triplet excitons. This phenomenon has attracted significant attention in the context of next‐generation photovoltaics, since it enables efficiencies above the Shockley–Queisser limit by effectively doubling the number of charge carriers from a single absorbed photon. Although it is a complex global process, it is rooted in a simple arithmetic: S_1_ must be (almost) isoenergetic with 2T_1_. Ideally, T_1_ values must be also higher than silicon, GaAs, or CdTe bandgaps (1.1, 1.4, and 1.5 eV, respectively) [36]. Tetracene and pentacene are the canonical examples of prototypical SF chromophores, with T_1_ values of ≈1.25 and ≈0.85 eV, respectively [37, 38], whereas anthracene has been much less explored because of the endothermicity of the SF for the naked core [39, 40, 41, 42]. For smaller acenes the situation is even worse with no examples of benzenes and naphthalenes exhibiting such a property yet.

Although previous works have used data‐driven methodologies for exploring different organic cores for SF [43, 44, 45, 46, 47, 48, 49, 50, 51], here we demonstrate that, by simply playing with substituents, it is possible to modulate the S_1_ and T_1_ levels for the whole family, thereby promoting or suppressing SF along it, even for benzene and naphthalene cores. The reason is the huge chemical space behind functionalization [12, 52]. As an example, if only six functional groups as substituents and six positions are used within the family, we are considering around 10^8^ potential structures. If no restrictions are imposed to the use of functional groups or positions, the number of possibilities scales very fast. The questions: *which acene core? which substituents? where to substitute*? and *how many of them?* make the search of suitable candidates an immense task only accessible by machine learning approaches. Furthermore, the lack of a parametrization relating the S_1_ and T_1_ state energies to the nature and positions of the substituents greatly hinders the direct and simple design process for synthetic chemists. Both challenges can be addressed effectively through inverse design.

Here, a Gated Recurrent Unit (GRU), a gating mechanism used in Recurrent Neural Network (RNN), codifies the acene structure, transforming the molecular connectivity into a suitable mathematical entry with the same size for all the cores (See the model description below and the Supporting Information). The role of the substituents is incorporated using a chemically inspired description as the classical Hammett σ constants. This cornerstone descriptor in physical organic chemistry [53, 54], quantifies in a single number the electron‐withdrawing or electron‐donating character of the substituent. That is, the use of σ constants provides a physically meaningful mapping between substituent identity and electronic response. Because multiple substituents may share identical or very similar σ values, different chemical structures may correspond to comparable electronic profiles, providing additional synthetic flexibility during candidate selection. Thus, the Hammett encoding ensures both generality and transparency, bridging classical physical organic chemistry and modern data‐driven modelling. The overall workflow of the AI‐based methodology, including molecular representation (Figure S1), GRU‐RNN prediction, and optimization strategies, is schematically summarized in Figure 2.

**FIGURE 2:**
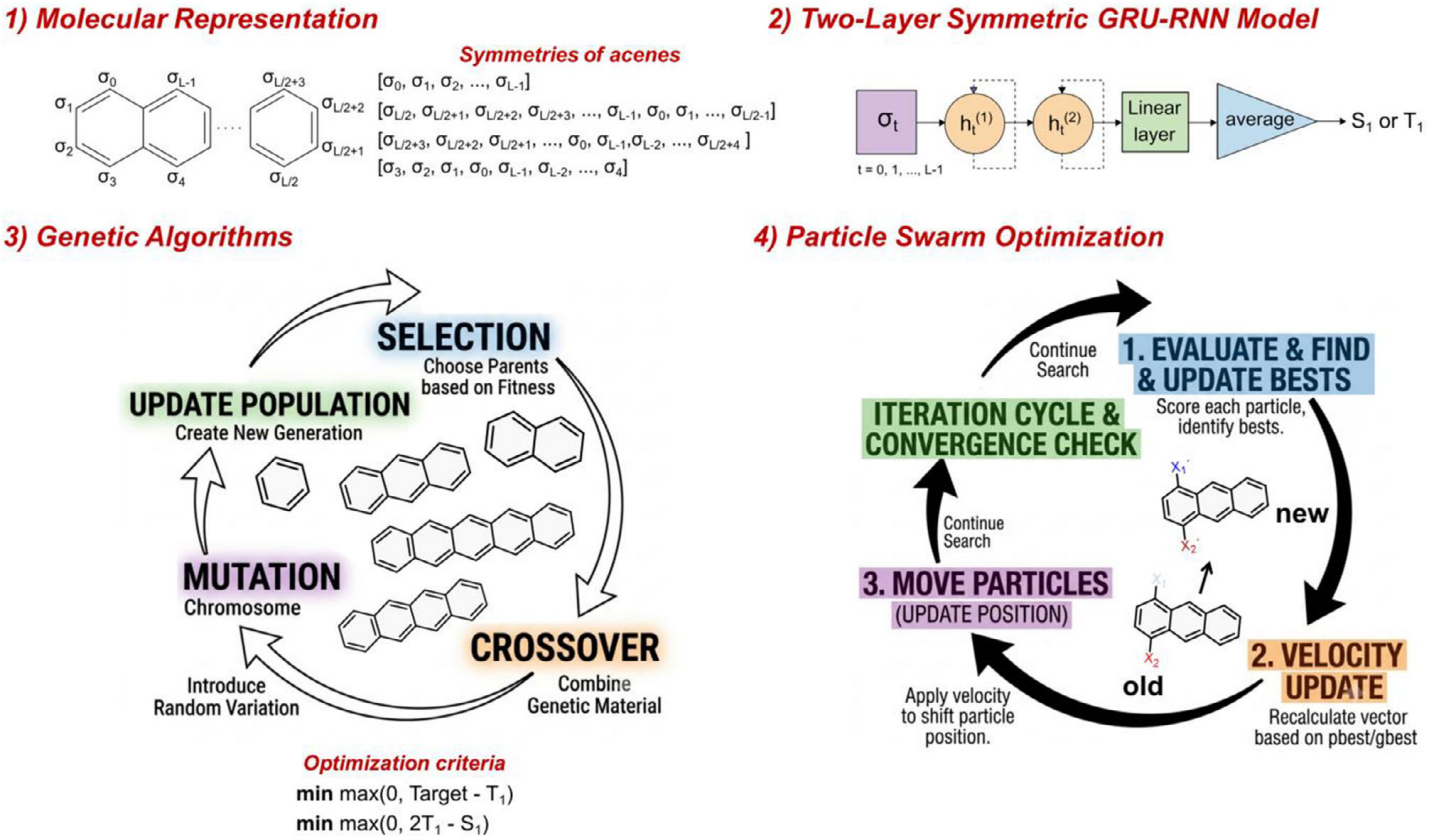
Workflow of the AI‐based methodology used for the design of acene derivatives with targeted excited‐state properties. (1) Molecular representation of acenes using Hammett σ constants to encode substituent effects and molecular symmetry. (2) Two‐layer symmetric GRU‐RNN model employed to predict the electronic properties (S_0_, S_1_, and T_1_ energies). (3) Genetic Algorithm (GA) optimization cycle and (4) Particle Swarm Optimization (PSO) strategy used to generate candidate molecules with desired properties prior to DFT validation.

GRU‐RNN is used as fitness function of Genetic Algorithms (GAs) [55, 56] or Particle Swarm Optimization (PSO) [57, 58] to generate candidates with the desired properties (Figure 2, parts 3 and 4). These proposed candidates are then validated by DFT. The versatility of using GA/PSO also allows imposing restrictions on the search related to the nature of the cores, functional groups, and their number and combinations, thus facilitating the identification of target compounds while *being compatible with synthetic protocols* [59].

With this approach, many different candidates have been found within acene family, being outstanding the case of benzene to anthracene cores. Moreover, the proposed candidates within such a huge chemical space can hardly be inferred by intuition, thus imposing a new paradigm for the rational identification of desired structures. It is worth noting that the true success of the inverse design must be evaluated by its ability to produce candidates (at DFT level) with customized S_1_ and T_1_ energies. The present inverse design approach is accessible at https://alba.ugr.es/acene/.

## 2 Results and Discussion

### 2.1 Dataset and Model Training

The electronic properties (S_1_ and T_1_) were calculated using TD‐DFT methods as implemented in Gaussian 16 (see SI) [60]. For acenes, the B3LYP functional has been shown to yield excitation energies in good agreement with experiment, with deviations typically limited to 0.1–0.2 eV [38, 61, 62, 63]. The use of TD‐DFT data assures their homogeneity and the possibility of generating more examples to cover other regions of the chemical space if needed. As for the predictive model, it must be trained with the most diverse and accurate available data. In this sense, the dataset should be constructed to include molecules that cover a broad and representative portion of the chemical space, ensuring diverse structural and property values [64]. In this case, the dataset includes S_1_ and T_1_ energies for a comprehensive set of benzene, naphthalene, anthracene, tetracene, and pentacene derivatives, totaling nearly 30,000 molecules computed at the B3LYP/6‐31G(d) level of theory. Notably, the largest subset corresponds to naphthalene derivatives (>11,000 instances), whereas comparable predictive accuracy for tetracene and pentacene is achieved with significantly fewer examples (≈4,000 and ≈6,000, respectively). The selected substituents for the training dataset (–F, –NH_2_, –OH, –CH_3_, –CN, and –NO_2_) include σ values from −0.66 to 0.78, thus covering different electronic characteristics. The unseen substituents present values within such range and can be interpolated from the previous σ values. The number of substituents along the families has been set at up to four for the training dataset, although reliable predictions are observed in up to hexa‐substituted compounds (See, Figures S2–S6). Moreover, the database can be easily expanded on demand for higher substitutions. The number was also chosen based on practical considerations, since current synthesizable molecules hardly contain more substituents, and acenes with a large number of substituents might be less appealing to experimentalists, as highly functionalized structures can be challenging to prepare.

### 2.2 Inverse design

#### 2.2.1 The Model

The predictive model uses a multi‐layer GRU‐RNN implemented through PyTorch's torch.nn.GRU module [65]. The specific GRU configuration used in this work is unidirectional with two layers and a hidden state vector of size 32. The network processes input sequences where each element contains a single feature—the Hammett constant (σ) of a substituent at the corresponding position in the acene molecule. The final hidden states from the GRU are passed to a linear transformation layer (torch.nn.Linear) [66] that maps the 32‐dimensional hidden state vector to a single output value corresponding to the target property (S_1_ or T_1_). To enforce invariance with respect to molecular symmetries, the model explicitly accounts for all automorphisms of the molecular graph. For each molecule, all symmetry‐equivalent representations are generated by applying permutations corresponding to the automorphism group of the acene structure. For benzene molecules, the full dihedral symmetry group is considered, yielding 12 equivalent sequence permutations (six rotations and six reflections). For linear acenes, four symmetry operations are used, corresponding to identity, 180° rotation, and reflections along the long and short molecular axes. Each symmetry‐equivalent sequence is processed independently by the model with shared parameters. The final prediction for a molecule is obtained by averaging the outputs over all its symmetry‐related representations. The model was trained using the Adam optimization algorithm (torch.optim.Adam) [67] with the default parameters. The complete training configuration and implementation details are provided in Section S2 of the Supporting Information. Additional models are discussed in Figures S7, and S8 of the Supporting Information. The representation of the test values in Figure 3 shows that the model is able to give reasonable predictions along the tetra‐substituted family for both optical properties and typical errors are 1%–3% for S_1_ and 1%–6% for T_1_ values, respectively. For penta‐ and hexa‐substituted derivatives, the typical errors are also acceptable (see Supporting Information).

**FIGURE 3:**
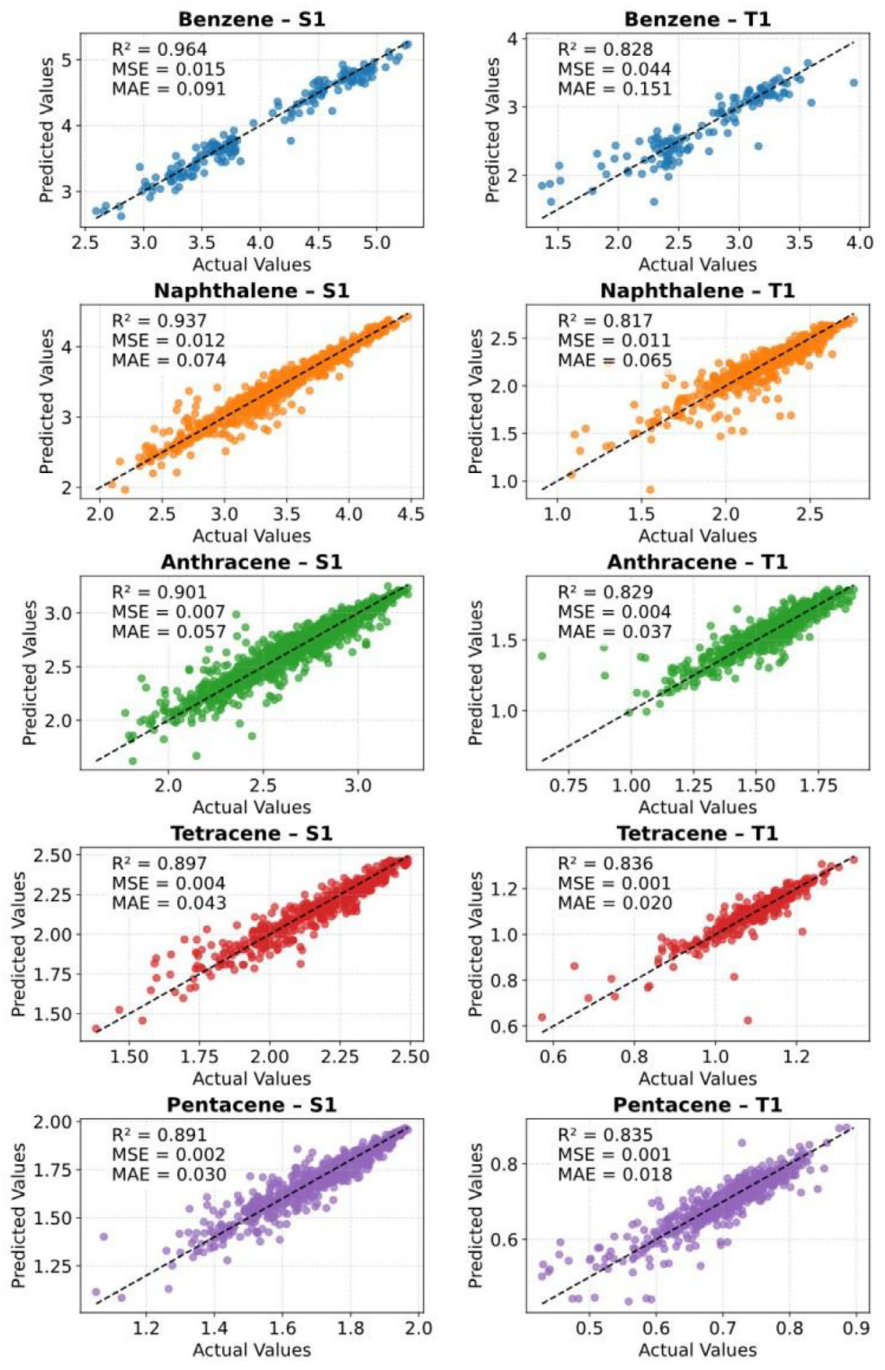
Regression of the target properties S_1_ and T_1_ for the acene family using the multi‐layer GRU‐RNN model. The Mean Absolute Error (MAE) and Mean Squared Error (MSE) metrics are computed over the entire set of points.

### 2.3 Optimization Algorithms

It is worth noting that precise quantitative predictions by the model are not essential for inverse design. From a practical perspective, we should assess the success of the approach by its ability of providing suitable candidates. Using brute force to compute all the possibilities is, in general, an inefficient procedure considering the total number of possible structures. Optimization algorithms, on the other hand, provide an alternative approach for more efficient exploration of the chemical space and enable the identification of molecules that satisfy the basic arithmetic requirements for SF—the lowest singlet excited state energy, S_1_, must be greater than or comparable to twice the triplet energy, 2T_1_. We note that this energetic criterion constitutes a necessary but not sufficient condition for efficient SF. Additional factors such as T_2_‐state energies, crystal packing, and intermolecular coupling may also play a decisive role. Therefore, the present methodology is intended as a first‐pass high‐throughput screening framework aimed at identifying promising regions of chemical space that satisfy the fundamental energetic requirements, after which more sophisticated calculations and screening steps can be applied. In this case, two optimization approaches have been used: Genetic Algorithms and Particle Swarm Optimization.

#### 2.3.1 Exploring the Discrete Space of Hammett Constants: Genetic Algorithm (GA) Approach

Results are classified as a function of acene length, distinguishing tetracenes and pentacenes, with many literature precedents of singlet fission, from benzene to anthracene, for which almost no SF‐related applications have been described.

***Tetracene and Pentacene***. In accordance with previous knowledge [68, 69, 70, 71], many substituted pentacenes and tetracenes were found. An interesting point is that the absorption edge (S_1_) can be tuned at will, and is not limited by values of prototypical unsubstituted tetracene and pentacene. That is, the absorption range can also be engineered, allowing a fine‐tuned energy window for photovoltaics. A (non‐exhaustive) list of suitable candidates ordered by S_1_ values is provided in Figure 4 and Tables S4 and S5, including both simple structures and more complex ones, all of which lie beyond intuitive chemical expectations. It is worth mentioning that SF is a very complex process and solid‐state properties are also essential for the resulting outcome. Having different alternatives even at the same S_1_ values can be useful for subsequent design of efficient exciton multiplicity and its temporal evolution [72, 73, 74], solid packaging and/or exciton mobility [75, 76].

**FIGURE 4:**
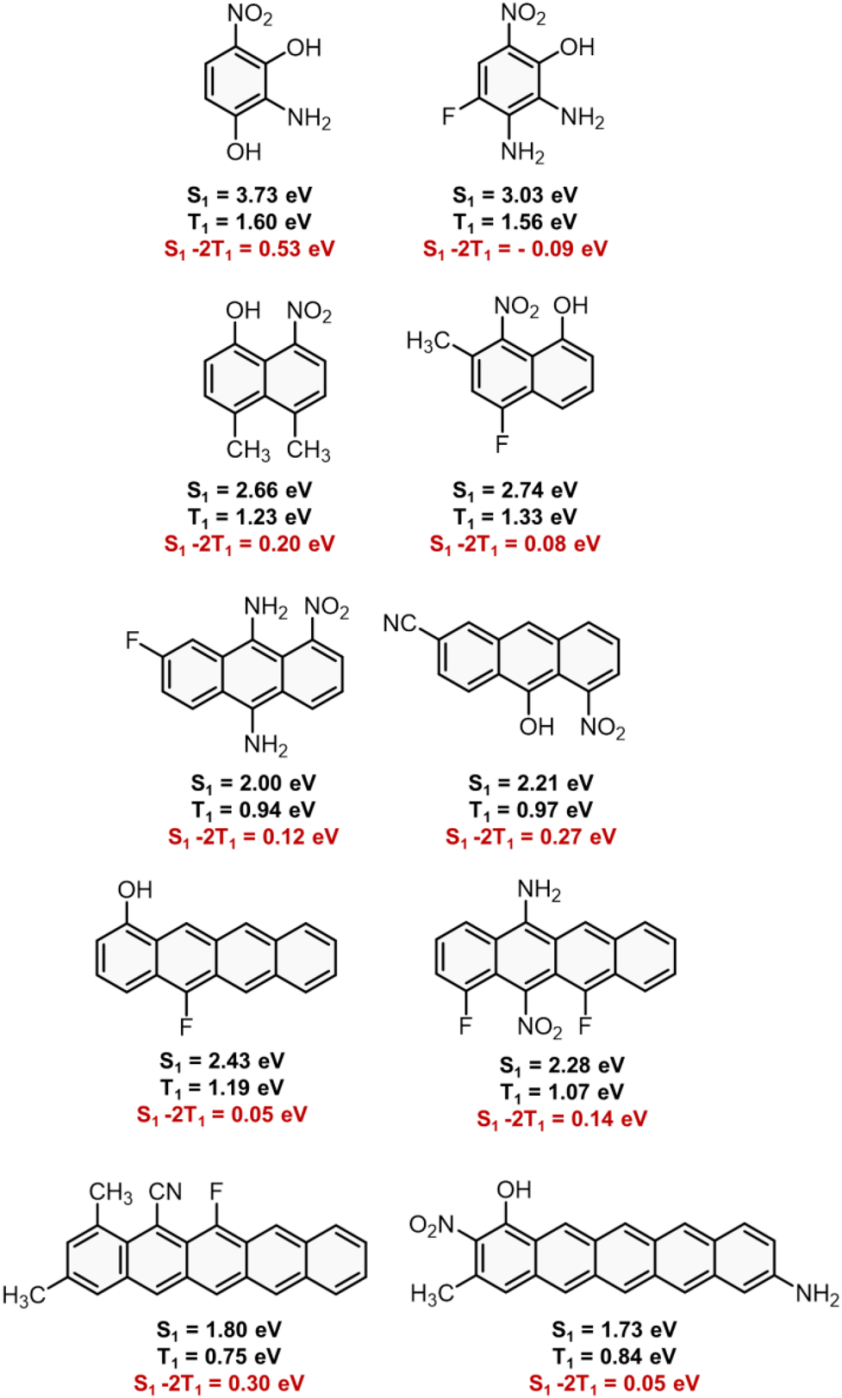
Examples of the proposed structures by the model for the different acene cores, and their excited‐state energies calculated using DFT.

***Benzene, Naphthalene and Anthracene***. More important is the fact that the GAs are able to provide suitable candidates for SF in the benzene, naphthalene and anthracene cores (Figure 4). To our knowledge, there are practically no examples with these cores due to the high T_1_ level [42, 77]. Consequently, this fact is noteworthy, showing the power of the AI‐based inverse design. A selected list of benzene, naphthalene and anthracene based‐candidates is provided in the (Tables S1–S3). Moreover, T_1_ energies above 1.4 eV have been proposed as suitable for efficient charge injection into silicon semiconductors [40], a condition that is satisfied by several of the candidates considered here.

Finally, the possible existence of multiple conformers should not be overlooked. To assess the impact of conformational flexibility on the predicted properties, conformational searches were performed using CREST software [78] for the candidates proposed by the AI‐based methodology. For all compounds shown in Figure 4, the energy levels obtained for the most populated conformer were essentially identical to those derived from the full conformational ensemble (see Table S6). This behavior reflects the dominance of a single conformer in the Boltzmann distribution, which overwhelmingly governs the overall properties. These results indicate that the fulfilment of the SF criteria is robust with respect to conformational effects, and that the predicted properties are representative of the conformational mixture as a whole. In any case, it should be emphasized that the present methodology is primarily intended as a filtering and inverse‐design strategy to identify promising regions of chemical space. Once attractive candidates are selected, more detailed analyses, including explicit conformational sampling, become essential. Indeed, while in the examples discussed here the dominant conformer is also representative of the ensemble, in other cases the conformer responsible for SF behaviour may constitute only a minor fraction of the conformational population.

***Dealing with Unseen Substituents***. As noted above, chemical information about substituents is encoded in the model through their σ constants. During training, the model was exposed to a subset of six substituents characterized by specific σ values. When asking for previously unseen substituents (unseen σ values), the model does not extrapolate new chemical identities but rather interpolates their behavior within the continuous σ‐descriptor space learned during training. In this way, the model captures the electronic nature of the substituents, treating those with similar σ values as chemically related. Therefore, GAs can be readily used to explore a much larger chemical space by incorporating new substituents into the search, allowing the use of σ constants beyond the original six. As a kind of demonstration, we allowed the use of four new prototypical functional groups: –CF_3_, –OMe, –CHO and –COOH. Thus, the algorithms localize novel candidates presenting the new and former groups. Interestingly, candidates presenting only the new groups are also localized. Some examples for all the considered cores are summarized in Figure 5 (Tables S8–S12).

**FIGURE 5:**
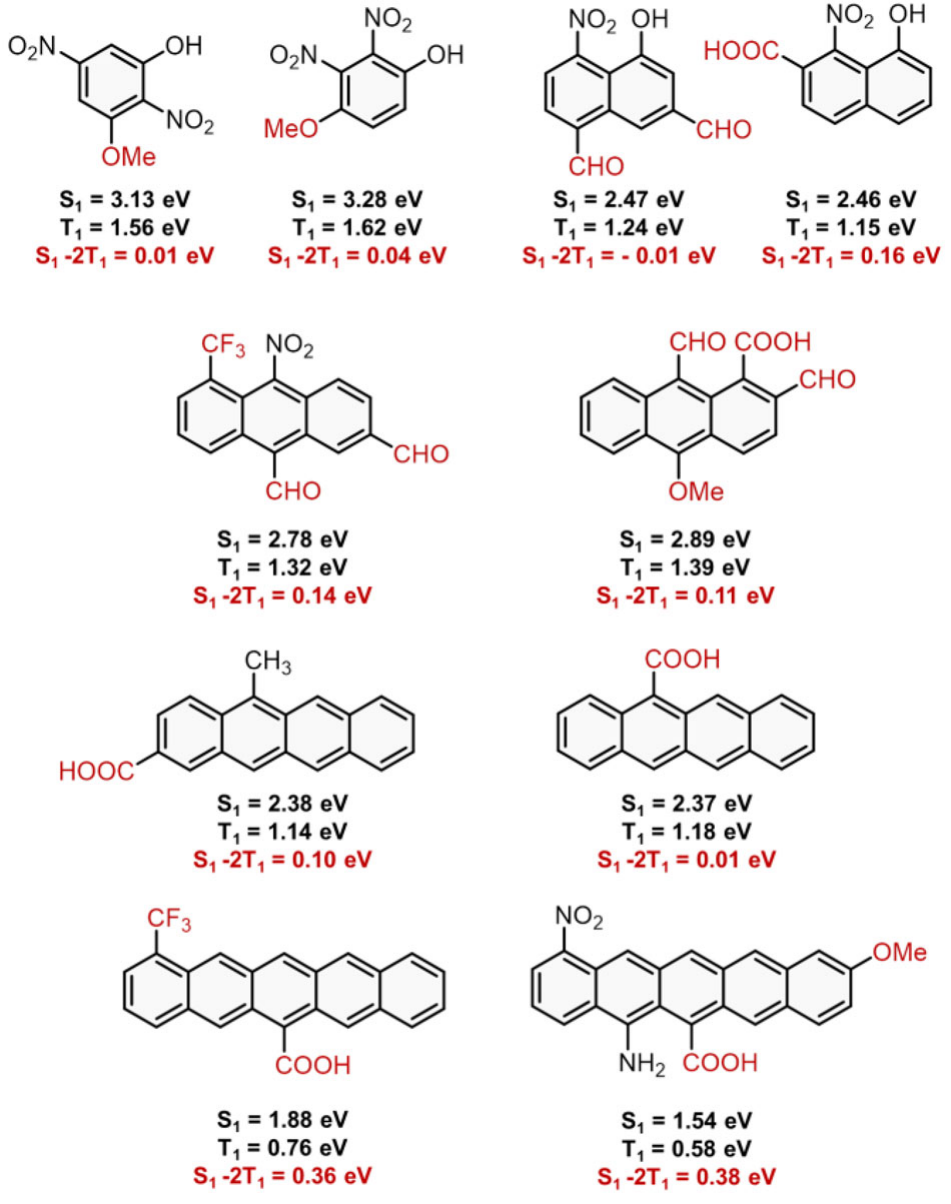
Examples of some structures proposed by the model combining functional groups included in the training dataset (black) and unseen ones (red) and their computed energies using DFT.

#### 2.3.2 Exploring the Continuum: Particle Swarm Optimization (PSO) Approach

Finally, if the σ‐constant space is regarded as a continuum rather than a set of *n* discrete values, a more versatile approach becomes possible. The optimization process can move freely in the entire chemical space to improve a property, thus proposing new σ constants. Such σ values can be then transformed in convenient functional groups. In that way, the chemical space is hugely incremented (See Figure S9 for a schematic illustration). For this purpose, herein we used the PSO implementation provided by the Distributed Evolutionary Algorithms in Python (DEAP) [79] framework (see Supporting Information), using the compounds proposed by the GAs as seeds. When fed into the PSO algorithm, up to four of the non‐hydrogen substituents are returned with alternative σ constants. Subsequently, they are transformed into corresponding unseen substituents using the full list of known σ constants (around 530) [80]. In this way, the PSO search can effectively compensate for limitations of the initial models by uncovering alternative solutions that fulfill the target requirement, extending the chemical space to ≈10^19^ molecules (six substitutions and 530 functional groups). Here we present some examples of the PSO approach up to four substitutions, in anthracene, with typical T_1_ values higher than 1.1 eV, and benzene, a more challenging test case with rarer viable candidates, cores. To explore other structures, we refer the reader to the freely accessible interactive program available at https://alba.ugr.es/acene/.

Firstly, as a proof of concept, a one‐dimensional PSO was applied to both cores, where only a single substituent was mutated (Figure 6a,b). The one‐dimensional PSO was also applied iteratively to all substituents, one at a time, generating a structurally diverse set of compounds from a single parent molecule (Figure 6c). As expected, in most cases, the substituents were replaced by groups with similar σ constants, preserving the same trend observed in the “parent” compound. It is also noteworthy that multiple substituents may share the same σ constant; consequently, a single substitution can lead to several “offspring”, all of which satisfy the desired requirements (Figure 6a). For benzene, the resulting substituent changes remain chemically reasonable (Figure 6a and Table S13), although the associated variations in σ constants are more pronounced than those observed for anthracenes. It is worth noting that PSO allows the identification of viable candidates even using as seeds compounds that initially do not satisfy the target energetic criterion (Figure 6a, right). In other words, PSO efficiently navigates the chemical space toward substituents that enforce the desired excited‐state ordering, even when the initial candidates are suboptimal.

**FIGURE 6:**
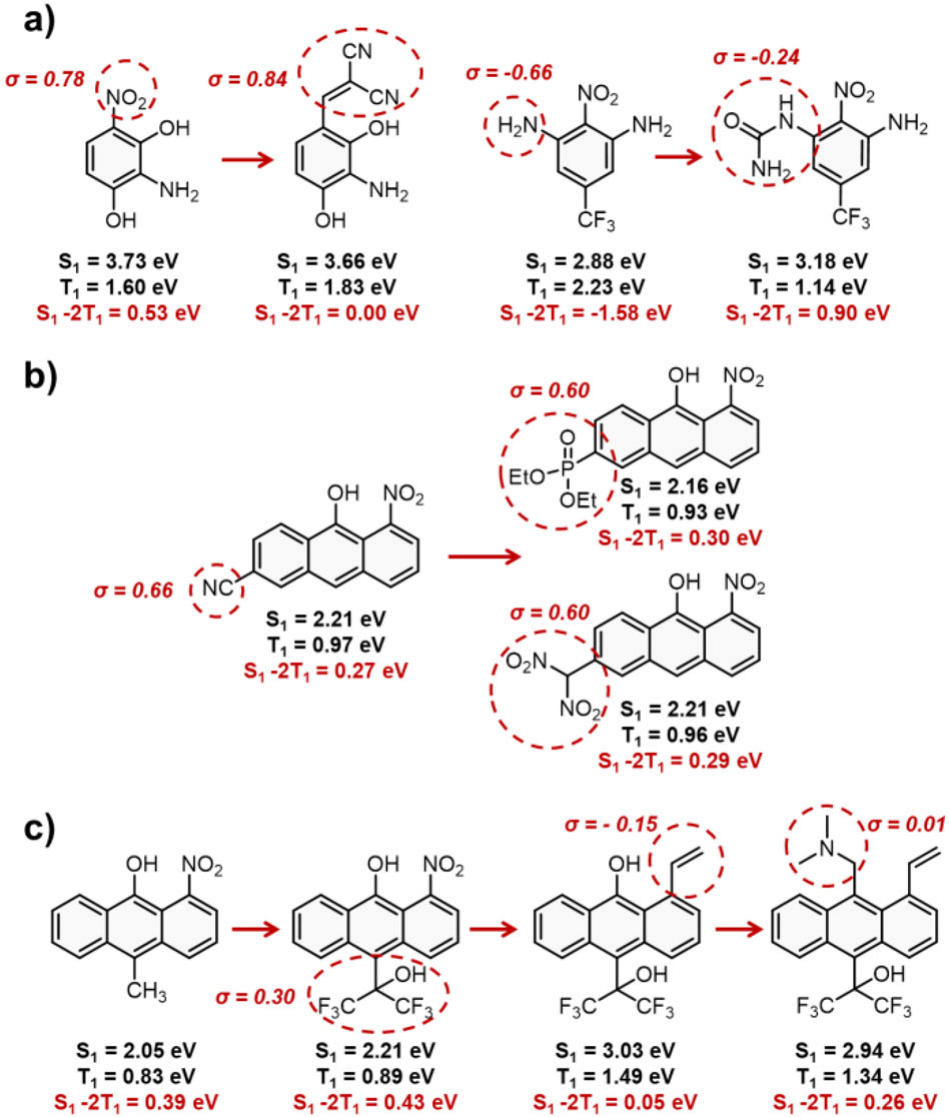
(a) Examples of the proposed benzene derivatives using the GA‐one‐dimensional‐PSO combined approach. (b) Examples of the proposed anthracenes derivatives using the GA‐one‐dimensional‐PSO combined approach. (c) Example of sequential one‐dimensional PSO for the systematic modification of all substituents.

We then performed multidimensional PSO, in which two to four substituents were modified simultaneously. Section S6 of the Supporting Information provides the detailed results (Tables S15–S17), which demonstrate how a single compound identified with a property of interest can serve as a seed to generate a vast number of new, equally valid candidates. It is worth noting that while the results of the one‐dimensional PSO were relatively intuitive, showing chemically reasonable modifications, the multidimensional PSO surpasses chemical intuition and highlights the full power of these algorithms in molecular design. When four substituents are modified simultaneously, it is quite common for all the σ Hammett constants to change substantially (Figure 7), while the target property remains essentially unchanged (Figure 7a). As in previous cases, the PSO approach corrects for these discrepancies, yielding viable candidates even when the solutions proposed by the GA fail to meet the desired criteria (Figure 7b). Here, PSO captures subtle electronic balances that are invisible to the human eye, interpreting each core as a purely N‐dimensional vector—a high‐dimensional landscape where correlations and compensations emerge naturally.

**FIGURE 7:**
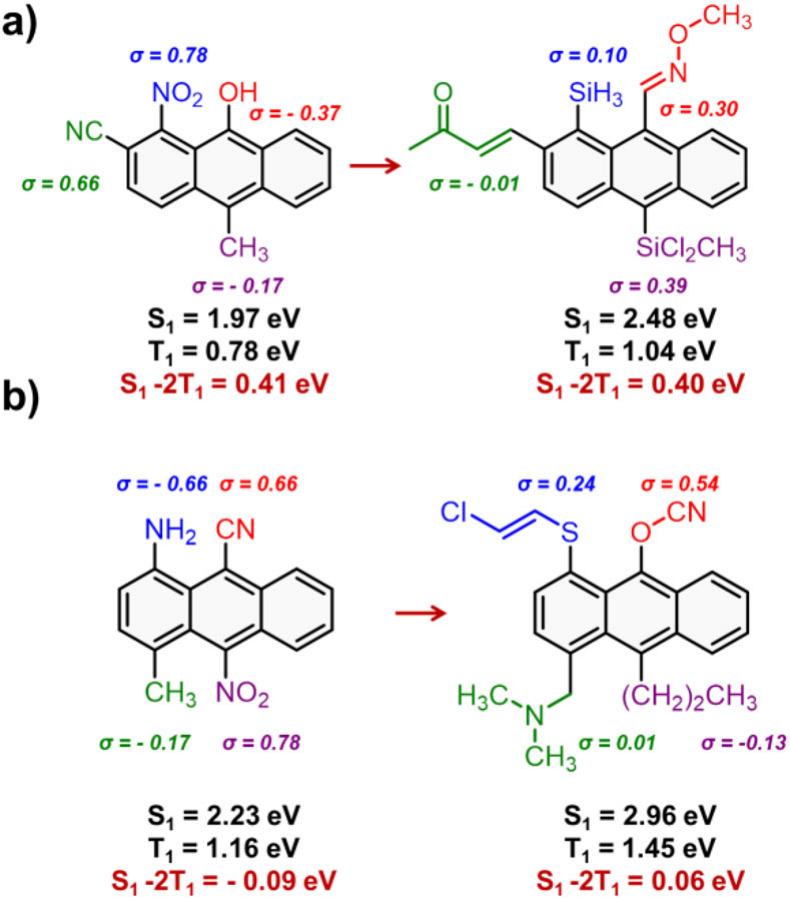
Examples of the proposed anthracene derivatives obtained using the GA–multidimensional PSO combined approach. The protocol can reproduce the target properties even when all Hammett constants are modified (a), or correct candidates proposed by the GA to yield viable solutions (b).

Examples of proposed PSO candidates with T_1_ energies lying in the 1.1–1.5 eV range can be seen in Figure 8. The methodology remains firmly grounded in experimental considerations, also allowing straightforward constraints on functional groups and molecular symmetry to be imposed with synthesizability in mind, as illustrated by the symmetric structures generated by the GA/PSO protocol shown in Figure 8b.

**FIGURE 8:**
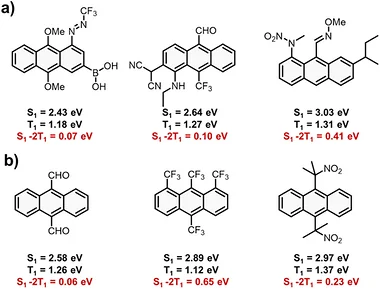
(a) Anthracene candidates for SF applications exhibiting particularly favorable energy levels. (b) Symmetric anthracene candidates for SF applications.

#### 2.3.3 Unboxing SF Predictions

As demonstrated above, these AI‐driven techniques can be extremely powerful for uncovering new privileged structures. However, at first sight their impact may seem overwhelming, sometimes giving the impression that the algorithms “take control” and unilaterally determine the optimal molecules. This can yield promising candidates but without an immediate understanding of the underlying rationale. While such concerns may be valid initially—or in certain specific cases—our results demonstrate that, in many instances, clear and chemically meaningful trends can indeed be discerned.

On the one hand, some of the suggested modifications yield substantially different σ values while still corresponding to broadly similar functional groups—for example, replacing –OH with –OCH_3_ or –O(CH_2_)_3_CH_3_, or substituting –CH_3_ with related groups such as –CH_2_CH_3_, –CH_2_CF_3_ or –C(CH_3_)_2_(CH_2_CH_3_), among others (see Tables in Section S6 of the Supporting Information). On the other hand, numerous solutions reveal recurring motifs in which donor and acceptor groups are strategically positioned to facilitate hydrogen bonding. Notably, although the model was trained on only six discrete substituents, it has learned to generalize these patterns and to propose new combinations that adjust multiple variables simultaneously in order to achieve a specific target value.

To closely examine the underlying patterns, we analyzed the anthracene family. Two significant patterns were identified and their substituent distributions are shown in Figure 9. The size of each sphere indicates the frequency with which that position is substituted, and the color encodes the average σ value of the substituent. Both patterns exhibit a marked preference for substitution at the internal positions, whereas external positions are only rarely modified. The first pattern corresponds to donor–acceptor pairings (Figure 9a, top), while the second pattern presents moderate electron‐withdrawing substituents placed at the internal positions and donor groups in the adjacent sites (Figure 9a, bottom). Armed with these guidelines, we proceeded to design new anthracenes derivatives by hand. Subsequent validation using TD‐DFT confirmed that the targeted substitution motifs reliably yield compounds meeting the SF criterion (Figure 9b). Notably, this strategy enabled us to predict molecular structures with no prior precedent in the literature, and to identify systematically privileged substitution patterns that govern the emergence of favorable excited‐state behavior.

**FIGURE 9:**
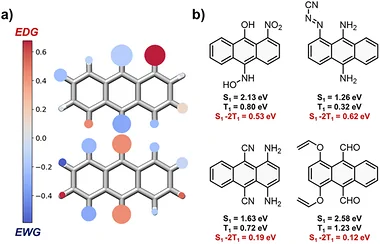
(a) Substituent distribution of candidates proposed in the anthracene core. (b) Hand‐designed anthracene derivatives and the corresponding S_1_ and T_1_ DFT computed values.

## 3 Conclusions

In this work, we have developed and validated an AI‐driven inverse design protocol capable of accurately predicting excited‐state energies (S_1_ and T_1_) across the acene family. The GRU‐RNN model, validated with TD‐DFT calculations, encodes substituents through Hammett σ constants, providing chemical interpretability and enabling the incorporation of previously unseen functional groups via interpolation. This model, coupled with optimization algorithms such as Genetic Algorithms (GA) and Particle Swarm Optimization (PSO), turns out to be an effective methodology for the exploration of a vast chemical space (≈10^19^ structures) to identify candidates meeting the key singlet fission criterion (S_1_> 2T_1_). Beyond recovering known tetracene and pentacene derivatives, the methodology uncovers viable benzene, naphthalene, and anthracene candidates, showing that strategic functionalization can render previously overlooked cores suitable for SF.

Importantly, the model reveals transferable design rules and privileged substitution patterns, while accommodating synthetic constraints such as substituent number, type, and symmetry. This versatile methodology, freely accessible at https://alba.ugr.es/acene/, can be applied to other molecular scaffolds and photophysical targets, enabling the rational discovery of molecules with tailored properties. Overall, it provides a powerful, interpretable, and user‐friendly tool to accelerate the design of functional materials.

## Author Contributions

**Carlos M. Cruz**: investigation, Writing – review and editing, methodology, formal analysis, data curation. **Juan M. Cuerva**: conceptualization, investigation, funding acquisition, writing – original draft, writing – review and editing, visualization, validation, methodology, formal analysis, project administration, resources, supervision, data curation. **Antonio J. Mota**: methodology, writing – review and editing, investigation. **Delia Miguel**: conceptualization, investigation, funding acquisition, writing – original draft, writing – review and editing, visualization, validation, methodology, formal analysis, project administration, data curation, supervision, resources. **Luis Álvarez de Cienfuegos**: investigation, writing – review and editing, methodology, validation, formal analysis, data curation. **Rafael G. Uceda**: investigation, writing – original draft, writing – review and editing, visualization, validation, methodology, software, formal analysis, data curation. **Sandra Míguez‐Lago**: investigation, methodology, writing – review and editing, formal analysis, data curation. **Boris Pérez‐Cañedo**: investigation, writing – original draft, writing – review and editing, visualization, validation, methodology, software, formal analysis, data curation. **Omar Núñez**: investigation, validation, formal analysis. **Joaquin J. Torres**: writing – review and editing, methodology, validation.

## Conflicts of Interest

The authors declare no conflicts of interest.

## Supporting information




**Supporting File**: advs76828‐sup‐0001‐SuppMat.pdf.

## Data Availability

The data that support the findings of this study are available in the supplementary material of this article.
