# Supplementary material for: Artificial Intelligence‐Driven Inverse Design of Singlet Fission Candidates in the Acene family
Source: Adv Sci (Weinh). 2026 Sep 27;13(59):e76828. doi: 10.1002/advs.76828 (PMC13616294; doi:10.1002/advs.76828)
Supplement: Supplementary file 1 [file ADVS-13-e76828-s001.pdf]

Supporting Information
©Wiley-VCH 2026
69451 Weinheim, Germany

Artificial Intelligence-driven inverse design of singlet fission candidates in the acene family

Rafael G. Uceda,[a][b][c] Boris Pérez-Cañedo,[a][b][c]* Sandra Míguez-Lago,[a][b][c] Carlos M. Cruz,[a][b][c] Joaquín. J. Torres,[c][d] Omar Núñez,[a][b][c] Luis Álvarez de Cienfuegos,[a][b][c][e] Antonio Mota,[b][f] Delia Miguel,[b][c][g]* and Juan M. Cuerva[a][b][c]*

[a] Dr. R. G. Uceda, Dr. B. Pérez-Cañedo, Dr. S. Míguez-Lago, Dr. C. M. Cruz, Omar Núñez, Prof. L. Álvarez de Cienfuegos, Prof. J. M. Cuerva
Departamento de Química Orgánica, Facultad de Ciencias
Universidad de Granada (UGR),
C. U. Fuentenueva, 18071 Granada, Spain
E-mail, bpcanedo@ugr.es, jmcuerva@ugr.es

[b] Unidad de Excelencia de Química Aplicada a la Biomedicina y Medioambiente (UEQ)
Universidad de Granada, 18071 Granada, Spain

[c] Cátedra de Energía y Tecnologías Cuánticas
Universidad de Granada, 18071 Granada, Spain

[d] Prof J. J. Torres
Instituto 'Carlos I' de Física Teórica y Computacional. Departamento de Electromagnetismo y Física de la Materia.
Universidad de Granada (UGR),
C. U. Fuentenueva, 18071 Granada, Spain

[e] Prof. L. Álvarez de Cienfuegos
Instituto de Investigación Biosanitaria.
Avda. Madrid, 15, 18016 Granada (Spain).

[f] Dr. A. J. Mota
Departamento de Química Inorgánica,
Facultad de Ciencias, C. U. Fuentenueva, 18071 Granada, Spain

[g] Dr. D. Miguel
Departamento de Fisicoquímica, UEQ, UGR
Facultad de Farmacia, Avda. Profesor Clavera s/n
C. U. Cartuja, 18071 Granada, Spain
E-mail, dmalvarez@ugr.es

Abstract: In this work, we describe the successful application of an AI-driven inverse design protocol to identify singlet fission (SF) candidates across the acene family, exploring a chemical space of ~10^{19} molecules. Substituent effects are encoded via Hammett σ constants, providing a chemically interpretable and generalizable descriptor for unseen functional groups, enabling highly accurate predictions of excited singlet (S_1) and triplet (T_1) states energies. These predictions are coupled with optimization algorithms, including Genetic Algorithms (GA) and Particle Swarm Optimization (PSO), to efficiently navigate chemical space, optimize S_1 and T_1 energies, and reveal recurring substituent patterns. This approach not only identifies well-known tetracene and pentacene candidates but also uncovers viable benzene, naphthalene, and anthracene derivatives that satisfy SF criteria, which are typically inaccessible via intuition. The methodology, accessible at https://alba.ugr.es/acene/, establishes a versatile and interpretable platform for rational molecular design, enabling the exploration of large chemical spaces and the discovery of compounds with tailored excited-state properties.

Table of Contents

1. Dataset generation. 3
2. Computational Methods. 4
2.1. Recurrent Neural Network Architecture. 4
2.2. Dataset 4
2.3. Training Procedure 5
2.4. Genetic Algorithm Optimization 5
2.4.1. Individual Representation 5
2.4.2. GA parameters 5
2.4.3. Fitness Function 5
2.4.4. Output 6
2.5. Particle Swarm Optimization 6
2.5.1. Conceptual Role of PSO 6
2.5.2. Particle Representation 6
2.5.3. PSO Update Equations 6
2.5.4. PSO Parameters 6
2.6. Summary: Hybrid GA-PSO Workflow 7
3. Model performance 8
3.1. Justification of the GRU-RNN Architecture: Comparison with Transformer-Based Models 13
4. Inverse design 16
4.1. Benzenes 16
4.2. Naphtalenes 16
4.3. Anthracenes 17
4.4. Tetracenes 18
4.5. Pentacenes 19
4.6. Conformational aspects 20
5. Dealing with unseen substituents 22
5.1. Benzenes 22
5.2. Naphtalenes 22
5.3. Anthracenes 23
5.4. Tetracenes 24
5.5. Pentacenes 25
6. PSO 26
6.1. Benzenes: One-dimensional PSO 26
6.2. Anthracene: One-dimensional PSO 28
6.3. Anthracenes: Two-dimensional PSO 30
6.4. Anthracenes: Three-dimensional PSO 32
6.5. Anthracenes: Four-dimensional PSO 34
7. Symmetric compounds 37
8. References. 39

1. Dataset generation.

Acene derivatives were generated using a Python-based automated procedure in which substitution sites on the acene framework were randomly selected for replacement with groups other than hydrogen. After selecting a position, a random substituent from a predefined list was assigned. This list included the following substituents: -F, - -OH, $-NH_2$, $-NO_2$, -CN and $-CH_3$. The procedure was repeated until the desired degree of substitution was achieved, ensuring that each generated position was unique and therefore that no substituent was assigned to the same site more than once within the acene structure.

Once the corresponding compound had been generated, its structure was evaluated using DFT calculations performed with the Gaussian 16 software package.[1] All computations were carried out employing the B3LYP functional[2] and the 6-31G(d) basis set.[3] This methodology has been widely used in the literature for similar systems and has been shown to provide reliable geometries and electronic properties, delivering good agreement with experimental results in recent studies.[4] The optimized structures of both S_0 and T_1 were confirmed as true minima on their respective potential energy surfaces, as no imaginary frequencies were found. Subsequently, 50 vertical electronic transitions were computed from the S_0 optimized geometry.

Finally, for their representation within the artificial intelligence framework, each acene is converted into a vector of appropriate length depending on the number of substitution sites: benzene (6), naphthalene (8), anthracene (10), tetracene (12), and pentacene (14) (Figure S1). The numerical value assigned to each position encodes the substituent present at that site, using its Hammett σ_p constant as the descriptor.[5]

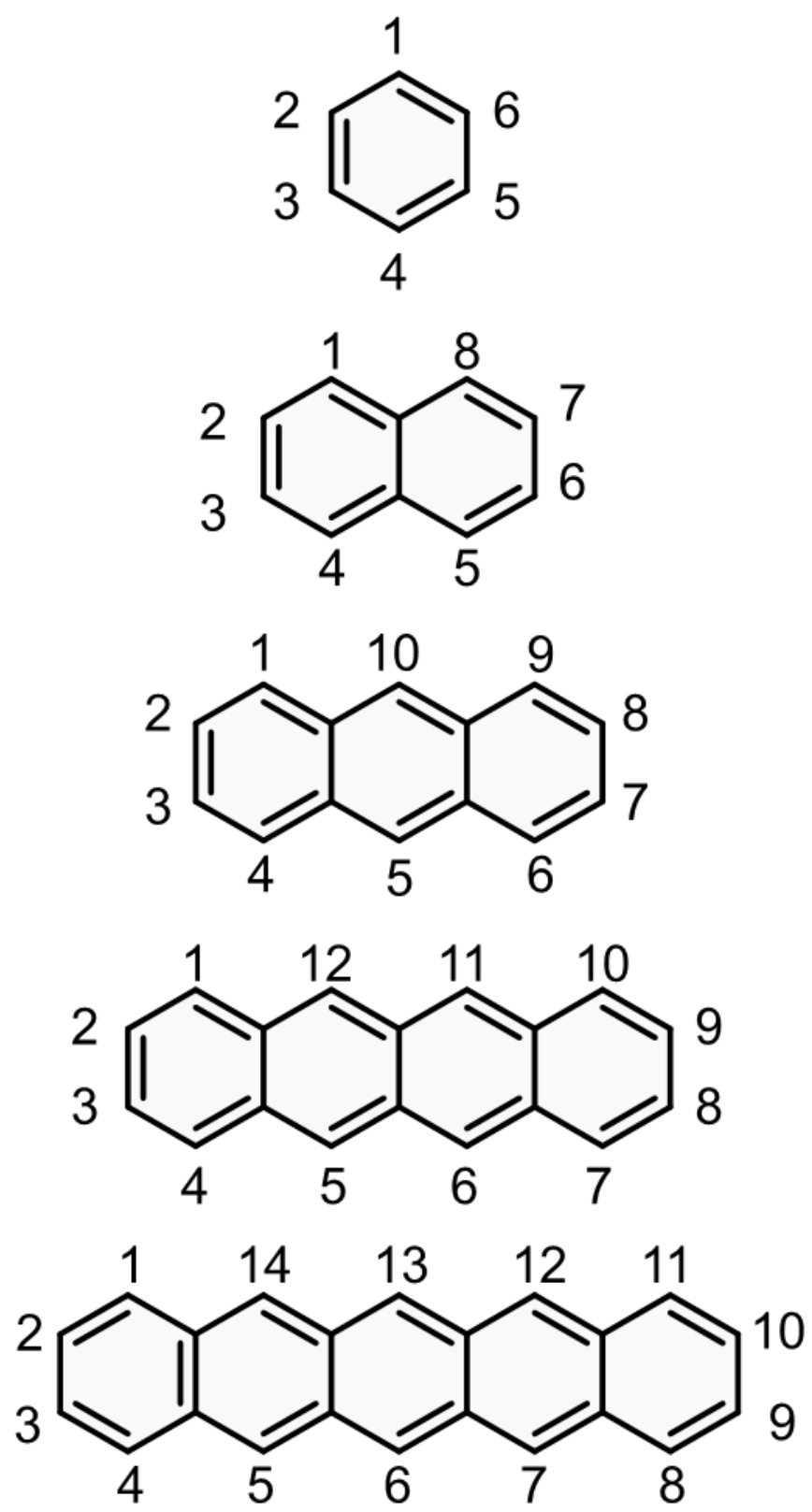

Figure S1. Substitution position numbering as used in this work.

2. Computational Methods.

This section provides a detailed description of the computational methods, algorithms, and source codes used in this work. The Python file RNN.py defines the gated recurrent unit (GRU) neural network architecture employed as a surrogate model for predicting singlet (S_1) and triplet (T_1) excitation energies. The files RNN_Train_S1.py and RNN_Train_T1.py contain the training workflows, data handling, and optimization procedures used to independently train the GRU models for S_1 and T_1, respectively.

The global optimization strategy is implemented in GA.py, which encodes a Genetic Algorithm (GA) designed to explore discrete substitution patterns subject to sparsity constraints, using the trained GRU models as fast evaluators. Local refinement of the GA-derived solutions and alternative solution exploration are performed using Particle Swarm Optimization (PSO), implemented in PSO.py. This file contains the particle representation and velocity–position update equations used to optimize continuous-valued Hammett constants while preserving the substitution topology identified by the GA. Together, these files define a hybrid GA–PSO framework that combines global discrete exploration with local continuous optimization. All script can be found in https://github.com/rguceda/Acenes.git

2.1. Recurrent Neural Network Architecture.

The GRU-based regressor consists of two stacked GRU layers with a hidden size of 32 units, followed by a fully connected linear output layer. Each input sequence x = {x_1, x_2, …, x_L} represents Hammett constants assigned to substitution positions along the acene backbone. Variable-length sequences are handled using sequence packing.

The GRU hidden state update is defined as (see PyTorch's GRU documentation for details):[6]

$$r_t = \sigma\left(W_{ir}x_t + b_{ir} + W_{hr}h_{(t-1)} + b_{hr}\right)$$

$$z_t = \sigma\left(W_{iz}x_t + b_{iz} + W_{hz}h_{(t-1)} + b_{hz}\right)$$

$$n_t = \tanh(W_{in}x_t + b_{in} + r_t \odot (W_{hn}h_{(t-1)} + b_{hn})$$

$$r_t = (1 - z_t) \odot n_t + z_t \odot h_{(t-1)})$$

The final prediction is obtained from the last valid hidden state hL using a linear layer:[7]

$$y = xA^T + b$$

To enforce invariance with respect to molecular symmetries, the model explicitly accounts for all automorphisms of the molecular graph. For each molecule, all symmetry-equivalent representations are generated by applying permutations corresponding to the automorphism group of the acene structure. For benzene molecules, the full dihedral symmetry group is considered, yielding 12 equivalent sequence permutations (six rotations and six reflections). For linear acenes, four symmetry operations are used, corresponding to identity, 180° rotation, and reflections along the long and short molecular axes. Each symmetry-equivalent sequence is processed independently by the model with shared parameters. The final prediction for a molecule is obtained by averaging the outputs over all its symmetry-related representations.

For clarity, we provide the full list of RNN parameters below:

- RNN Type: GRU.
- Number of layers: 2.
- Hidden size: 32.
- Input size: 1.
- Output Layer: Linear.
- Epochs: 500.
- Batch Size: 32.
- Optimizer: Adam.
- Learning Rate: 0.001

2.2. Dataset

CSV files are used as input data, where each row corresponds to one molecular structure. Columns labeled Pos_i represent Hammett constants, and the target column corresponds to either S_1 or T_1 excitation energies. The dataset is randomly split into 80%

training set and 20% testing set. Random seeds are fixed to ensure reproducibility. Mini-batches of size 32 are constructed using padded sequences.

2.3. Training Procedure

The loss function is the mean squared error (MSE):

$$MSE = \frac{1}{N}\sum_i (\hat{y}_i - y_i)^2$$

where y_i is the DFT-computed value (S_1 or T_1) and $\hat{y}_i$ is the model's prediction. Optimization is performed using the Adam optimizer with a learning rate of 0.001 and the optimizer's default parameters (see PyTorch's Adam optimizer documentation for details).[8] Training is carried out for 500 epochs.

2.4. Genetic Algorithm Optimization

GA is a population-based stochastic optimization method inspired by natural evolution. GA propagates information through genetic operators such as crossover and mutation.

We implemented a GA using the Distributed Evolutionary Algorithms in Python (DEAP) framework.[9]

2.4.1. Individual Representation

In the GA used in this work, each candidate solution is represented as a fixed-length vector x = (x_1, x_2, …, x_L), where L corresponds to the number of substitution positions along the acene backbone. Each element xi can take either the value zero (no substitution) or a discrete Hammett constant selected from the set {0.0, 0.06, 0.66, -0.66, -0.17, -0.37, 0.78, -0.27, 0.45, 0.54, 0.42}.

A sparsity constraint is enforced by limiting the number of nonzero elements to at most four, since, currently, synthesizable molecules hardly contain more substituents, and acenes with a large number of substituents might be less appealing to experimentalists.

2.4.2. GA parameters

- Population size: 500

- Number of generations: 5

- Crossover probability (cxpb): 0.9

- Mutation probability (mutpb): 0.7

- Tournament size: 2

Single-point crossover is applied, followed by a repair step enforcing substituent constraints. Mutation replaces Hammett constants with alternative values with probability p = 0.3 per gene.

2.4.3. Fitness Function

The fitness function penalizes structures that violate the Singlet Fission (SF) condition:

$$S_1 \geq 2T_1$$

The fitness value F of a substitution pattern x is defined as:

$$F(x) = \max\big(0, 2T_1(x) - S_1(x)\big)$$

Lower fitness values correspond to better candidates.

2.4.4. Output

The best candidates are stored in a Hall of Fame with size 10 and reported together with predicted S_1 and T_1 values.

2.5. Particle Swarm Optimization

PSO is a population-based, stochastic optimization algorithm inspired by the collective behaviour of social organisms such as bird flocks and fish schools. In PSO, a group of candidate solutions called particles is initialized in the search space. Each particle represents a potential solution and is characterized by its position and velocity. During each iteration, particles update their velocities based on their own best-known position (personal best) and the best-known position found by the entire swarm (global best). In this work, we used the PSO implementation provided by the DEAP framework.[10]

2.5.1. Conceptual Role of PSO

PSO is applied after GA convergence as a local refinement strategy. While the GA explores the discrete combinatorial space of substitution patterns, PSO refines selected nonzero Hammett constants in a continuous search space, thereby improving solutions found by the GA or proposing alternative candidates that are expected to fulfill the SF condition.

2.5.2. Particle Representation

Each particle represents a vector of continuous values corresponding to a subset of nonzero substitution positions identified by the GA:

$$p = (x_1, x_2, \dots, x_d)$$

where d is the number of positions allowed to vary during PSO. In this work, $d \in \{1, 2, 3, 4\}$.

2.5.3. PSO Update Equations

Particle velocities and positions are updated according to:

$$v_i(t+1) = v_i(t) + \varphi_i r_i \odot \left(p_i^{best} - p_i(t)\right) + \varphi^2 r^2 \odot \left(g^{best} - p_i(t)\right)$$

$$p_i(t+1) = p_i(t) + v_i(t+1)$$

where $\varphi_1 = \varphi_2 = 2.0$ are the cognitive and social acceleration coefficients, r_1 and r_2 are vectors of uniformly distributed random numbers in [0,1], p_i^{best} is the best position previously visited by particle i, g^{best} is the global best position, and $\odot$ denotes element-wise multiplication. Velocity magnitudes are clamped to the interval [−0.1, 0.1] to ensure numerical stability.

2.5.4. PSO Parameters

-Swarm size: 50

-Number of generations: 100

-Position bounds: Minimum and maximum Hammett constants

-Velocity bounds: [−0.1, 0.1]

-Objective: Maximize −F(x), equivalent to minimizing the penalty function

2.6. Summary: Hybrid GA-PSO Workflow

GA Stage

1. Initialize a GA population of substitution patterns.

2. Evolve the population using GA operators and surrogate evaluations using the GRU regressors for S_1 and T_1.

3. Extract elite solutions from the GA hall of fame.

PSO Stage

4. For each solution in the hall of fame:

4.1. Output GA solution.

4.2 Apply PSO to the GA solution to refine selected substitution positions using the GRU regressors for S_1 and T_1 as surrogate models.

4.3. Output the corresponding PSO solutions.

3. Model performance

In addition to the graphs presented in Figure 3 of the manuscript, we provide here the percentage errors in the estimation of S_1 and T_1 in the acene derivatives. The data shown in Figure S2 correspond to the test set and illustrate that the percentage error is very low in almost all cases. The exception occurs for low property values, where a small change of 0.1–0.2 eV can result in a large percentage variation; however, this does not imply a conceptual difference.

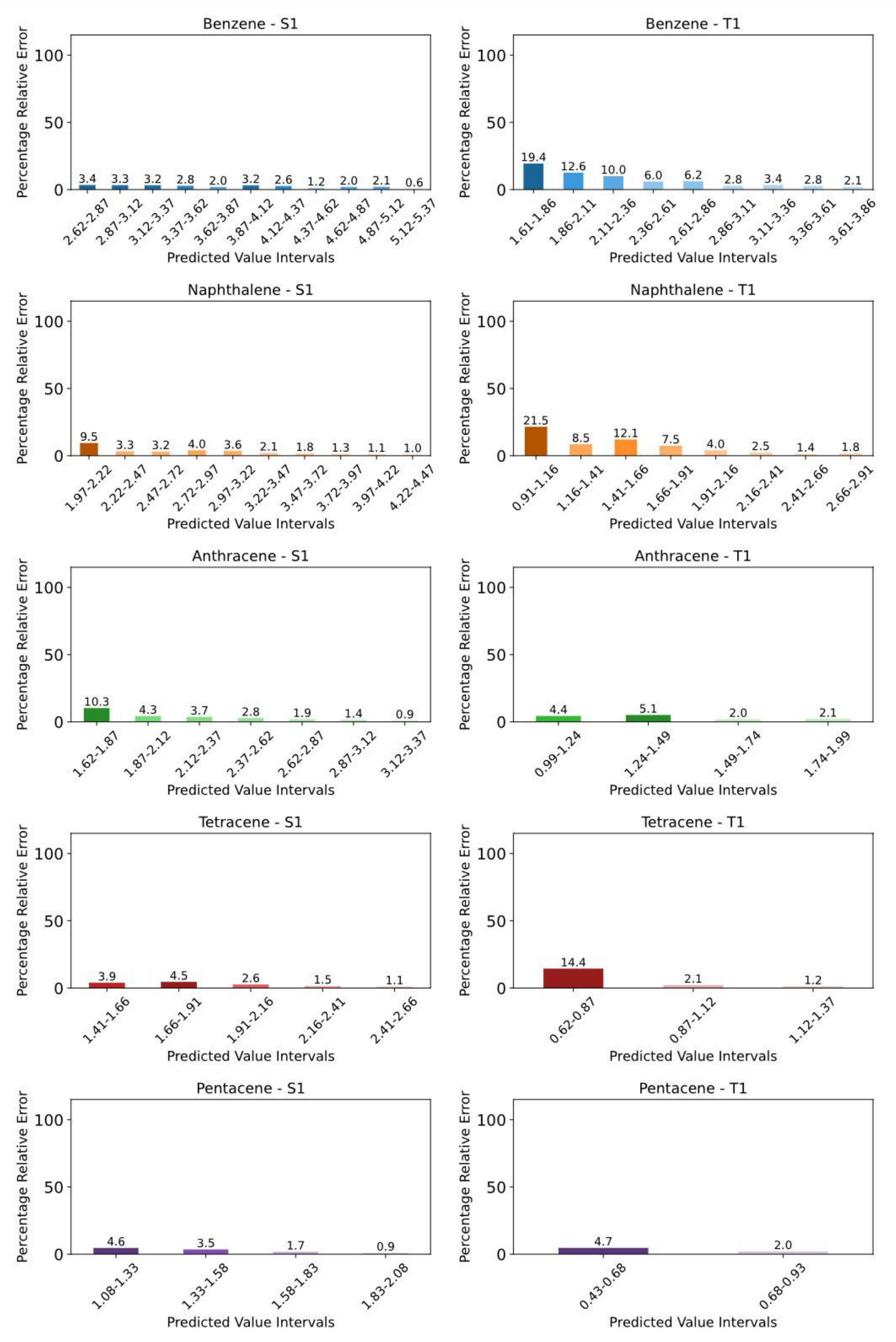

Figure S2. Percentage errors in the estimation of S_1 and T_1 for the test set.

Moreover, although the models were trained with up to four substituents, they exhibit acceptable metrics for evaluating acenes with five (Figure S3) and six (Figure S4) substituents. For this evaluation, 100 acenes with five and six substituents were randomly selected, and the resulting errors were relatively moderate (Figures S5 and S6, respectively), demonstrating that the proposed model can generalize beyond its training set.

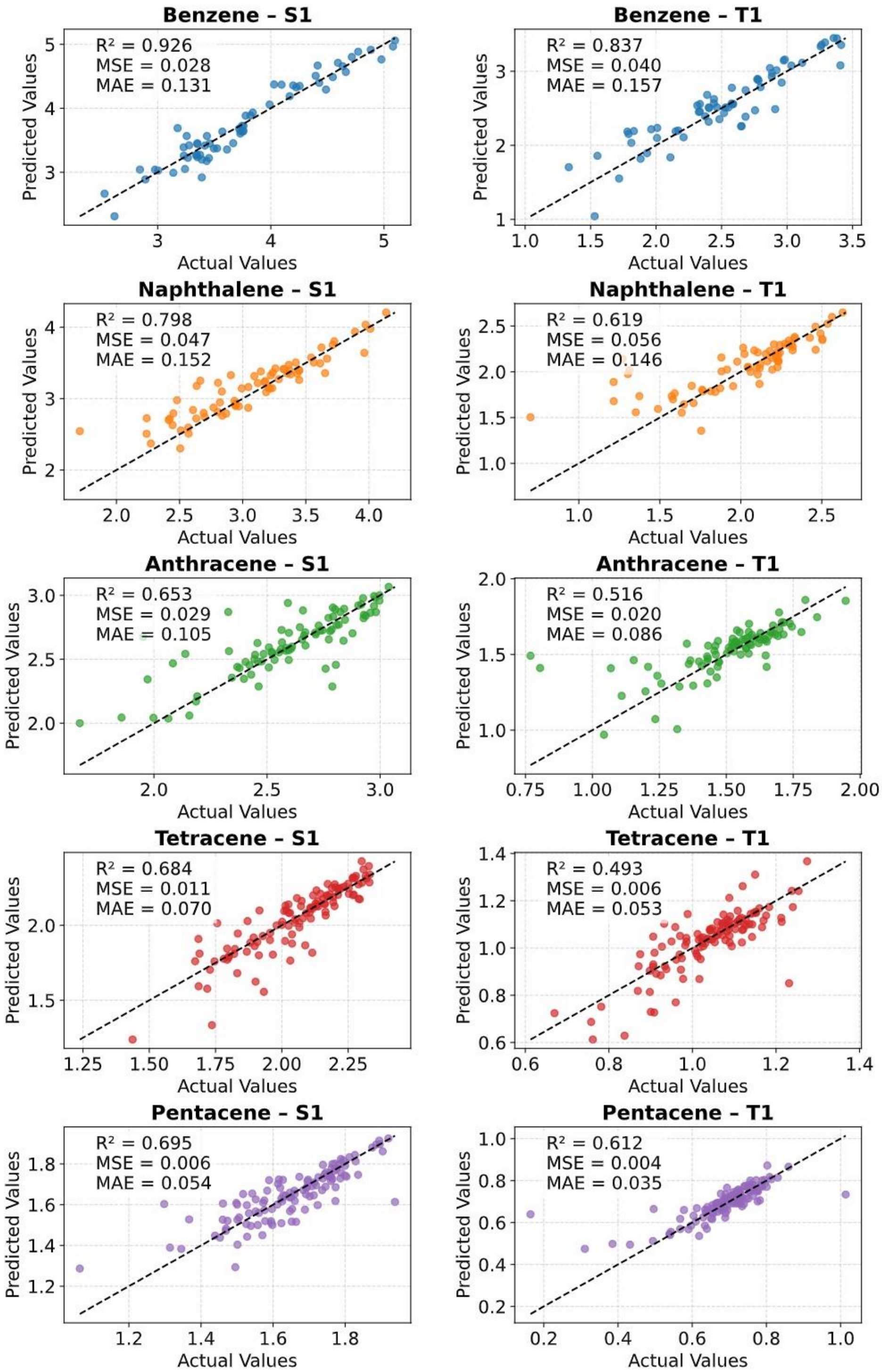

Figure S3. Regression of the target properties S_1 and T_1 for the A_1-A_5 acene family with 5 substituents. The Mean Absolute Error (MAE) and Mean Squared Error (MSE) metrics are computed over the entire set of points.

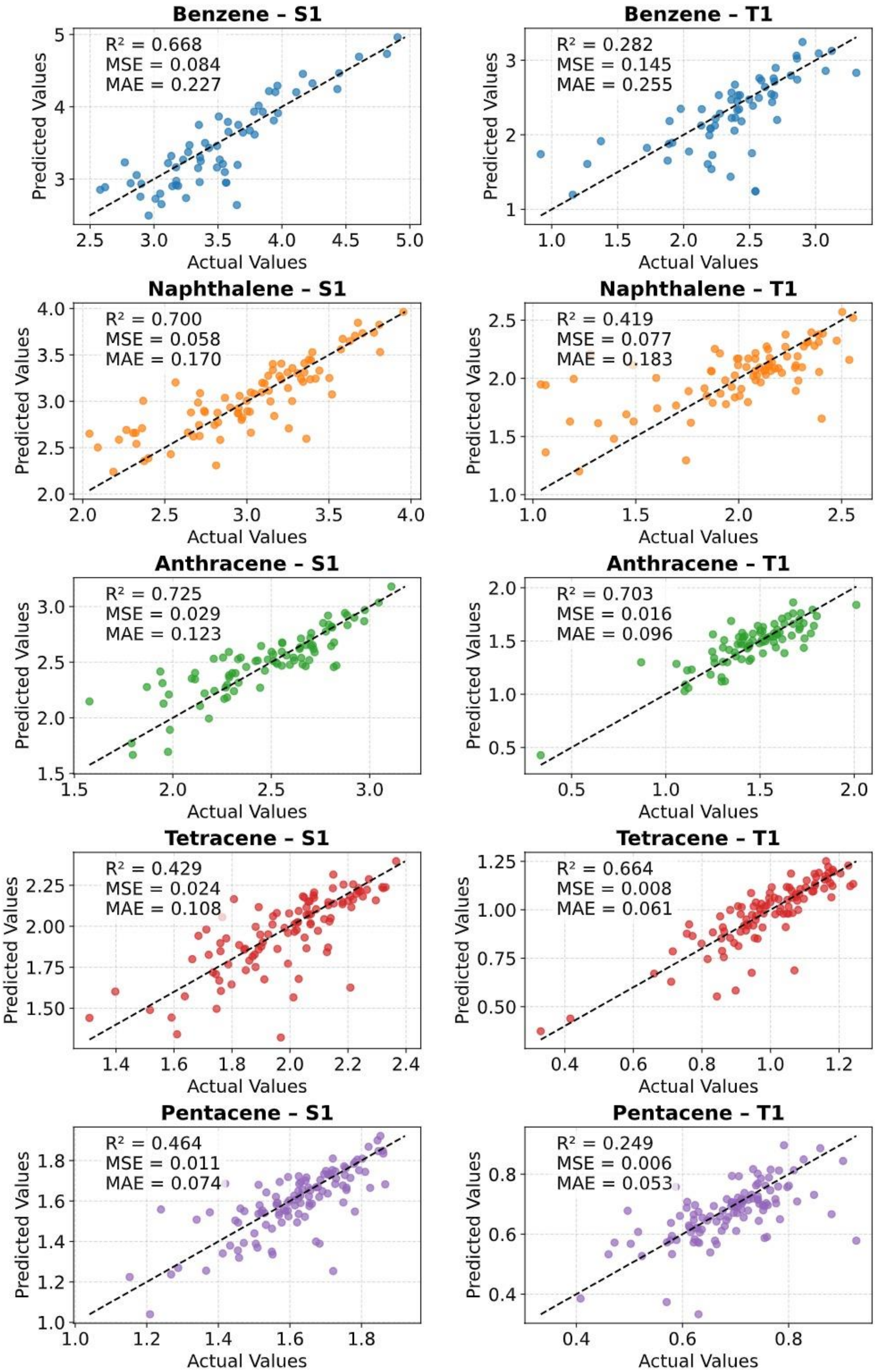

Figure S4. Regression of the target properties S_1 and T_1 for the A_1-A_5 acene family with 6 substituents. The Mean Absolute Error (MAE) and Mean Squared Error (MSE) metrics are computed over the entire set of points.

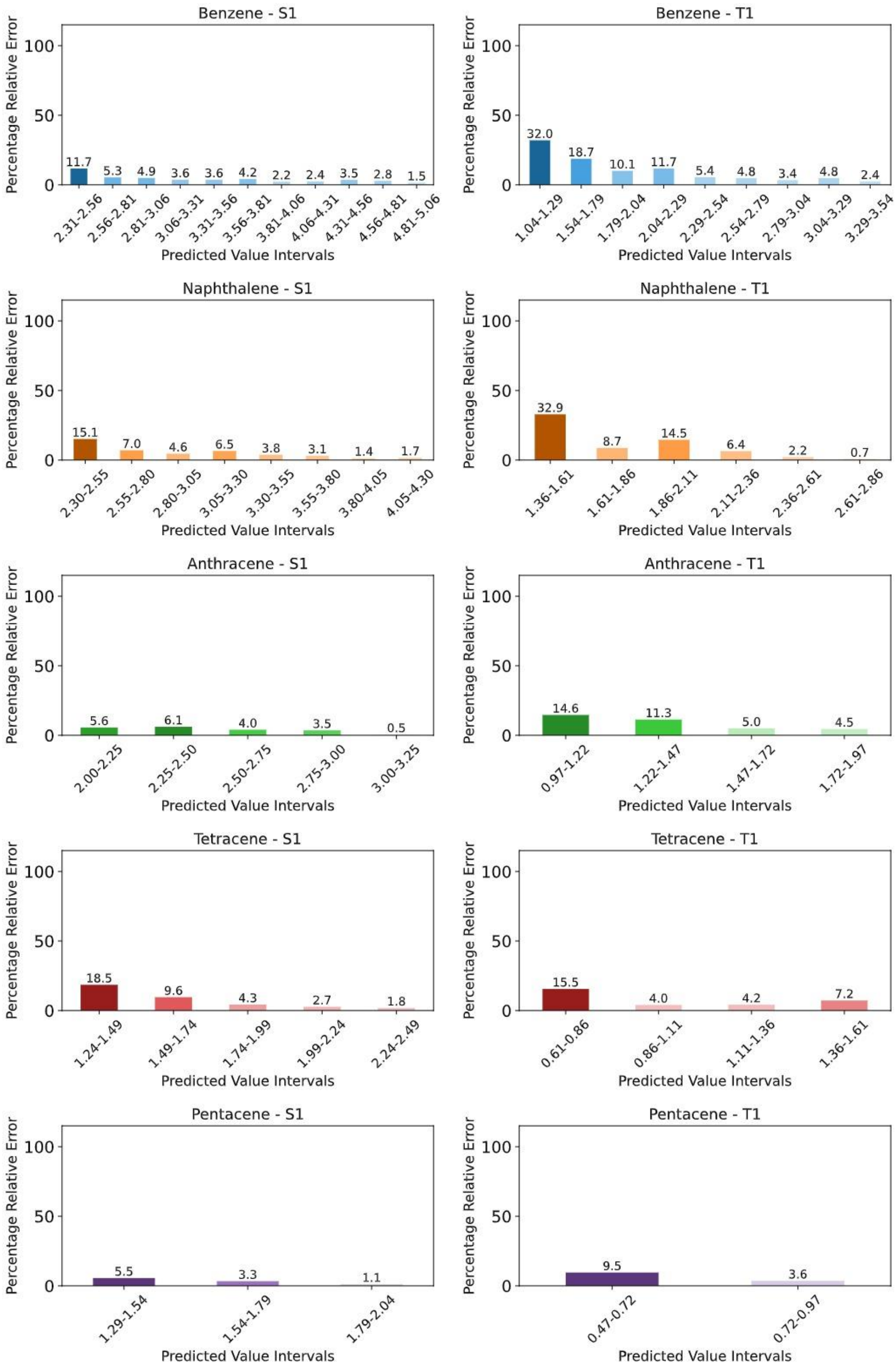

Figure S5. Percentage errors in the estimation of S_1 and T_1 in acenes with 5 substituents.

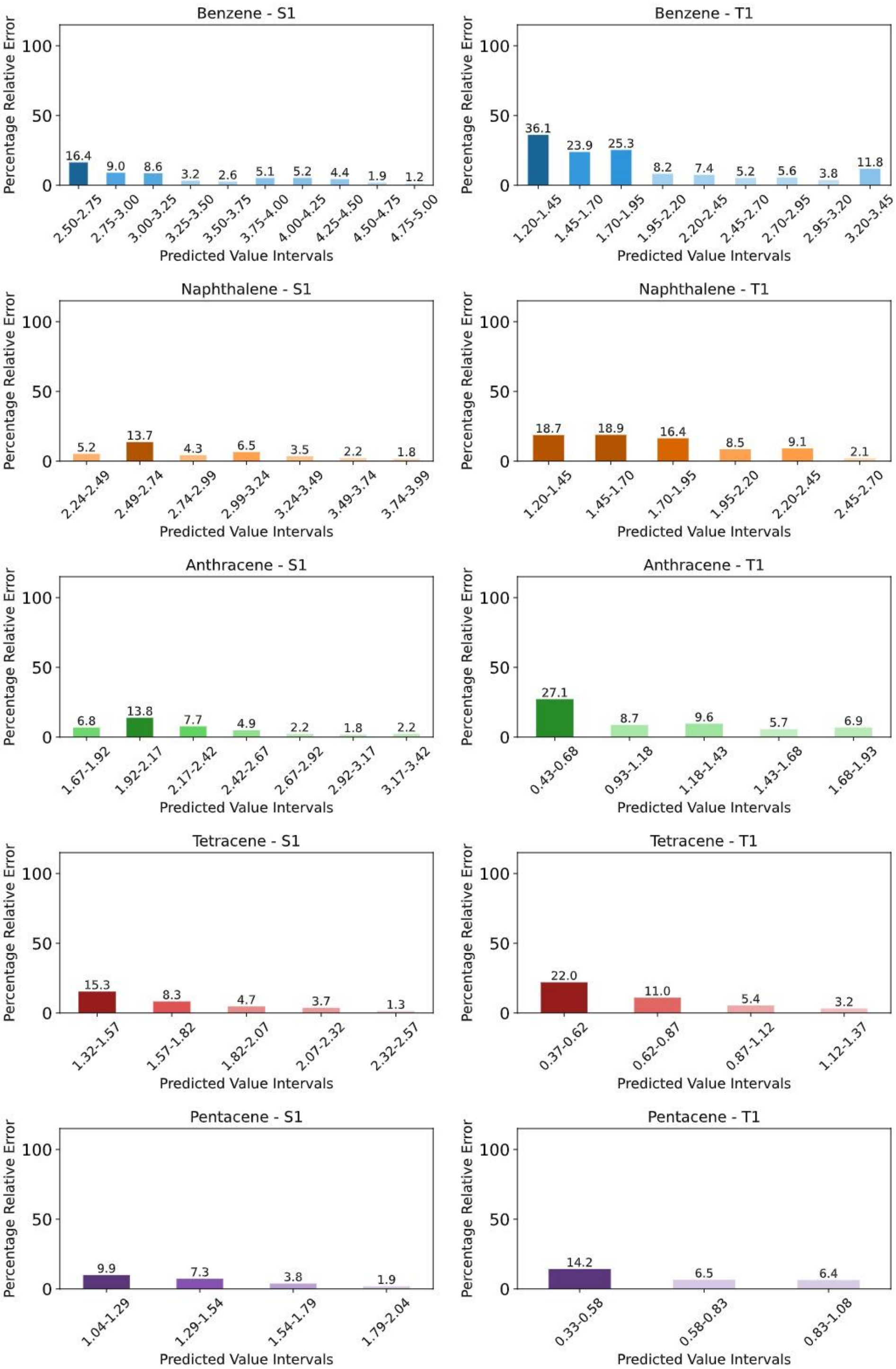

Figure S6. Percentage errors in the estimation of S_1 and T_1 in acenes with 6 substituents.

3.1. Justification of the GRU-RNN Architecture: Comparison with Transformer-Based Models

During the peer-review process, one reviewer suggested evaluating transformer-based models as an alternative to the proposed GRU-RNN framework, arguing that attention-based architectures generally scale more effectively with increasing data volumes and provide a powerful paradigm for conditional molecular generation. To rigorously assess this possibility, we performed a direct benchmarking study using the Regression Transformer architecture reported by Born and co-workers.[11]

The regression transformer model was implemented using the codebase provided by IBM at the GitHub repository: https://github.com/IBM/regression-transformer.git, following the framework described in *Nat. Mach. Intell.* **2023**, *5*, 432-444.

Training Dataset

The training set was constructed from five classes of linear acenes and consisted of:

- 1,000 benzene derivatives,
- 1,000 naphthalene derivatives,
- 1,000 anthracene derivatives,
- 1,000 tetracene derivatives, and
- 1,000 pentacene derivatives.

Test Datasets

Separate test sets were assembled for singlet (S_1) and triplet (T_1) excitation energy prediction. The size of each test set depended on the availability of data:

- Benzenes: 259 (S_1), 177 (T_1)
- Naphthalenes: 1,000 (S_1), 1,000 (T_1)
- Anthracenes: 1,000 (S_1), 1,000 (T_1)
- Tetracenes: 800 (S_1), 667 (T_1)
- Pentacenes: 1,000 (S_1), 1,000 (T_1)

Model Architecture

The regression transformer architecture employed the following hyperparameters:

- d_model = 128
- d_inner = 512
- d_head = 16
- n_head = 8
- n_layer = 8
- numerical_encodings_dim = 16

Preliminary experiments indicated that smaller architectures were unable to adequately learn the structure-property relationships present in the dataset. Consequently, the above configuration was adopted for all reported experiments.

Training Protocol

For experiments involving the molecular-family-specific datasets, models were trained using:

- learning_rate = 1×10^{-3}
- epochs = 500
- batch_size = 32

All remaining hyperparameters and training options were kept at their default values as specified in the original implementation.

Baseline GRU-based RNN Model

To provide a benchmark for comparison, the proposed GRU-based RNN model was trained and evaluated alongside the regression transformer. The GRU-based RNN was trained using the same training datasets employed for the regression transformer, namely the 1,000-sample datasets for each molecular family (benzenes, naphthalenes, anthracenes, tetracenes, and pentacenes). Evaluation was performed using the same test datasets described above for both S_1 and T_1 excitation energy prediction tasks.

The predictive performance of both models was assessed on the corresponding test sets. Under these conditions, the GRU-based RNN model consistently achieved superior predictive performance compared with the regression transformer.

Results

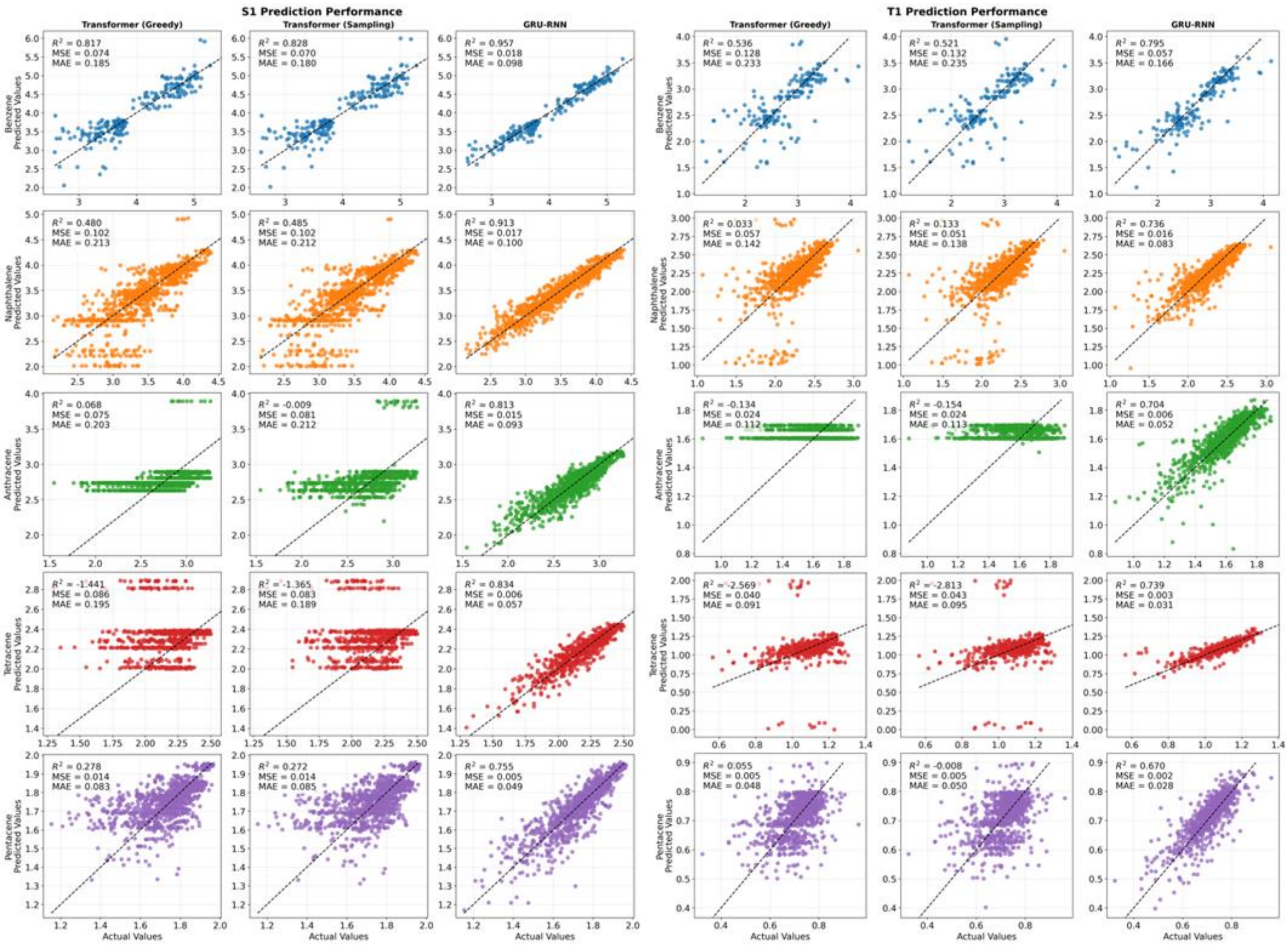

Figure S7. Excited state energy predictions in the test set using the regression transformer and the proposed GRU-RNN.

Full-Dataset Training

To investigate whether access to larger amounts of training data could improve the performance of the regression transformer, additional experiments were conducted using the complete available dataset. In this case, the configuration was modified to:

- learning_rate = 1×10^{-3}
- epochs = 1000
- batch_size = 100

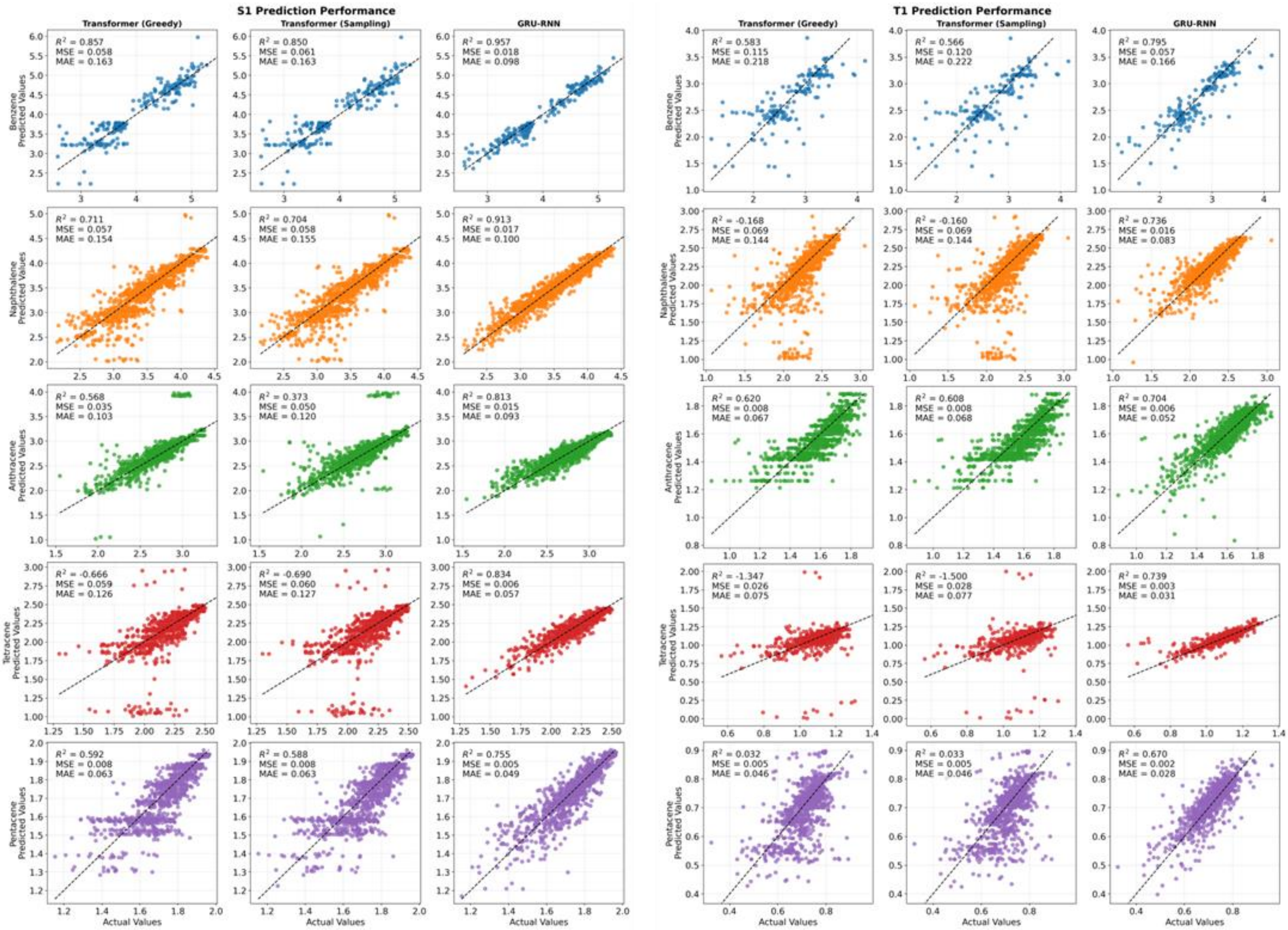

Figure S8. Excited state energy predictions in the test set using the regression transformer and the proposed GRU-RNN. The full training set was used to train the transformer model. The test set remains the same. We keep the metrics attained by the GRU-RNN model for reference.

We see that indeed the regression transformer metrics improve with more training data. For comparison, the performance metrics of the GRU-based RNN model were retained. Despite being trained on less data, the GRU-based RNN continued to outperform the regression transformer on the common test sets. These results suggest that, within the computational budget available for this study, increasing the amount of training data alone was insufficient for the regression transformer to surpass the performance of the GRU-based RNN.

4. Inverse design

4.1. Benzenes

Table S1. TD-DFT values of candidates obtained through inverse design.

Substituents	S_1/eV	T_1/eV	S_1-2T_1/eV
None (Benzene)	5.54	3.88	-2.22
1-NO_2_2-OH_3-NH_2_4-OH	3.73	1.60	0.53
1-NO_2_2-OH_3-NH_2_4-NH_2_5-F	3.03	1.56	-0.09
1-NO_2_2-OH_3-NH_2_4-NH_2	3.08	1.61	-0.14

4.2. Naphtalenes

Table S2. TD-DFT values of candidates obtained through inverse design.

Substituents	S_1/eV	T_1/eV	S_1-2T_1/eV
None (Naphthalene)	4.46	2.72	-0.98
1-OH_4-NH_2_5-NO_2_8-NO_2	1.90	0.78	0.34
1-OH_4-CH_3_5-CH_3_8-NO_2	2.66	1.23	0.20
1-NO_2_2-CN_4-CN_8-OH	2.52	1.21	0.10
1-NO_2_2-CH_3_4-F_8-OH	2.74	1.33	0.08
1-NH_2_4-NO_2_5-OH_8-CN	2.96	1.45	0.06

4.3. Anthracenes

Table S3. TD-DFT values of candidates obtained through inverse design.

**Substituents**	**S_1/eV**	**T_1/eV**	**S_1-2T_1/eV**
None (Anthracene)	3.27	1.81	-0.35
1-NO_2_2-CH_3_9-CH_3_10-OH	2.14	0.82	0.50
2-NH_2_4-NO_2_5-OH_6-CH_3	1.88	0.71	0.46
5-OH_6-NO_2_7-CN_10-CH_3	1.98	0.78	0.42
5-CH_3_9-NO_2_10-OH	2.05	0.83	0.39
5-OH_9-NO_2_10-NH_2	2.34	0.97	0.40
2-CH_3_6-CH_3_9-NO_2_10-OH	2.13	0.92	0.29
2-CN_5-OH_6-NO_2	2.21	0.97	0.27
1-NH_2_4-NH_2_9-CH3_10-NO_2	2.18	1.02	0.14
1-CN_5- NH_2_6 NH_2_9-CN_10- NH_2	2.19	1.06	0.07
5-NO_2_6-OH_10-CN	2.28	1.18	-0.08
1-NH_2_4-CH_3_5-NO_2_10-CN	2.23	1.16	-0.09
4-NH_2_5-CN_6-CH_3_10-NH_2	2.60	1.37	-0.14

4.4. Tetracenes

Table S4. TD-DFT values of candidates obtained through inverse design.

**Substituents**	**S_1/eV**	**T_1/eV**	**S_1-2T_1/eV**
None (Tetracene)	2.49	1.20	0.09
4-F_5-NO_2_6-F_12-NH_2	2.28	1.07	0.14
1-OH_12-NO_2	2.05	0.97	0.11
12-NH_2	2.27	1.08	0.11
11-CN	2.36	1.13	0.10
2-F	2.49	1.20	0.09
1-CH_3_9-NH_2_11-NH_2	2.19	1.06	0.07
1-CH_3_11-F	2.47	1.20	0.07
4-CH_3	2.50	1.22	0.06
1-CH_3	2.50	1.22	0.06
1-OH_5-F	2.43	1.19	0.05
10-F_11-OH	2.41	1.18	0.05
3-NH_2_11-NH_2	2.25	1.10	0.05
2-OH_3-F	2.48	1.22	0.04
9-NH_2_12-F	2.37	1.17	0.03
2-CN_3-F_4-NH_2	2.30	1.14	0.02
2-CN_3-F_8-NO_2_9-F	2.20	1.09	0.02
5-NO_2_11-F	2.24	1.12	0.00
3-CN_4-NH_2_6-CH_3_8-NH_2	2.26	1.16	-0.06
1-CN_4-CH_3_10-NH_2_12-CH_3	2.16	1.12	-0.08

4.5. Pentacenes

Table S5. TD-DFT values of candidates obtained through inverse design.

**Substituents**	**S_1/eV**	**T_1/eV**	**S_1-$2T_1$/eV**
None (Pentacene)	1.94	0.78	0.38
3-NH_2_7-NH_2	1.78	0.72	0.34
6-NO_2	1.83	0.75	0.33
11-NH_2	1.88	0.78	0.32
1-CH_3_3-CH_3_11-F_12-CN	1.80	0.75	0.30
3-CN_14-CN	1.74	0.72	0.30
2-F_9-CH_3_14-CN	1.83	0.77	0.29
2-NH_2_10-OH	1.89	0.80	0.29
2-NH_2_9-NO_2_11-CH_3_13-NH_2	1.56	0.64	0.28
2-NH_2_5-NH_2_6-NH_2_10-F	1.63	0.69	0.25
2-NH_2_3-NO_2_10-CH_3	1.45	0.62	0.21
4-CH_3_9-F_10-NO_2_11-NO_2	1.57	0.69	0.19
1-NH_2_3-OH_5-NO_2_7-F	1.77	0.80	0.17
3-CH_3_4-NH_2_10-NO_2	1.61	0.72	0.17
1-NH_2_2-F_4-CH_3_12-NO_2	1.64	0.74	0.16
4-OH_5-NH_2_9-OH_12-NO_2	1.52	0.68	0.16
1-OH_10-NH_2_12-NO_2	1.76	0.80	0.16
2-NH_2_4-CH_3_11-NO_2	1.61	0.75	0.11
3-NH_2_8-NO_2_9-NO_2_10-CN	1.45	0.68	0.09
2-F_4-NO_2_5-OH_7-OH	1.27	0.59	0.09
1-OH_2-NO_2_3-CH_3_9-NH_2	1.73	0.84	0.05
1-F_3-NH_2_8-CN_11-NO_2	1.34	0.65	0.04
1-NH_2_3-OH_10-NH_2_11-NO_2	1.77	0.89	-0.01

4.6. Conformational aspects

To evaluate the impact of conformational flexibility on the computed excited-state properties, conformational searches were performed using CREST[12] for all candidates obtained through the inverse-design procedure. For each molecule, the relevant conformers were identified and their corresponding TD-DFT energy gaps were calculated. The resulting values for the candidates shown in Figure 4, reported in Table S6, exhibit excellent agreement between the properties derived from the most populated conformer and those obtained from the full conformational ensemble. This indicates that a single dominant conformer largely governs the Boltzmann-weighted description, and therefore provides a reliable representation of the electronic properties of the system.

Table S6. TD-DFT-computed properties of candidates obtained through the inverse-design workflow, including values for the most populated conformer and the full conformational ensemble.

Molecule	Number of relevant conformers	S_1-$2T_1$ (most populated conformer) / eV	S_1-$2T_1$ (ensemble) / eV
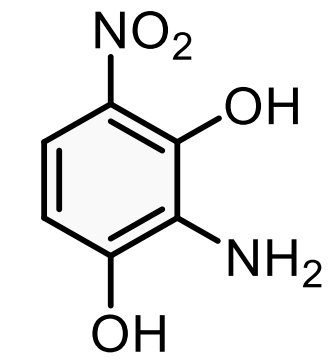	2	0.53	0.53
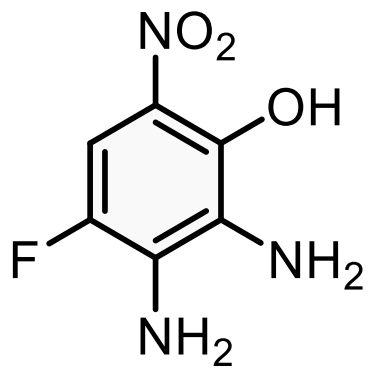	1	-0.09	-0.09
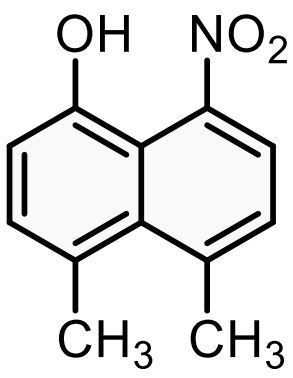	2	0.20	0.20
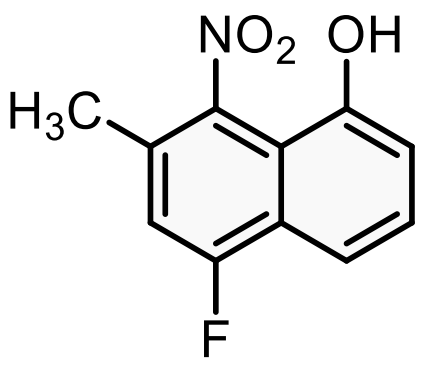	2	0.08	0.07
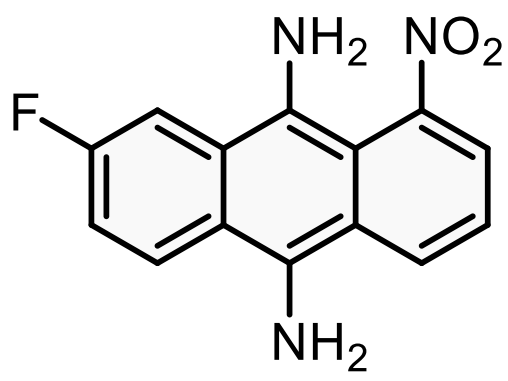	4	0.10	0.11
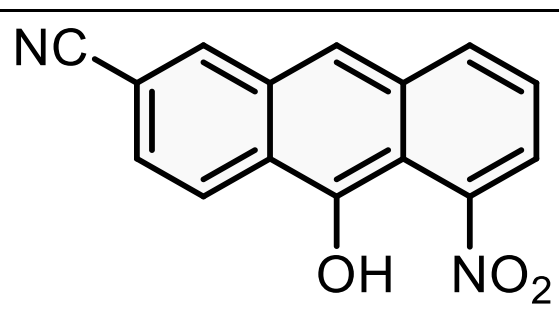	2	0.27	0.27
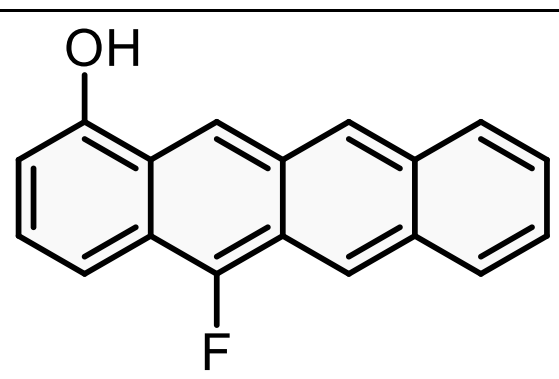	2	0.05	0.05

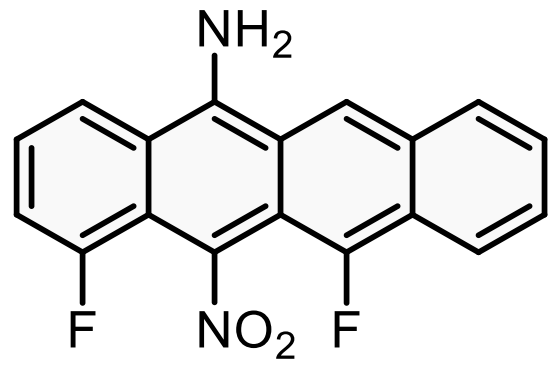	2	0.14	0.14
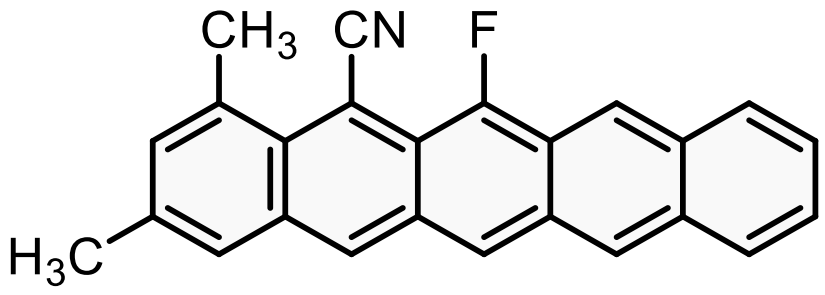	1	0.30	0.30
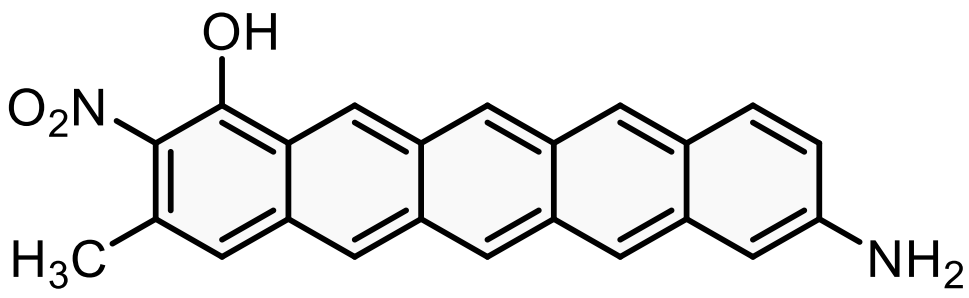	1	0.05	0.05

This behaviour is not limited to systems with a small number of conformers, but is also observed in compounds exhibiting more complex substituents and a higher degree of conformational diversity. As an illustrative example, Table S7 reports an analogous analysis to that presented for the molecules shown in Figure 9, which are particularly relevant as they were obtained through manual design following the guidelines established throughout this work.

Table S7. TD-DFT-computed properties and conformational analysis of manually designed candidates and comparison with conformational ensembles.

Molecule	Number of relevant conformers	S_1-$2T_1$ (most populated conformer) / eV	S_1-$2T_1$ (ensemble) / eV
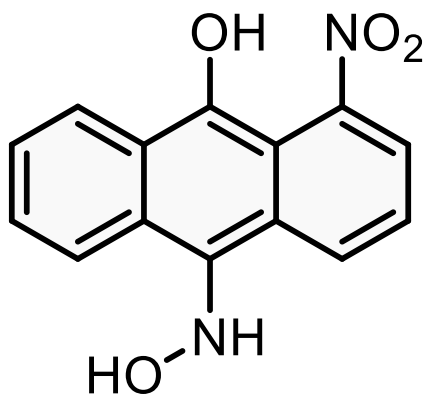	19	0.53	0.48
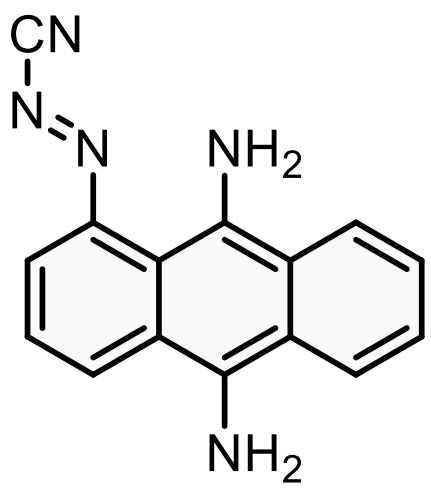	8	0.62	0.62
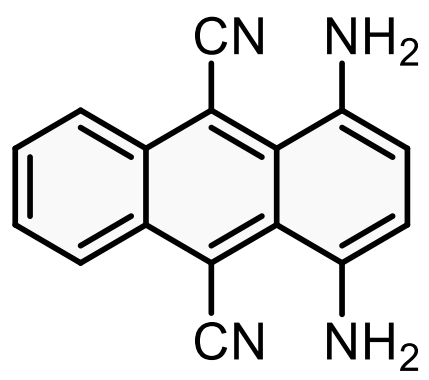	1	0.19	0.19
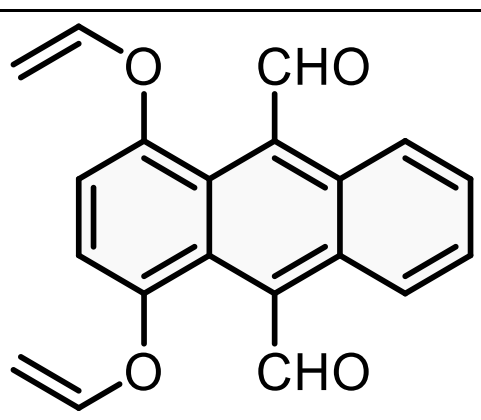	16	0.12	0.05

5. Dealing with unseen substituents

In this section, a set of candidate structures has been explored, comprising the six original substituents (–F, $–NH_2$, –OH, $–CH_3$, –CN, $–NO_2$) together with four additional ones ($–CF_3$, –OMe, –CHO and –COOH). Many of the candidates identified incorporate at least one of the newly introduced substituents, whereas others are restricted to the original set. Since all candidates were obtained using the genetic algorithm and satisfy the imposed constraints—given that the six original substituents are included within the overall pool—they are all reported here. The newly introduced substituents, which were not encountered by the model at any stage of training or optimization, are highlighted in red.

5.1. Benzenes

Table S8. TD-DFT values of candidates obtained through inverse design.

Substituents	S_1/eV	T_1/eV	S_1-2T_1/eV
None (Benzene)	5.54	3.88	-2.22
2-OH_3-NO_2_5-OMe_6-NO_2	3.14	1.57	0.00
1-NO_2_2-OH_5-OMe_6-NO_2	3.29	1.62	0.05

5.2. Naphtalenes

Table S9. TD-DFT values of candidates obtained through inverse design.

Substituents	S_1/eV	T_1/eV	S_1-2T_1/eV
None (Naphthalene)	4.46	2.72	-0.98
1-OH_2-OMe_5-OH_8-NO_2	2.75	1.17	0.41
1-OH_2-OH_5-OH_8-NO_2	2.65	1.12	0.41
1-NO_2_2-COOH_8-OH	2.47	1.15	0.17
1-NO_2_4-CN_6-F_8-OH	2.51	1.26	-0.01
1-NO_2_4-CHO_6-CHO_8-OH	2.48	1.25	-0.02

5.3. Anthracenes

Table S10. TD-DFT values of candidates obtained through inverse design.

Substituents	S_1/eV	T_1/eV	S_1-2T_1/eV
None (Anthracene)	3.27	1.81	-0.35
1-NO_2_4-CN_5-NH_2_10-OH	1.56	0.42	0.72
5-NH_2_9-NO_2_10-OH	1.84	0.63	0.58
1-OH_5-CF_3_7-CF_3_10-COOH	2.71	1.15	0.41
1- NH_2_5-CF_3_6 NH_2_9-NH_2_10-CF_3	1.63	0.65	0.33
1-NO_2_5-OH_9-COOH_10-NH_2	2.34	1.01	0.32
8-CH_3_9-OH_10-NO_2	2.37	1.05	0.27
1-OH_5-CN_7-OMe_10-COOH	2.48	1.14	0.20
1-CF_3_5-CHO_7-CHO_10-NO_2	2.78	1.32	0.14
5-OMe_8-CHO_9-COOH_10-CHO	2.89	1.39	0.11
2-NH_2_5-COOH_6-OH_9-CH_3	2.69	1.33	0.03
3-CN_5-NH_2_8-COOH_10-NH_2	2.31	1.15	0.01
1-F_3-CN_5-CF_3_10-CF_3	2.88	1.45	-0.02
4-F_5-CN_10-CHO	2.64	1.34	-0.04
5-NH2_9-NO_2_10-OMe	2.63	1.34	-0.05
1-CF_3_3-CH_3_5-CF_3_10-CH_3	3.05	1.55	-0.05
1-COOH_10-CHO	2.86	1.47	-0.08
5-CH_3_8-COOH_10-CHO	2.84	1.47	-0.10
3-CF_3_5-CF_3_10-COOH	3.08	1.59	-0.10
5-CHO_8-NH_2_10-NH_2	2.72	1.41	-0.10
4-CF_3_5-OMe_10-CHO	2.90	1.51	-0.12

5.4. Tetracenes

Table S11. TD-DFT values of candidates obtained through inverse design.

Substituents	S_1/eV	T_1/eV	S_1-$2T_1$/eV
None (Tetracene)	2.49	1.20	0.09
4-F_5-NO_2_6-F_12-NH_2	2.28	1.07	0.14
6-NH_2	2.27	1.08	0.11
8-OMe_9-F_11-F	2.47	1.18	0.11
3-OH_10-OH_11-NO_2	2.02	0.96	0.10
9-NH_2_12-NH_2	2.24	1.07	0.10
3-COOH_12-CH_3	2.38	1.15	0.08
8-F_12-F	2.45	1.19	0.07
3-CN_4-CH_3_5-NH_2	2.22	1.07	0.08
4-CH_3_7-OMe_12-NH_2	2.32	1.13	0.06
3-CN	2.40	1.18	0.04
10-CN_11-F	2.35	1.15	0.05
2-COOH_9-COOH_10-OMe_12-F	2.35	1.17	0.01
10-COOH	2.37	1.18	0.01
2-NO_2_8-COOH_11-NO_2	2.26	1.13	0.00

5.5. Pentacenes

Table S12. TD-DFT values of candidates obtained through inverse design.

Substituents	S_1/eV	T_1/eV	S_1-2T_1/eV
None (Pentacene)	1.94	0.78	0.38
2-OMe_3-NH_2_4-NH_2_6-CN	1.65	0.61	0.43
3-NH_2_4-NH_2_9-COOH_12-COOH	1.50	0.56	0.38
2-OMe_6-COOH_7-NH_2_11-NO_2	1.54	0.58	0.38
6-COOH	1.88	0.75	0.38
1-COOH_12-F_13-CH_3	1.80	0.71	0.38
2-CN_9-CN	1.87	0.76	0.35
1-COOH_2-F_6-F_12-COOH	1.87	0.76	0.35
1-F_6-NO_2_7-OMe_8-NH_2	1.63	0.66	0.31
2-NO_2_11-F	1.80	0.76	0.28
2-CH_3_3-CH_3_12-CN_13-COOH	1.77	0.75	0.27
4-NO_2_6-OH_9-OMe_10-CN	1.55	0.65	0.25
1-NH_2_5-CH_3_10-CN_13-CN	1.56	0.65	0.26
3-OH_5-F_9-NO_2_10-NH_2	1.42	0.60	0.22
1-CN_4-NO_2_5-CH_3_9-COOH	1.64	0.71	0.22
4-OH_8-COOH_9-CN_14-NH_2	1.45	0.64	0.17
1-OMe_2-NH_2_3-NH_2_9-NO_2	1.66	0.77	0.12
1-NH_2_7-NO_2_9-OH_12-NO_2	1.45	0.66	0.13
4-OH_10-NH_2_12-NO_2	1.76	0.82	0.12
3-NO_2_4-NH_2_7-OMe_12-NO_2	1.78	0.84	0.10
4-CH_3_8-NO_2_9-NH_2_14-CH_3	1.87	0.89	0.09
3-OH_4-NO_2_10-CH_3	1.79	0.90	-0.01

6. PSO

Owing to the nature of the substituents involved, this section presents the molecular structures of the proposed candidates that were subsequently validated by TD-DFT calculations. The section is organized as follows: first, the cases related to benzene derivatives are introduced; subsequently, naphthalene-based derivatives are examined in detail, considering both the one-dimensional PSO approach and PSO schemes involving from two to four substitutions.

6.1. Benzenes: One-dimensional PSO

Due to the simple structure of benzene, this case is taken as a reference to explain the proposed protocol. First, the genetic algorithm is used to identify promising benzene candidates; subsequently, particle swarm optimization (PSO) is applied to modify the substituents present in these structures. It is worth noting that the derivatives proposed by the genetic algorithm are very similar to each other (Figure S9, up), and that, once PSO is applied, the substituents are mutated by very similar constants in all cases (Figure S9, bottom). For example, the NO_2 group at position 1 is consistently replaced by a substituent of a σ value around 0.8. This behavior highlights the consistency of the approach and demonstrates that the underlying physics of the system is being interpreted in a unique and coherent manner.

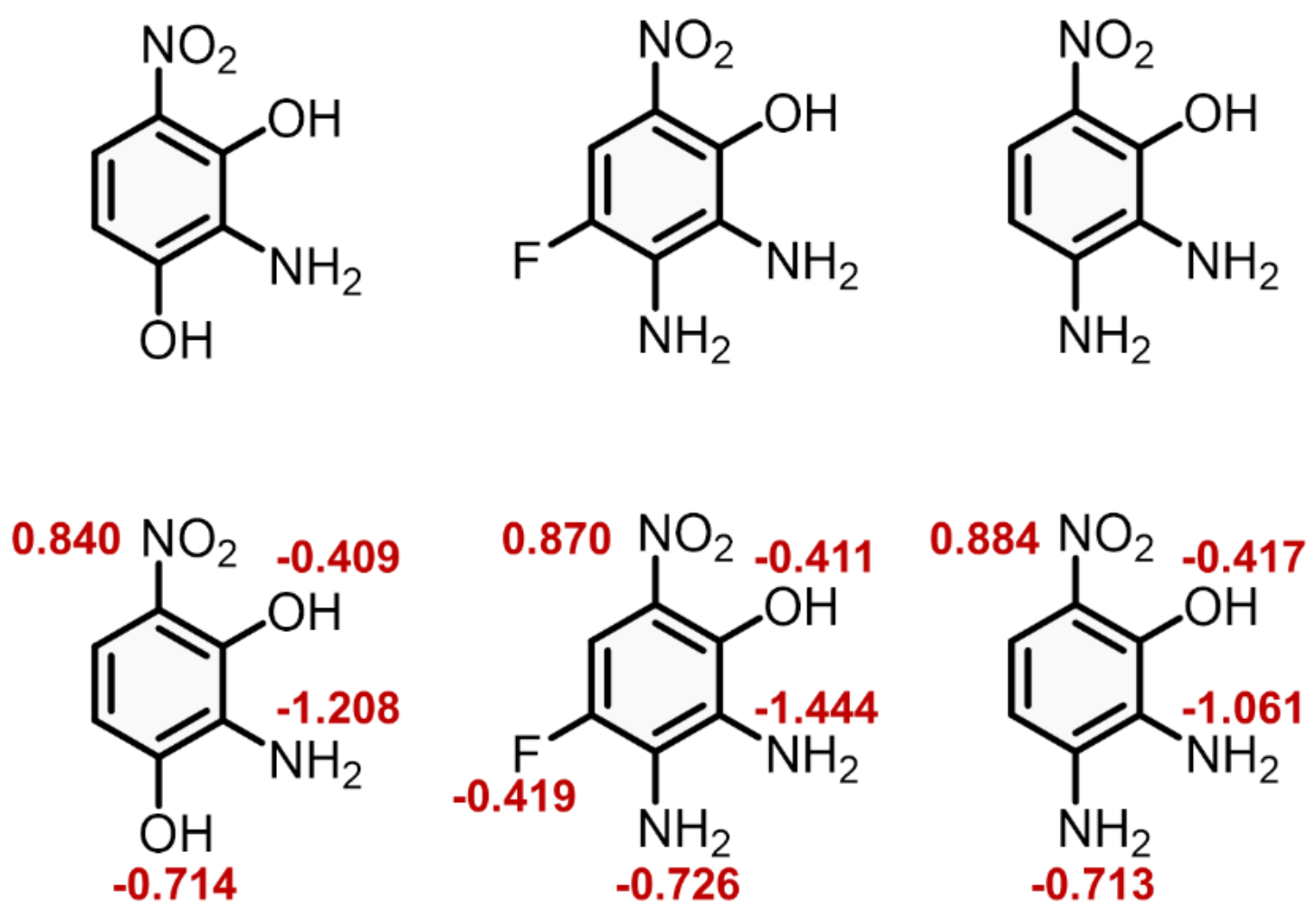

Figure S9. Up: Candidates proposed by the genetic algorithm for benzene derivatives. Bottom: Hammett constants proposed by the PSO for single-substitution modifications of the benzene candidates generated by the GA.

Next, Table S13 presents several benzene derivatives obtained by PSO that satisfy the SF condition. As said, since this case involves substituents of diverse types that have not appeared earlier, the structures are shown explicitly rather than being identified by name. This representation scheme will be maintained throughout Section 5 of this Supporting Information.

Table S13. TD-DFT values of benzene candidates obtained via the GA/PSO combination and one-dimensional PSO.

Parent				Son			
**Structure**	**S_1/eV**	**T_1/eV**	**S_1-2T_1/eV**	**Structure**	**S_1/eV**	**T_1/eV**	**S_1-2T_1/eV**
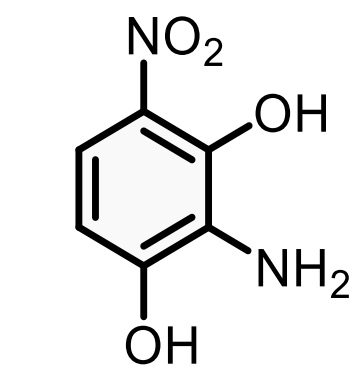	3.73	1.60	0.53	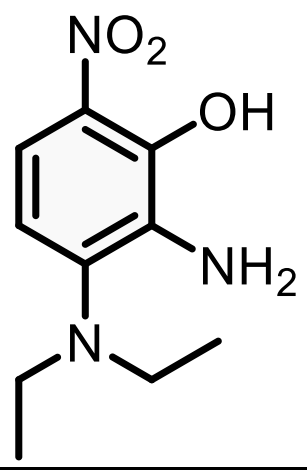	3.51	0.88	1.75
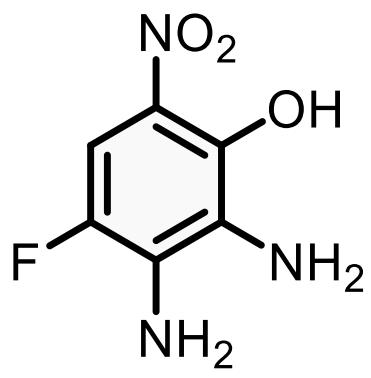	3.03	1.56	-0.09	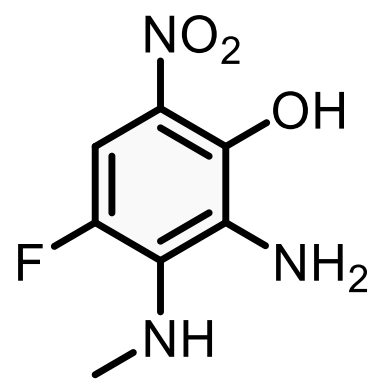	2.78	1.46	-0.14
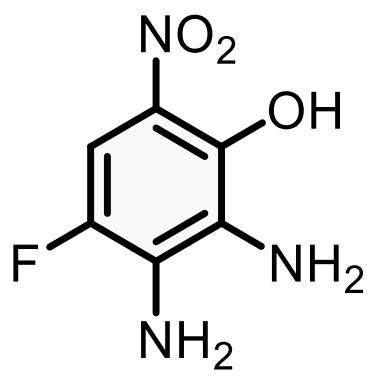	3.03	1.56	-0.09	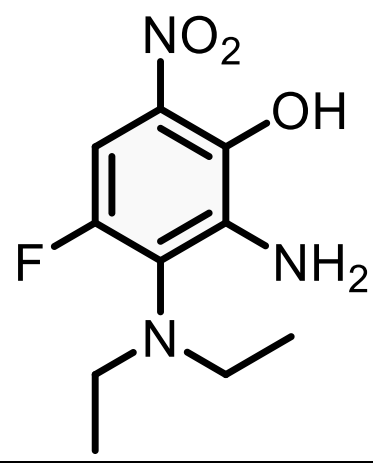	2.62	1.37	-0.12
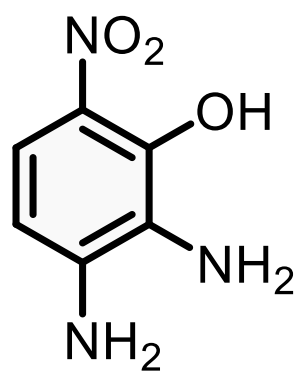	3.08	1.61	-0.14	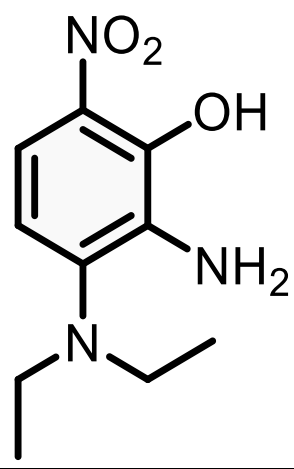	3.51	0.88	1.75
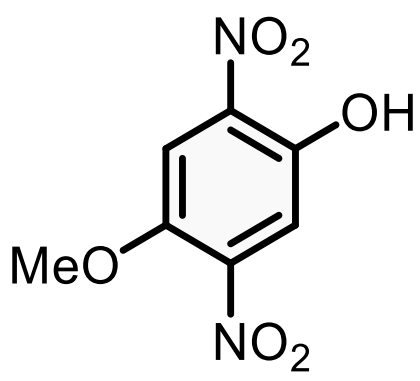	3.14	1.57	0.00	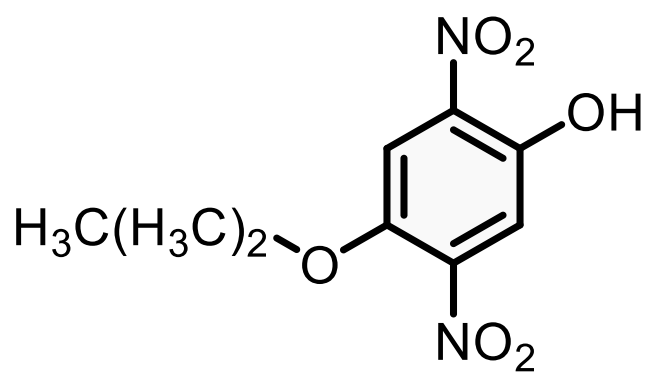	3.10	1.54	0.02
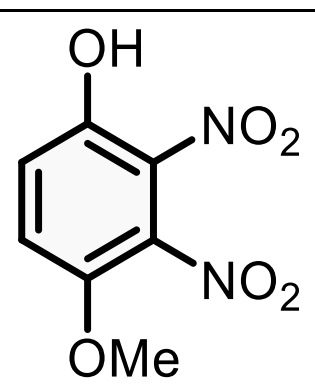	3.29	1.62	0.05	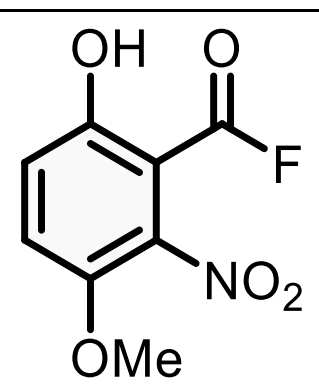	3.30	1.58	0.14

6.2. Anthracene: One-dimensional PSO

Table S14. TD-DFT values of anthracene candidates obtained via the GA/PSO combination and one-dimensional PSO.

Parent				Son			
**Structure**	**S_1/eV**	**T_1/eV**	**S_1-2T_1/eV**	**Structure**	**S_1/eV**	**T_1/eV**	**S_1-2T_1/eV**
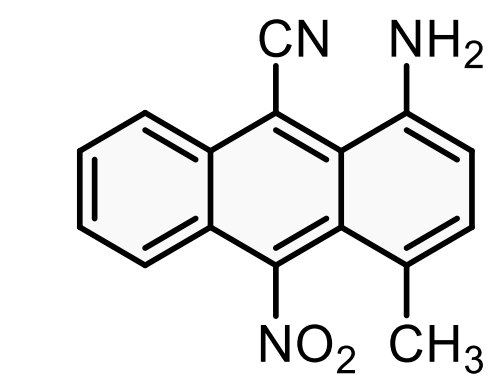	2.23	1.16	-0.07	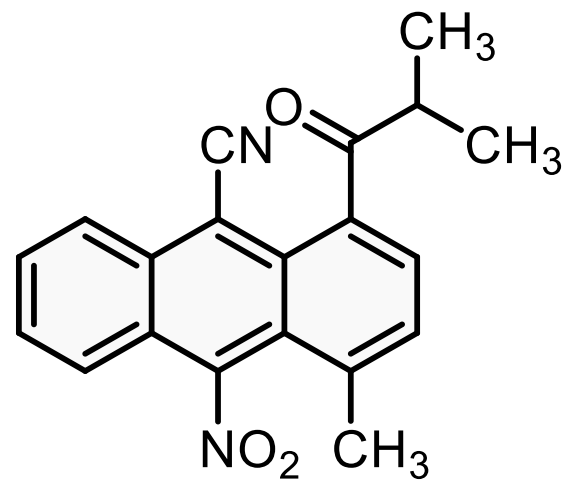	2.74	1.42	-0.10
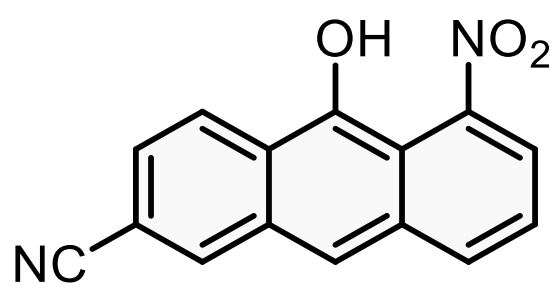	2.21	0.97	0.27	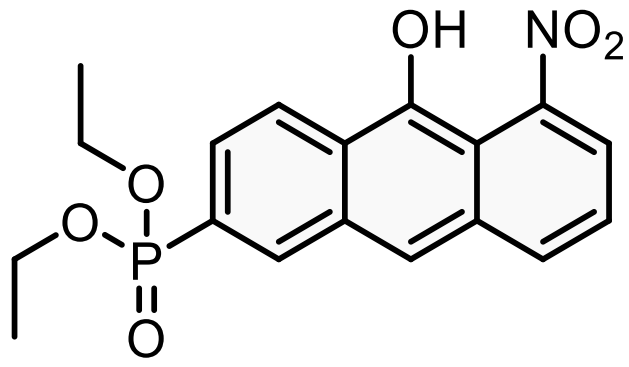	2.16	0.93	0.30
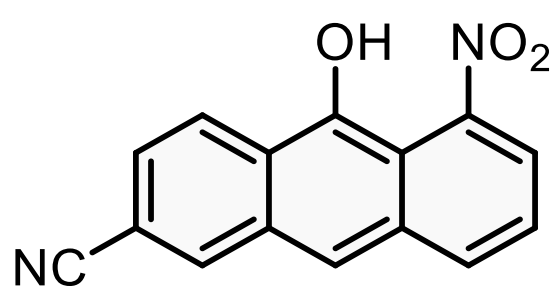	2.21	0.97	0.27	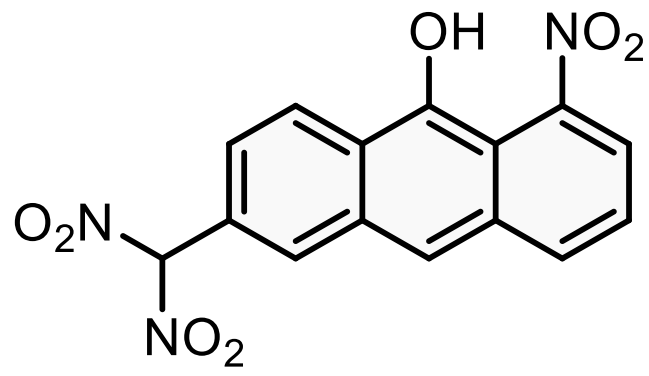	2.21	0.96	0.29
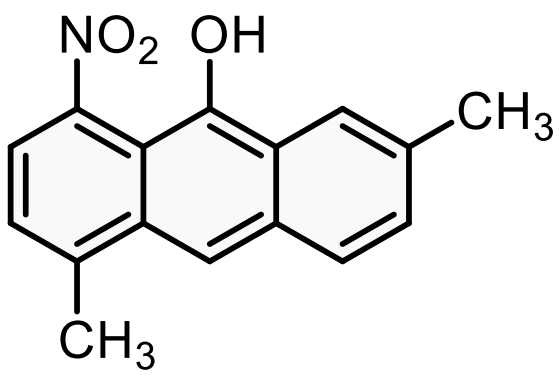	2.13	0.92	0.29	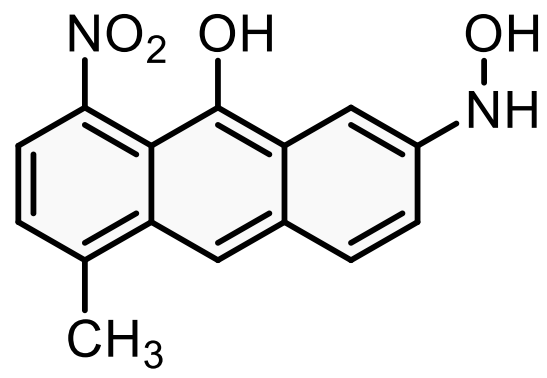	2.03	0.87	0.29
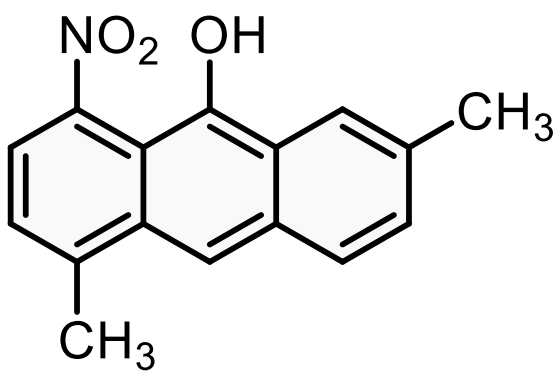	2.13	0.92	0.29	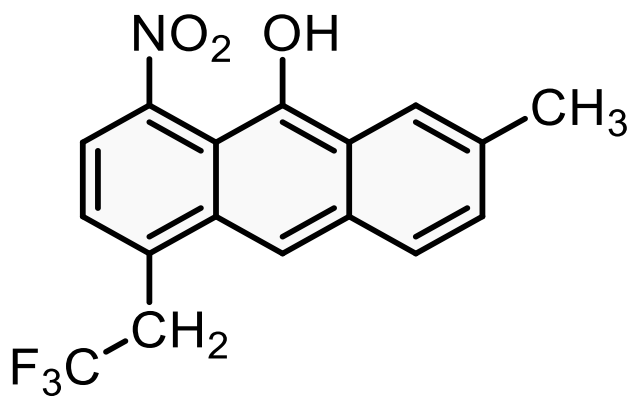	2.11	0.92	0.27
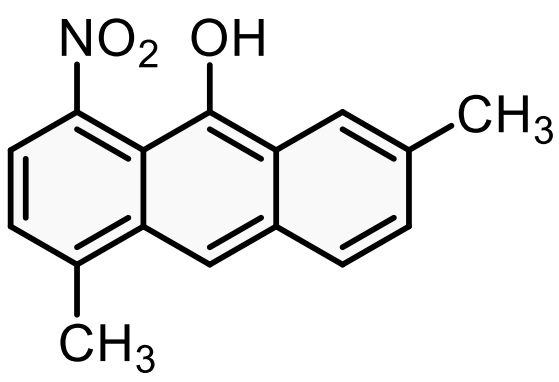	2.13	0.92	0.29	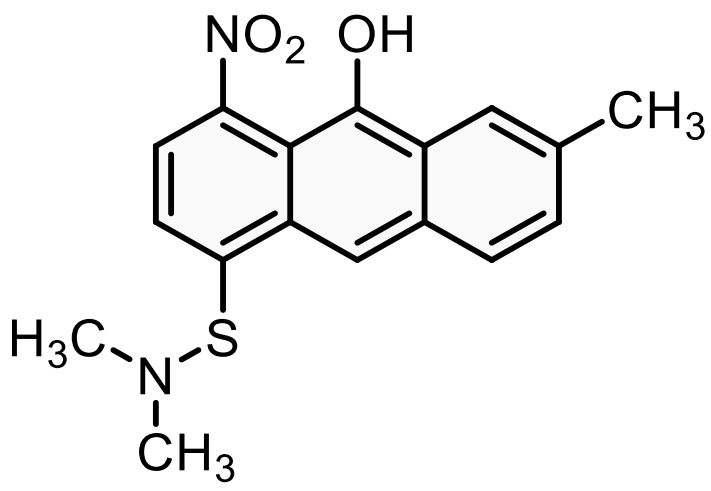	2.16	0.89	0.38
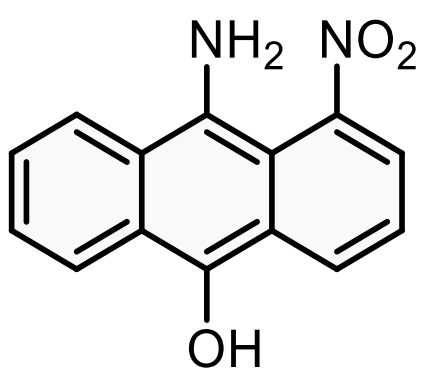	2.01	0.98	0.05	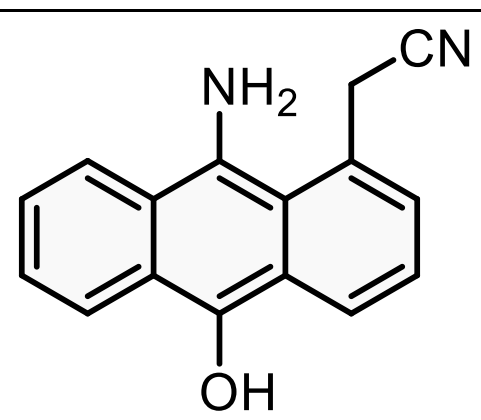	2.87	1.40	0.07

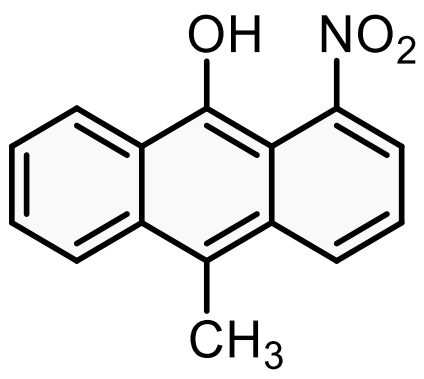	2.05	0.83	0.39	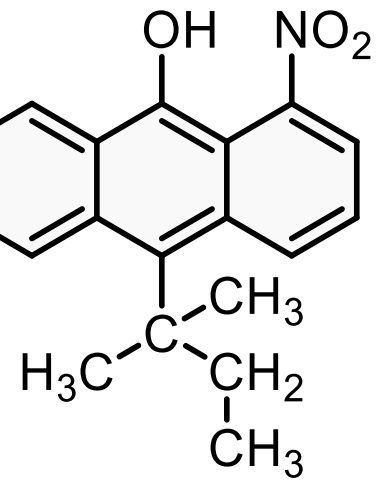	1.99	0.71	0.57
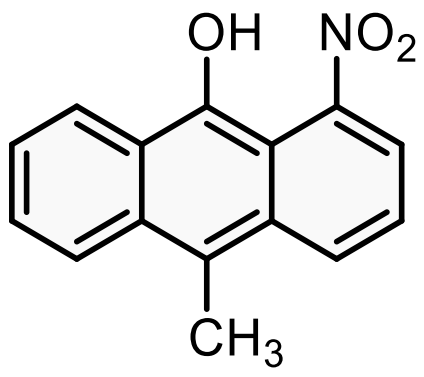	2.05	0.83	0.39	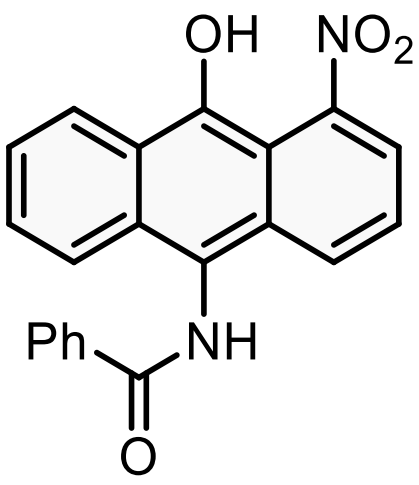	2.10	0.86	0.38
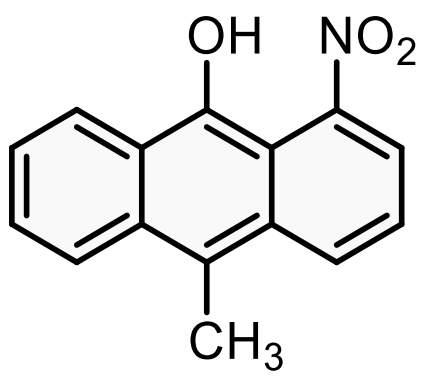	2.05	0.83	0.39	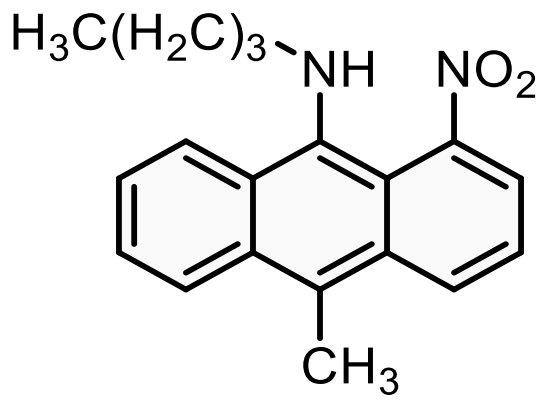	2.90	1.12	0.66
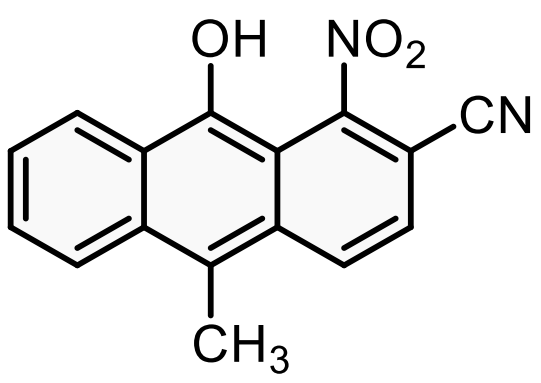	1.97	0.78	0.41	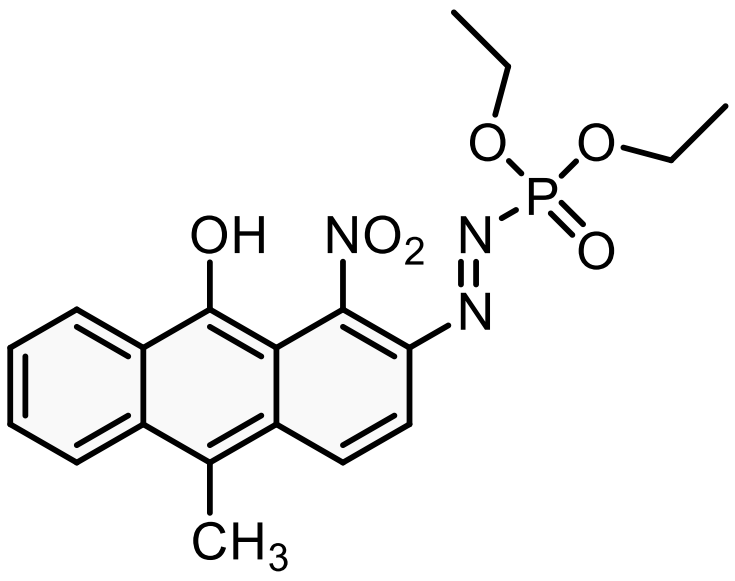	1.93	0.83	0.27
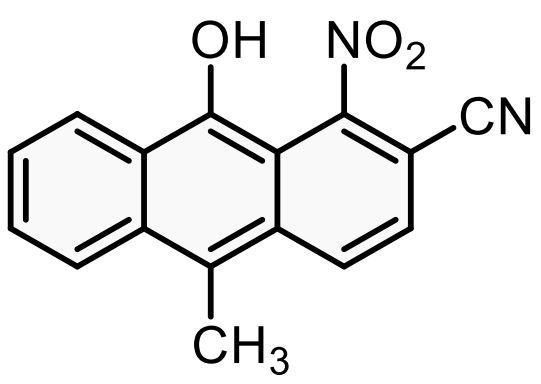	1.97	0.78	0.41	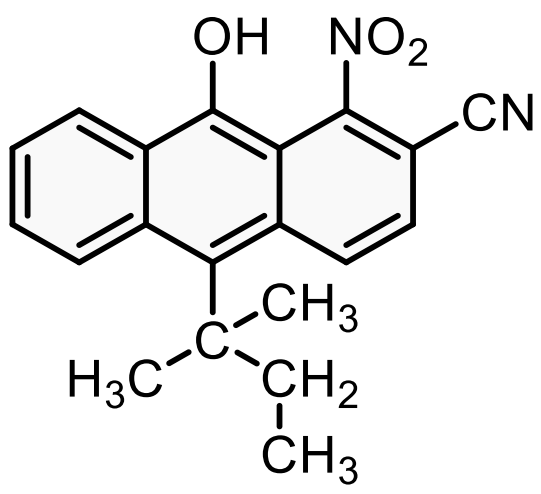	1.95	0.64	0.67
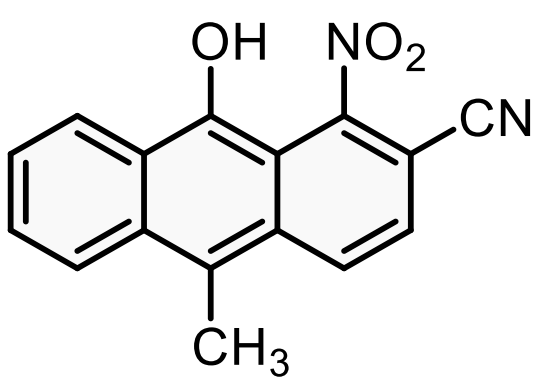	1.97	0.78	0.41	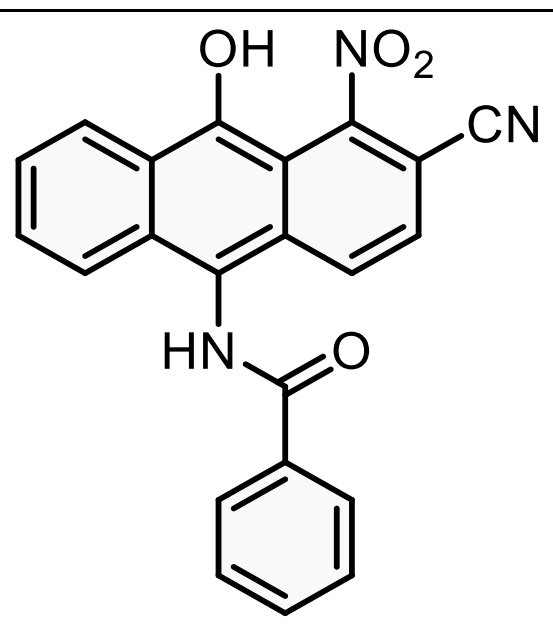	2.01	0.78	0.45
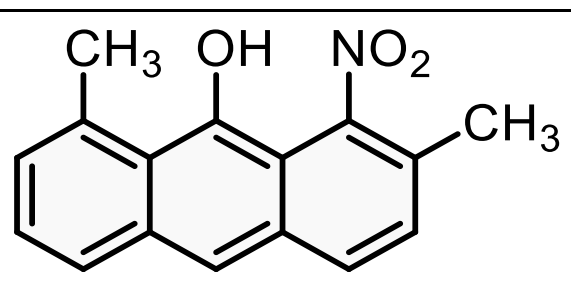	2.14	0.82	0.50	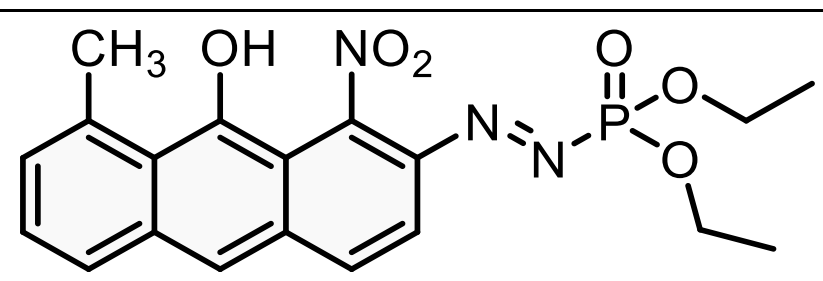	2.01	0.85	0.31

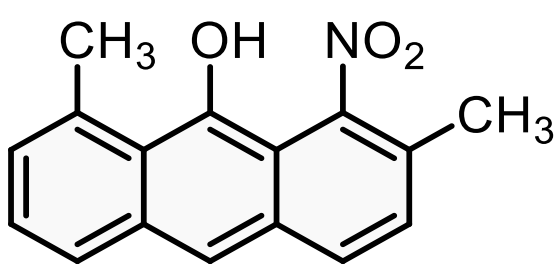	2.14	0.82	0.50	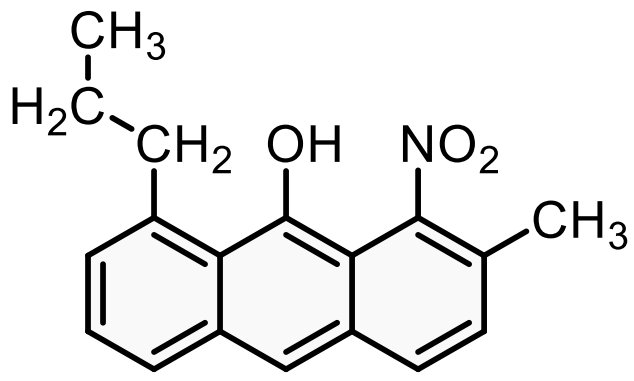	2.14	0.81	0.52

6.3. Anthracenes: Two-dimensional PSO

Table S15. TD-DFT values of anthracene candidates obtained via the GA/PSO combination and two-dimensional PSO.

Parent				Son			
**Structure**	**S_1/eV**	**T_1/eV**	**S_1-$2T_1$/eV**	**Structure**	**S_1/eV**	**T_1/eV**	**S_1-$2T_1$/eV**
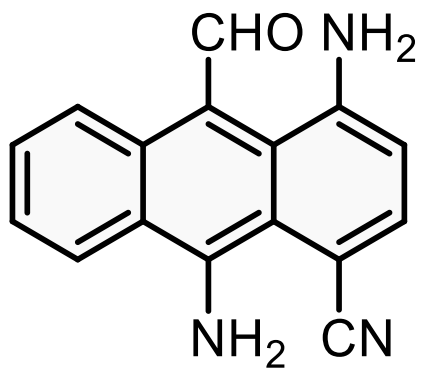	2.58	1.30	-0.02	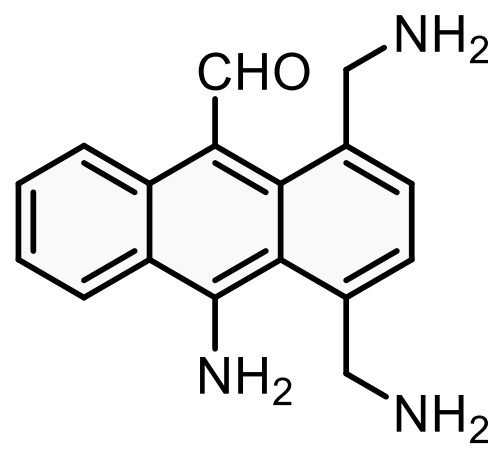	2.71	1.32	-0.07
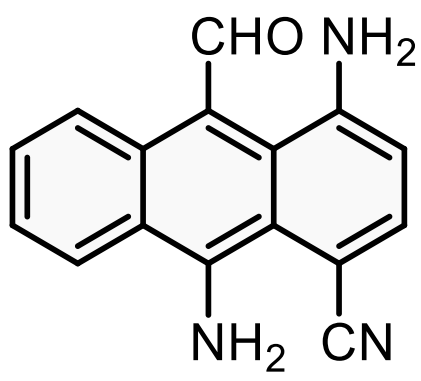	2.58	1.30	-0.02	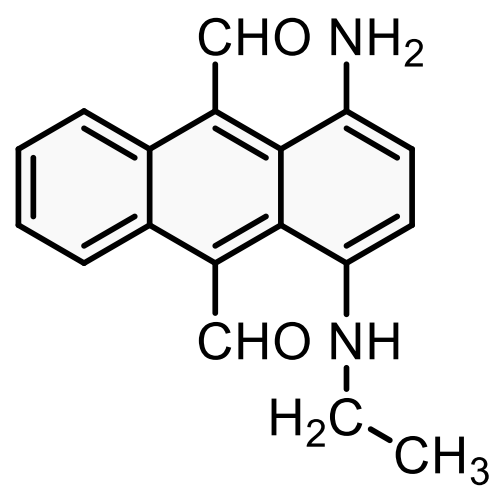	2.40	1.01	0.38
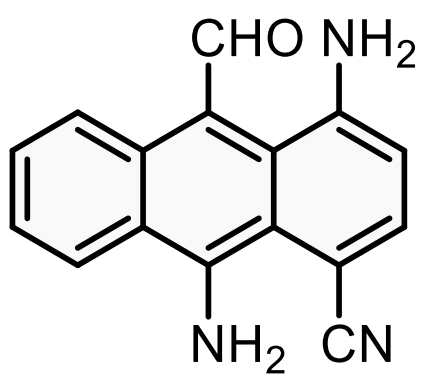	2.58	1.30	-0.02	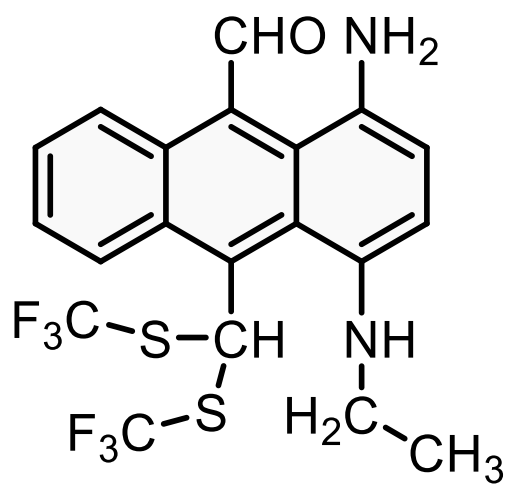	2.68	0.88	0.92
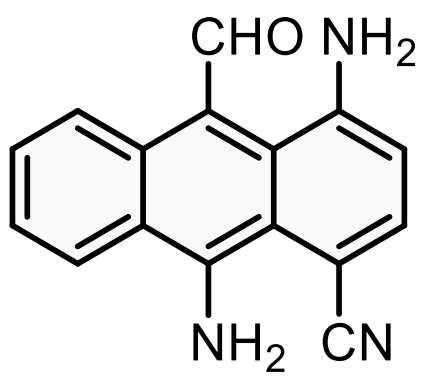	2.58	1.30	-0.02	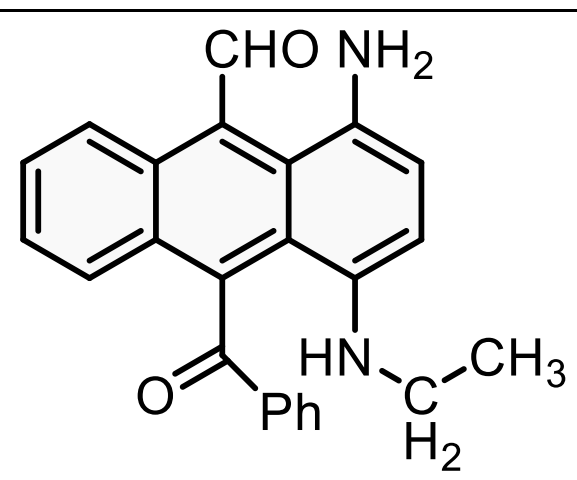	2.27	1.02	0.23
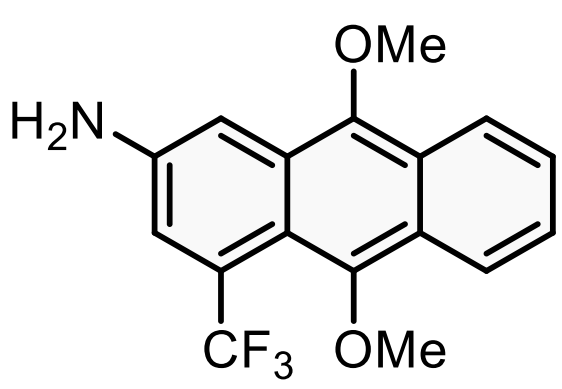	2.87	1.59	-0.31	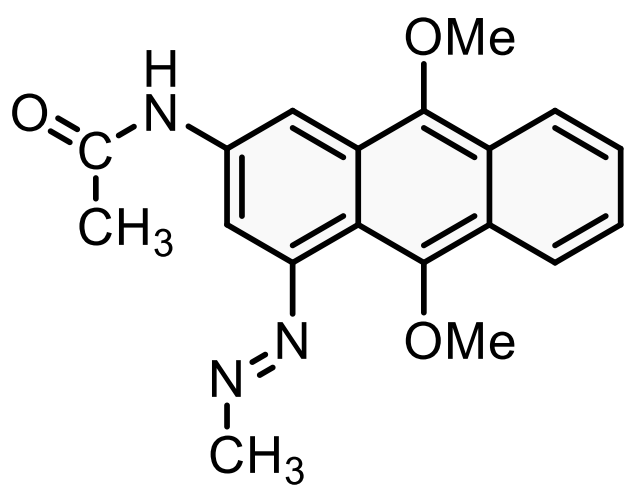	2.36	1.13	0.10

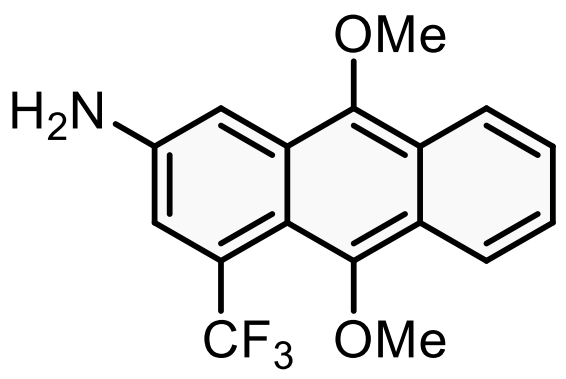	2.87	1.59	-0.31	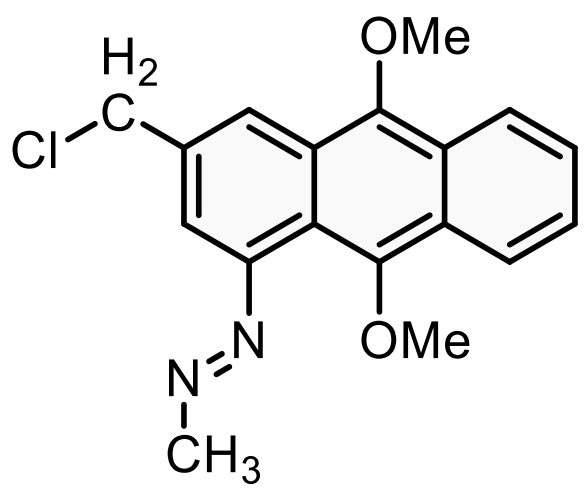	2.46	1.18	0.10
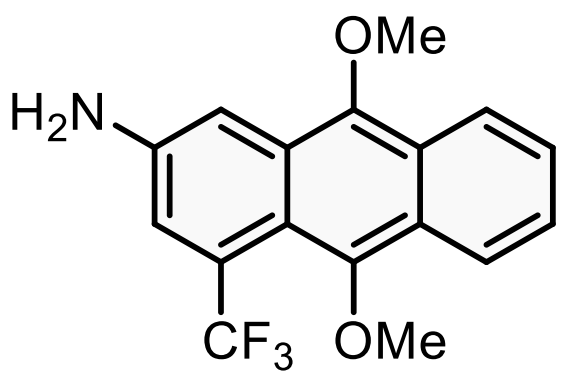	2.87	1.59	-0.31	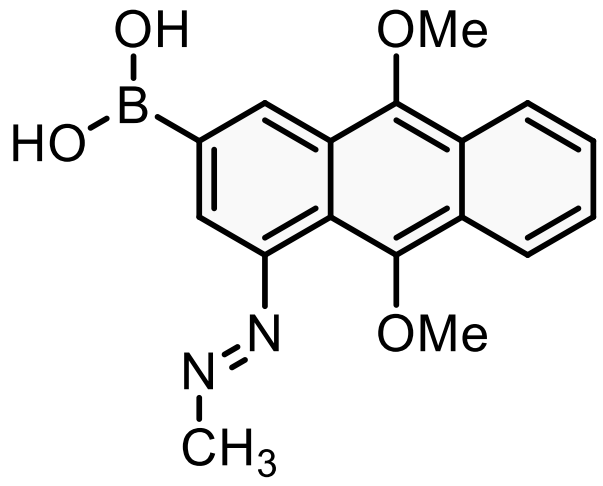	2.43	1.18	0.07
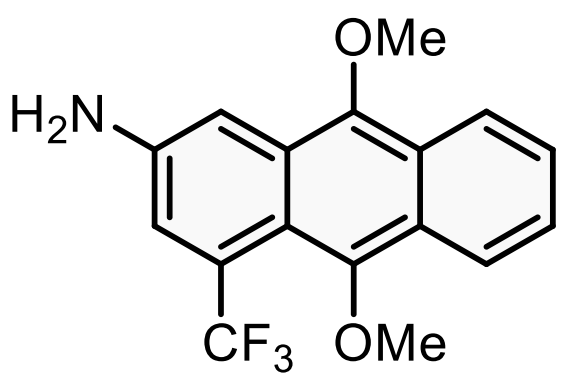	2.87	1.59	-0.31	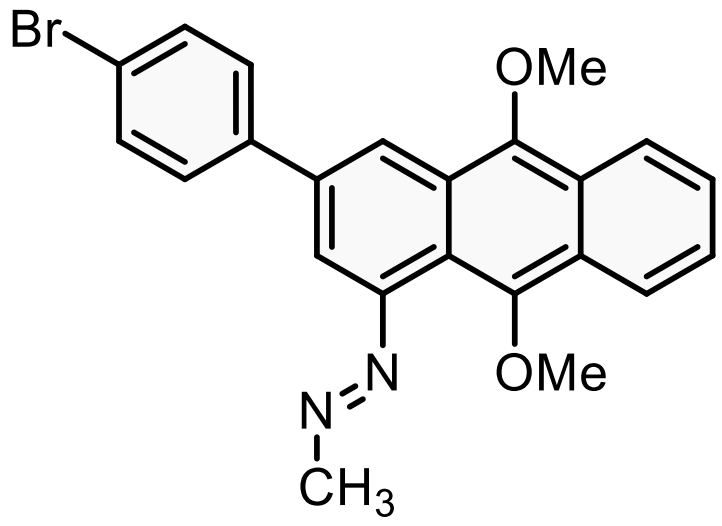	2.39	1.17	0.05
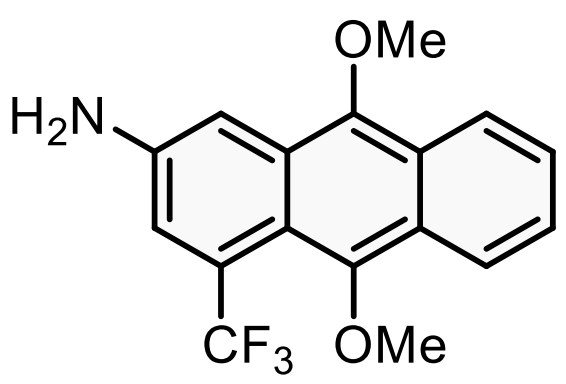	2.87	1.59	-0.31	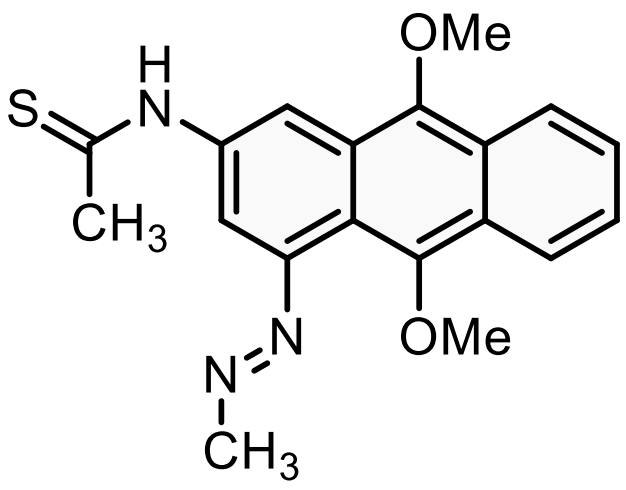	2.30	1.11	0.08
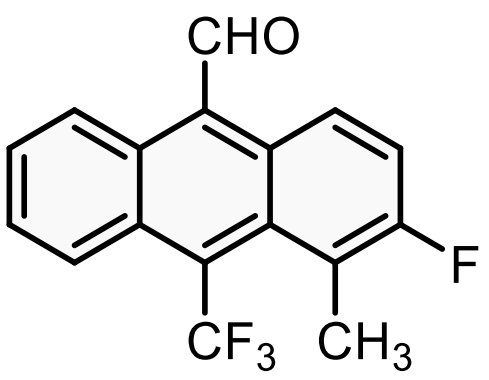	2.68	1.27	0.14	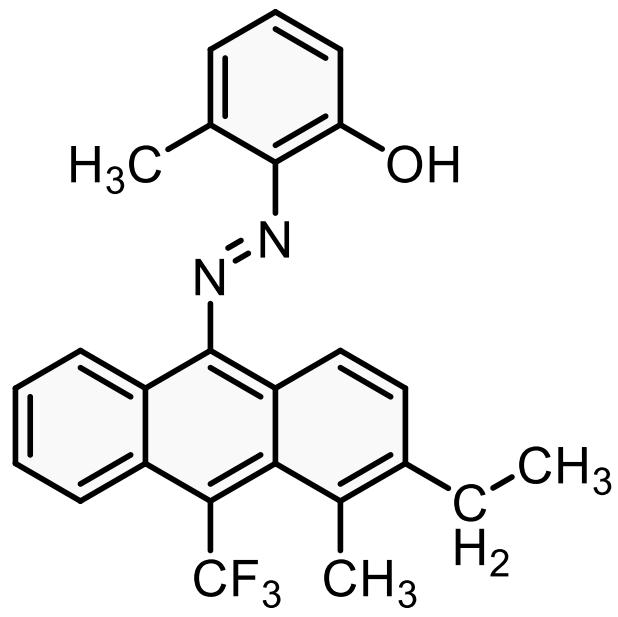	2.08	0.70	0.68
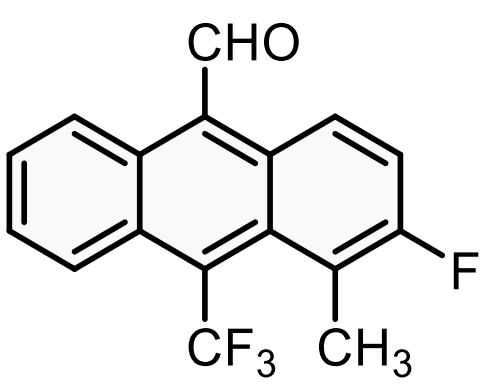	2.68	1.27	0.14	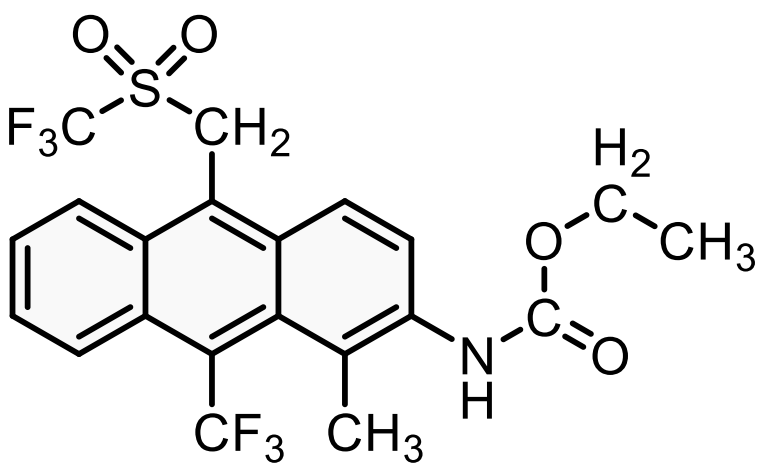	2.77	1.43	-0.09
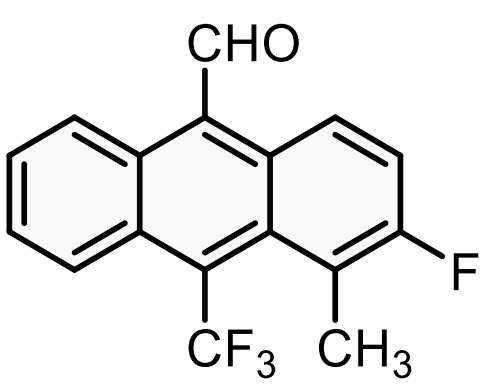	2.68	1.27	0.14	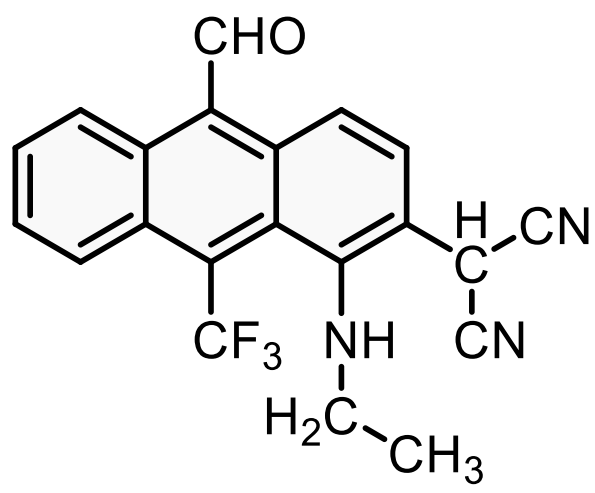	2.64	1.27	0.10

6.4. Anthracenes: Three-dimensional PSO

Table S16. TD-DFT values of anthracene candidates obtained via the GA/PSO combination and three-dimensional PSO.

Parent				Son			
**Structure**	**S_1/eV**	**T_1/eV**	**S_1-$2T_1$/eV**	**Structure**	**S_1/eV**	**T_1/eV**	**S_1-$2T_1$/eV**
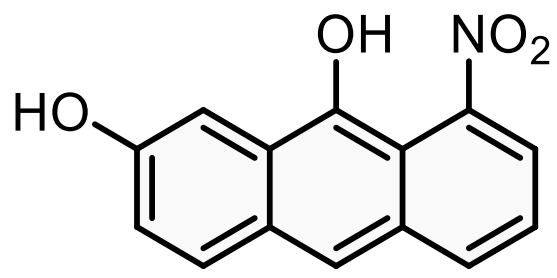	2.07	0.87	0.33	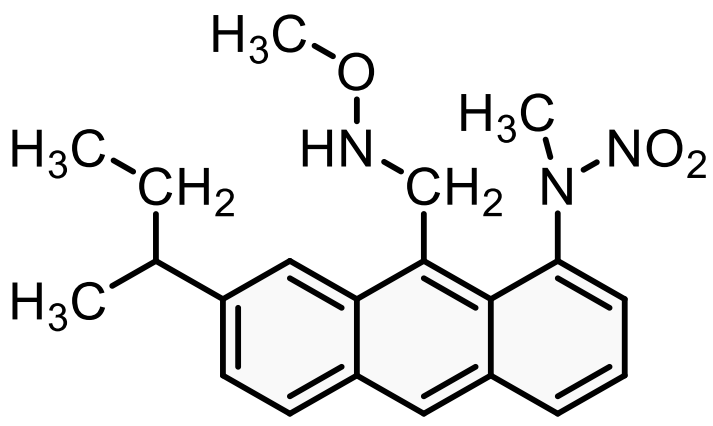	3.03	1.32	0.39
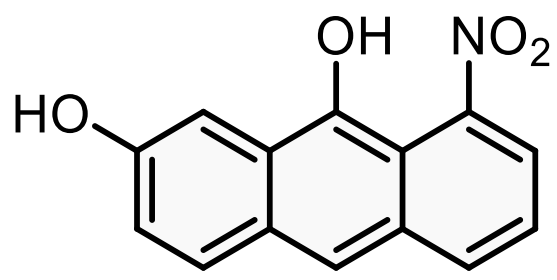	2.07	0.87	0.33	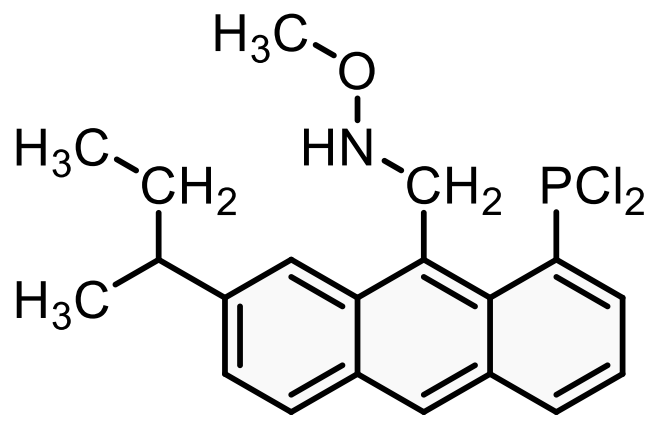	2.65	1.34	-0.03
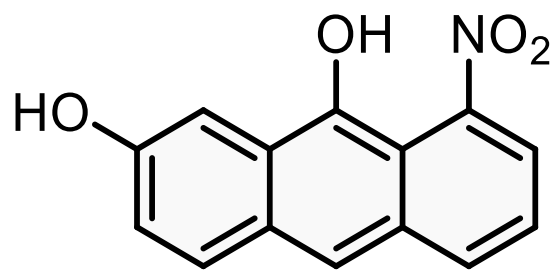	2.07	0.87	0.33	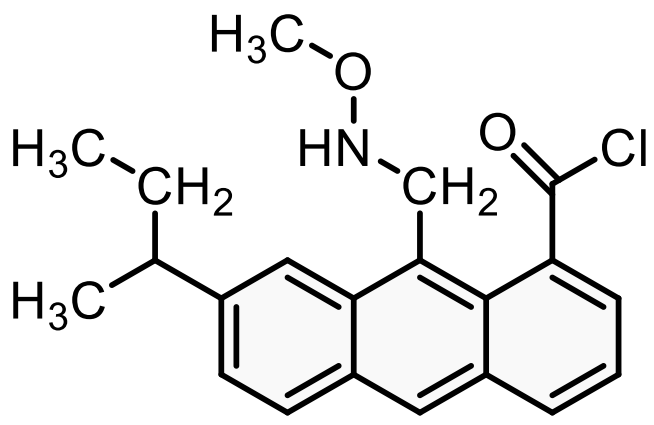	2.89	1.46	-0.03
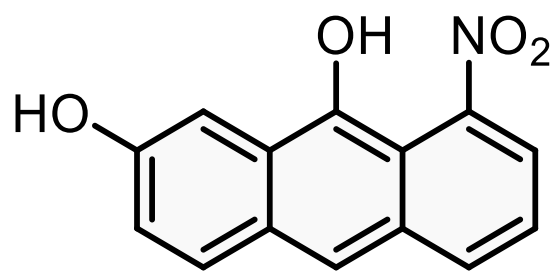	2.07	0.87	0.33	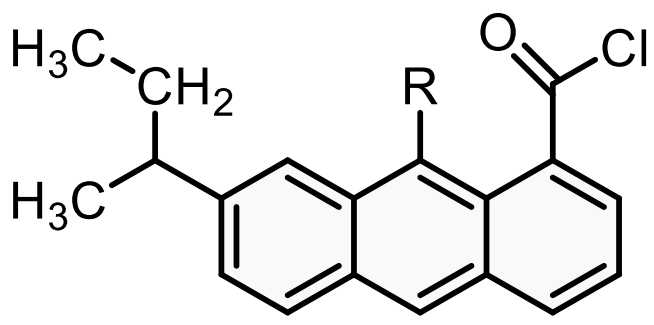 R = $CH(OH)(CF_3)_2$	3.01	1.40	0.21
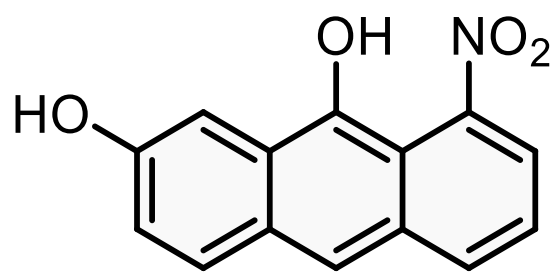	2.07	0.87	0.33	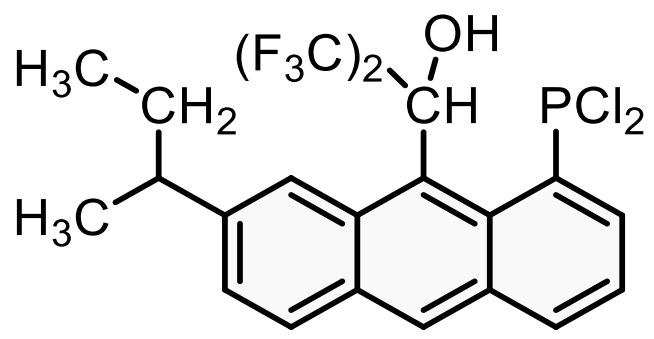	2.94	1.47	0.00
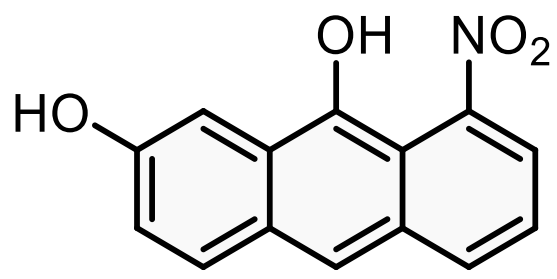	2.07	0.87	0.33	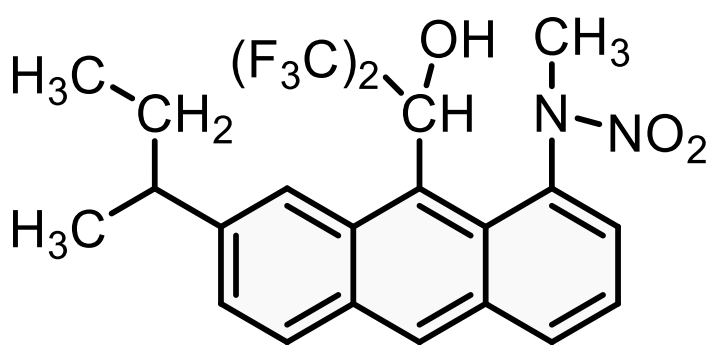	2.93	1.42	0.09
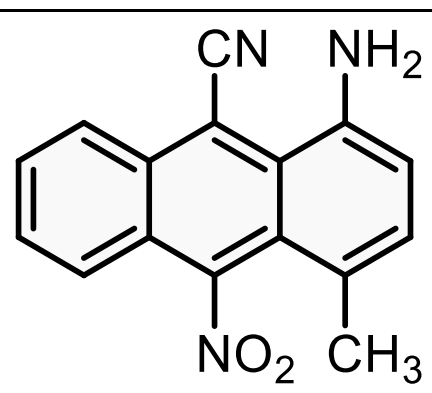	2.23	1.16	-0.09	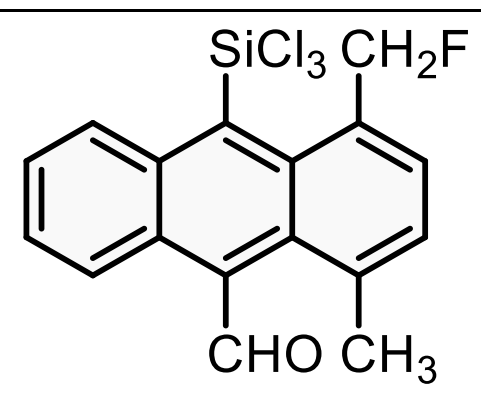	2.52	1.07	0.38
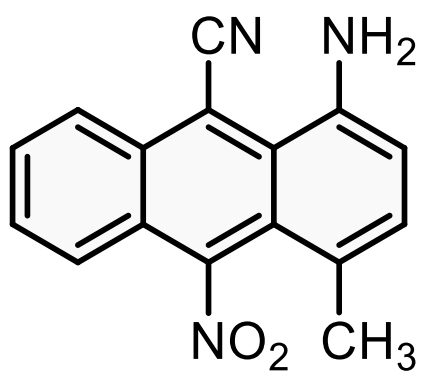	2.23	1.16	-0.09	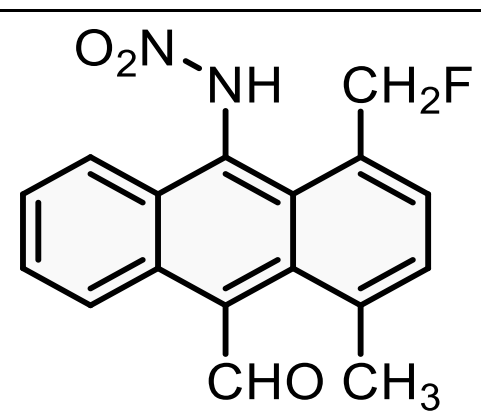	2.81	1.33	0.15

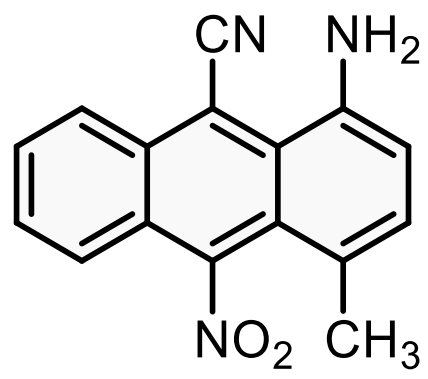	2.23	1.16	-0.09	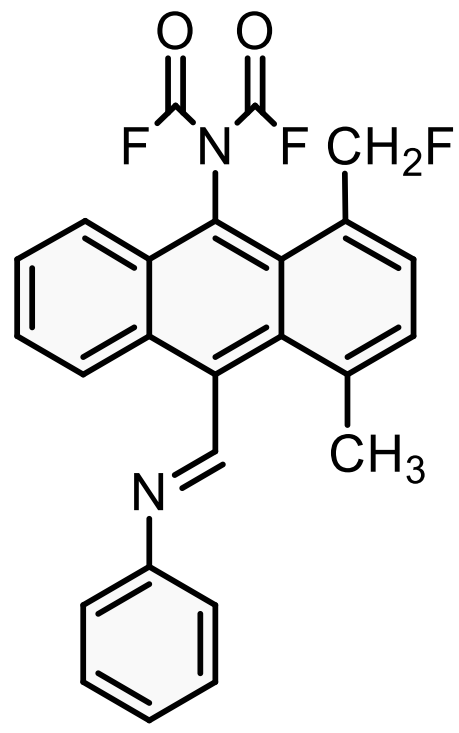	2.66	1.25	0.16
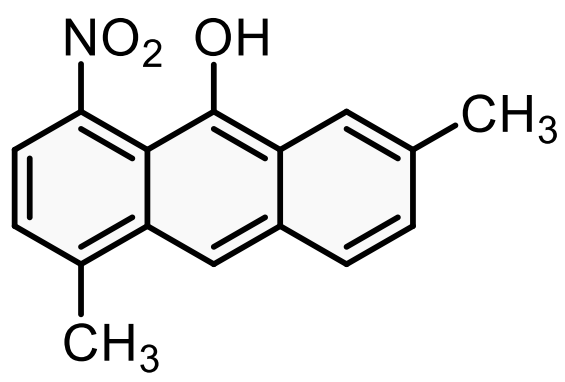	2.13	0.92	0.29	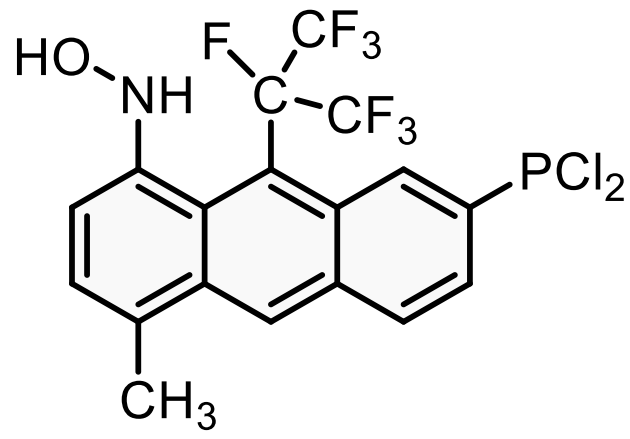	2.92	1.18	0.56
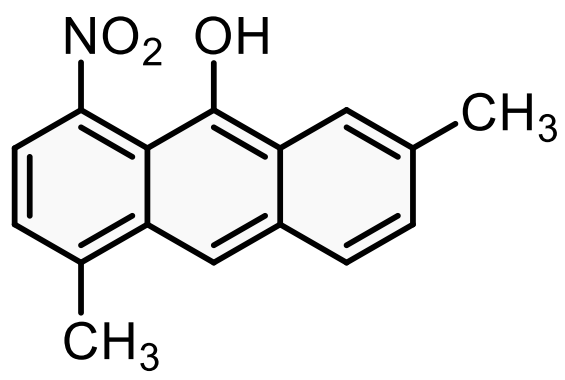	2.13	0.92	0.29	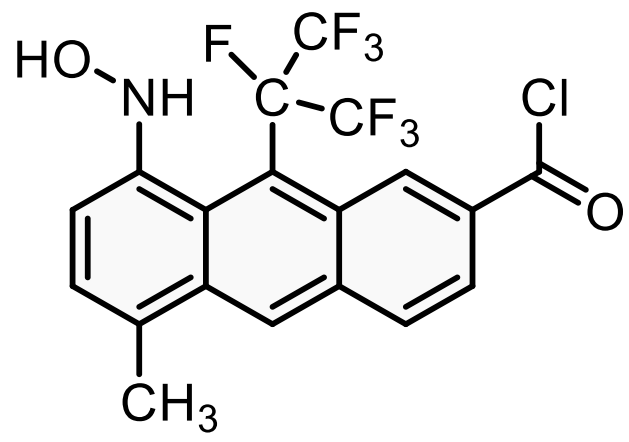	2.76	1.10	0.56
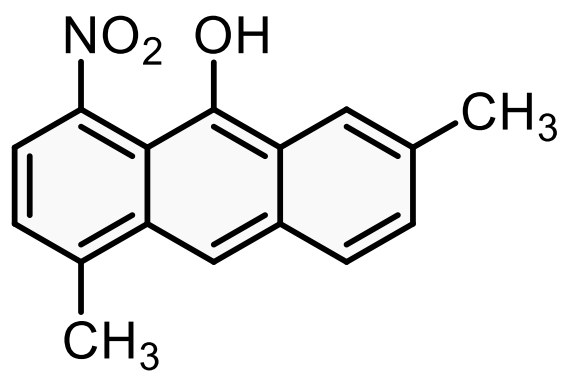	2.13	0.92	0.29	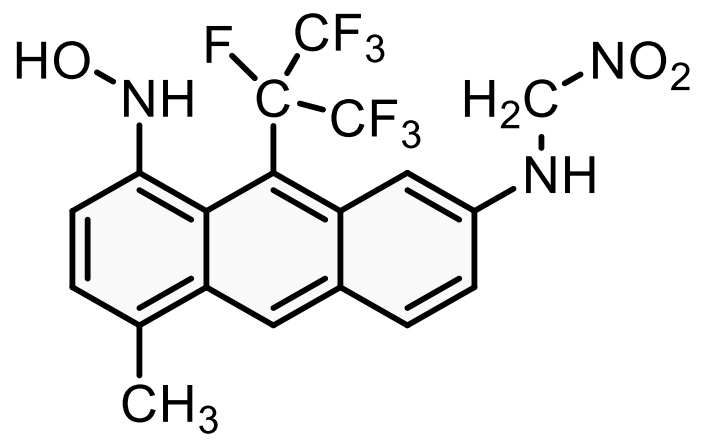	2.92	1.25	0.42
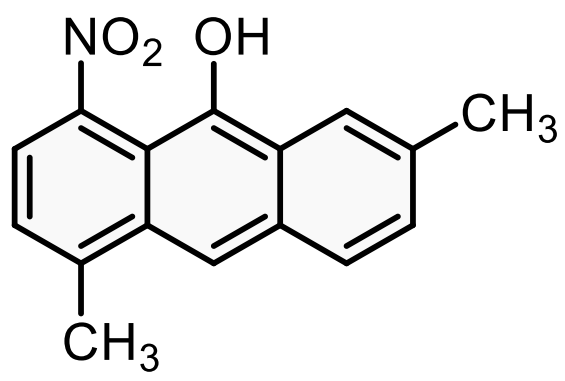	2.13	0.92	0.29	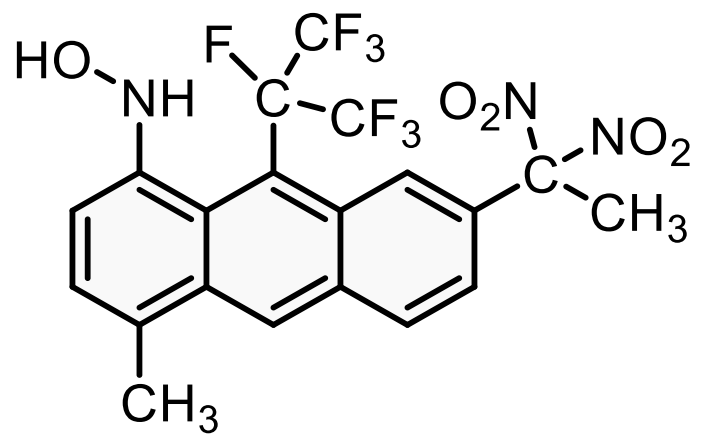	2.77	1.10	0.57

6.5. Anthracenes: Four-dimensional PSO

Table S17. TD-DFT values of anthracene candidates obtained via the GA/PSO combination and four-dimensional PSO.

Parent				Son			
**Structure**	**S_1/eV**	**T_1/eV**	**S_1-$2T_1$/eV**	**Structure**	**S_1/eV**	**T_1/eV**	**S_1-$2T_1$/eV**
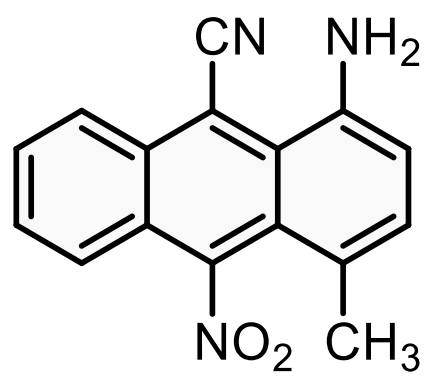	2.23	1.16	-0.09	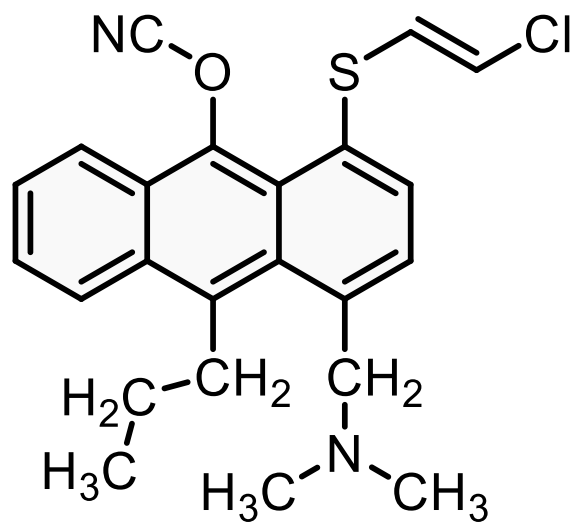	2.13	0.92	0.29
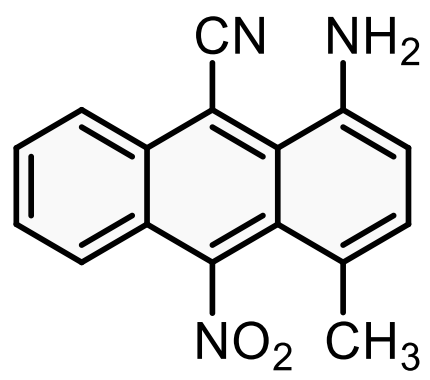	2.23	1.16	-0.09	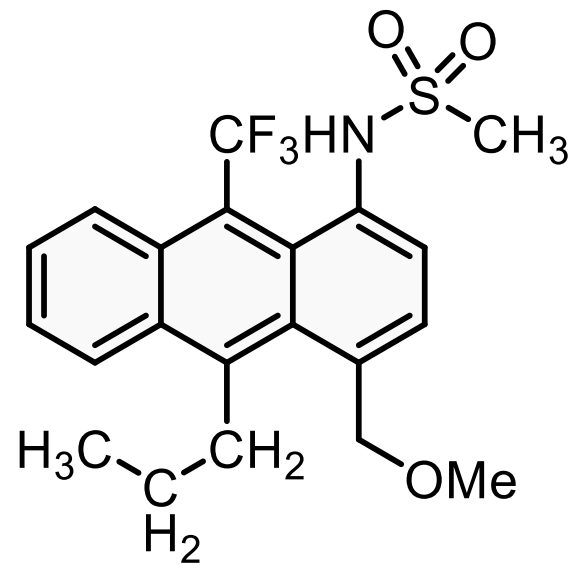	2.86	1.42	0.02
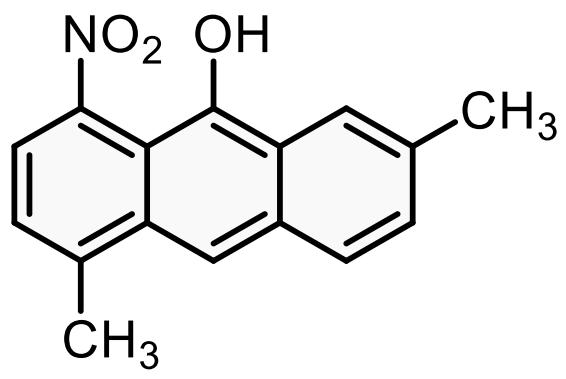	2.13	0.92	0.29	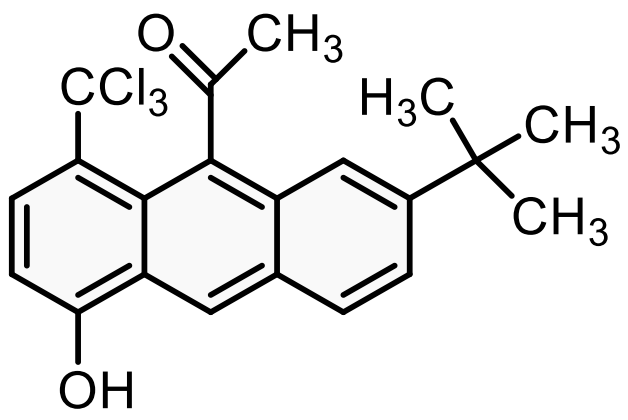	2.90	1.40	0.10
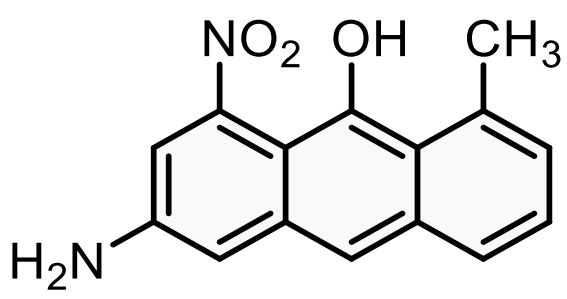	1.88	0.72	0.44	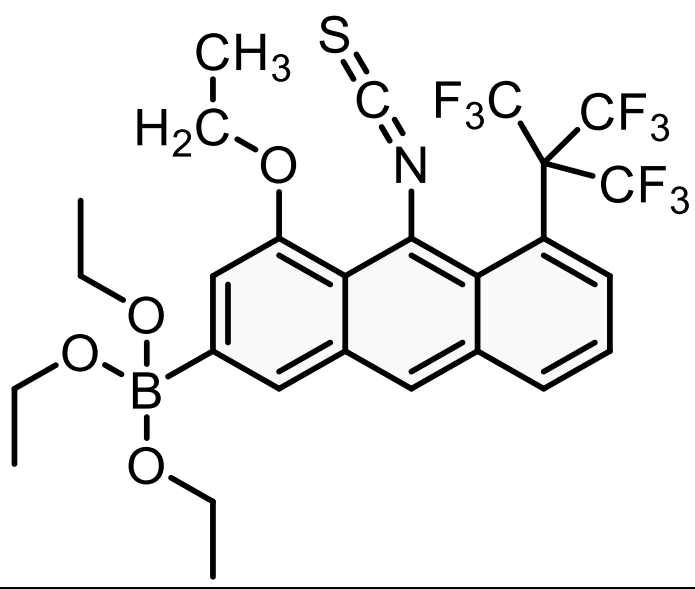	2.63	1.28	0.07
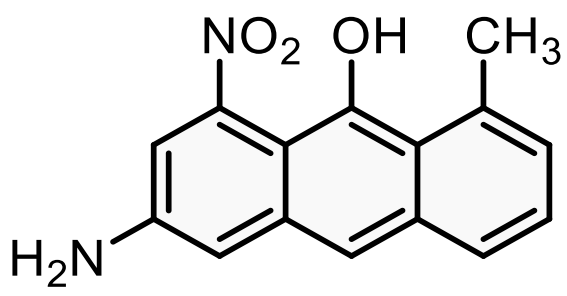	1.88	0.72	0.44	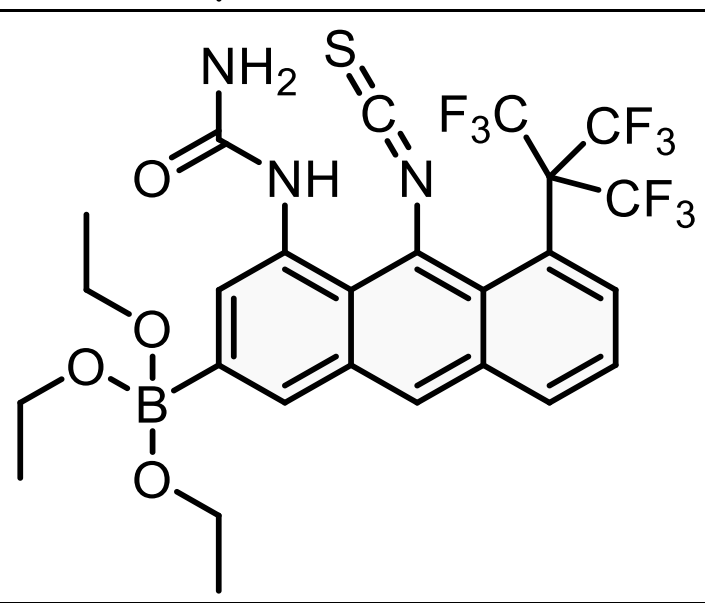	2.73	1.34	0.05

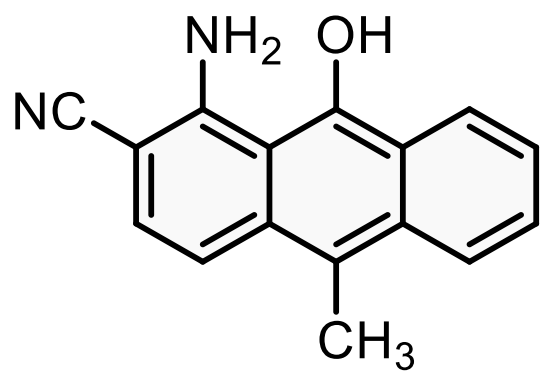	1.97	0.78	0.41	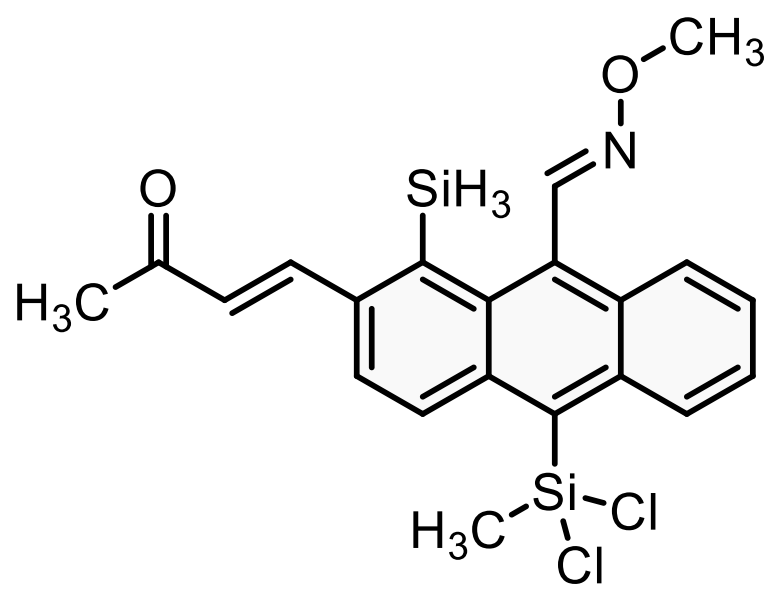	2.48	1.04	0.40
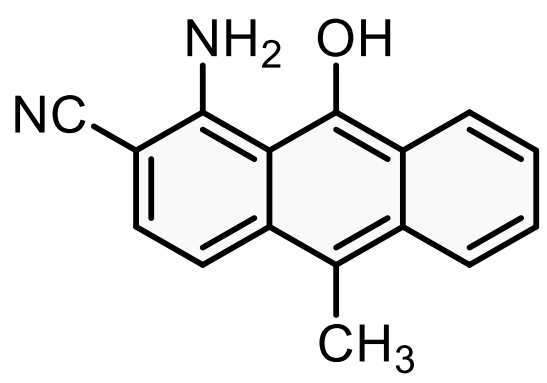	1.97	0.78	0.41	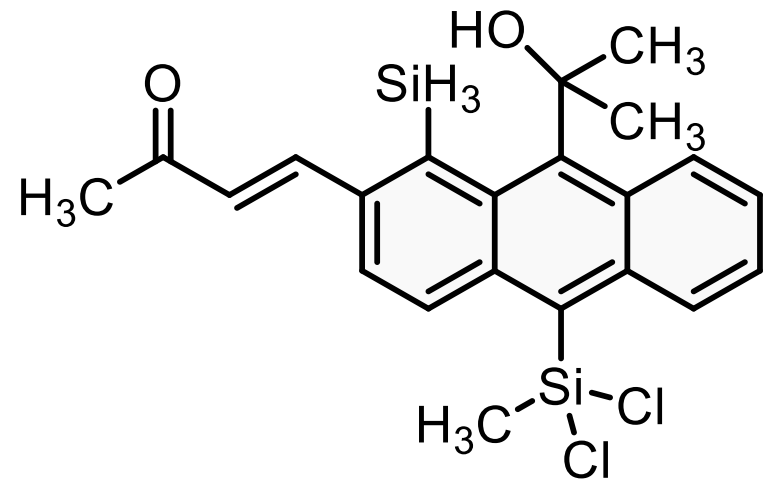	2.70	1.22	0.26
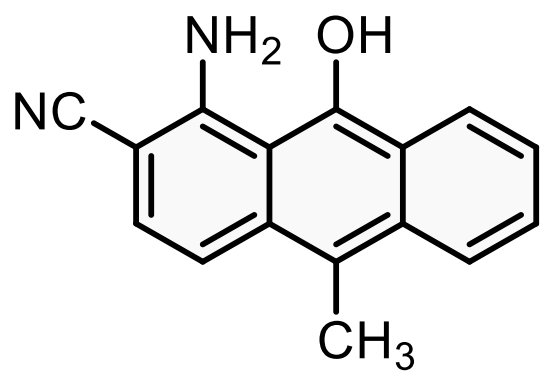	1.97	0.78	0.41	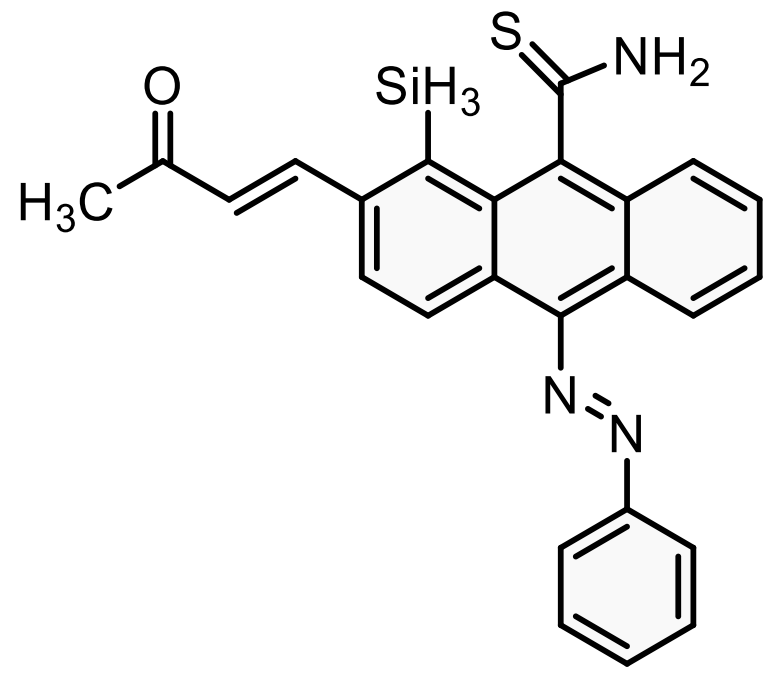	2.18	0.87	0.44
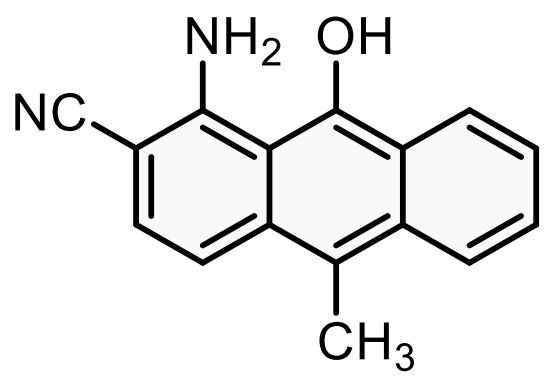	1.97	0.78	0.41	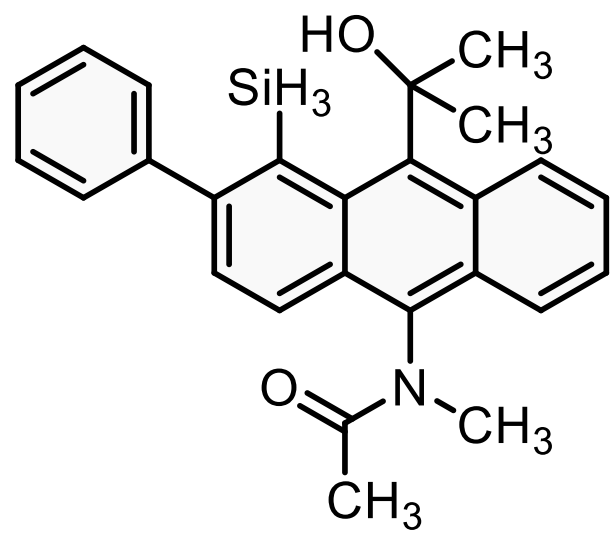	2.94	1.43	0.08
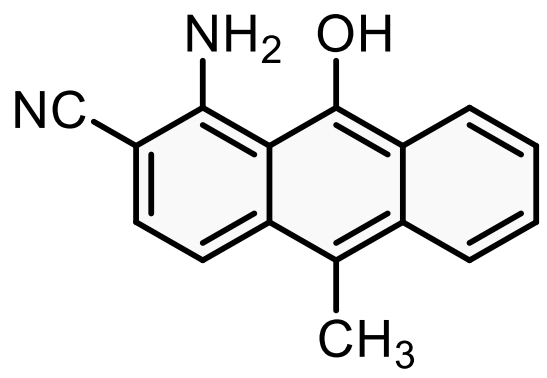	1.97	0.78	0.41	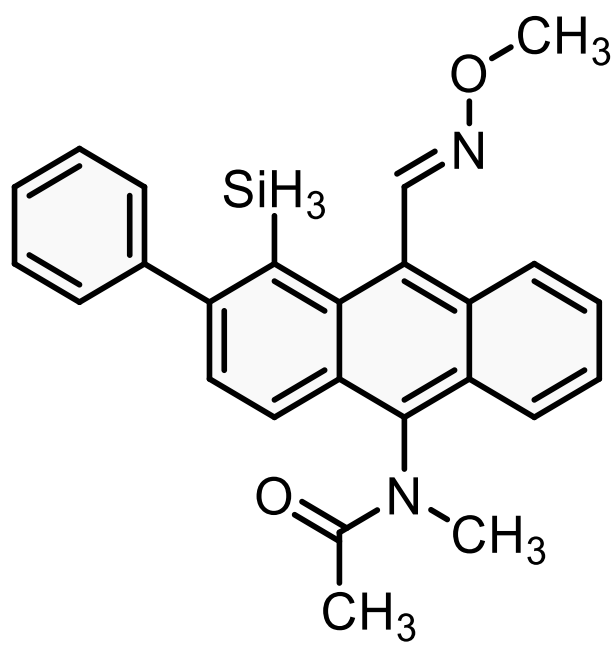	2.75	1.25	0.25

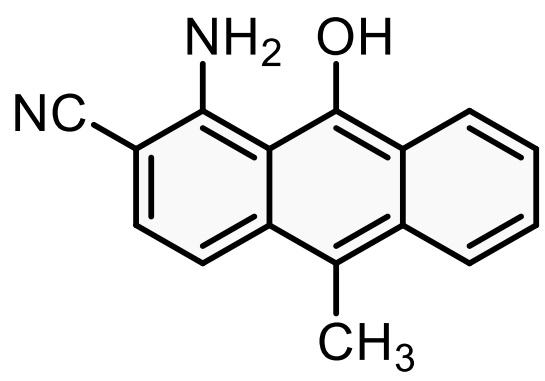	1.97	0.78	0.41	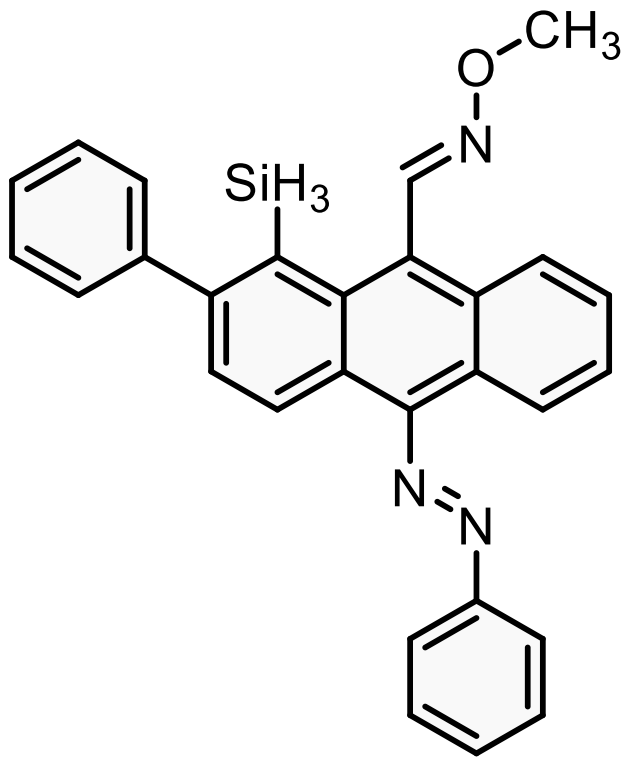	2.17	0.85	0.47
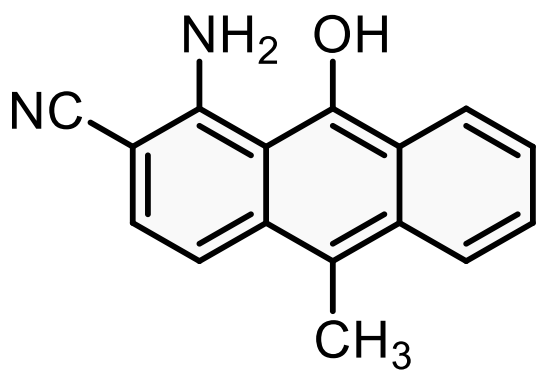	1.97	0.78	0.41	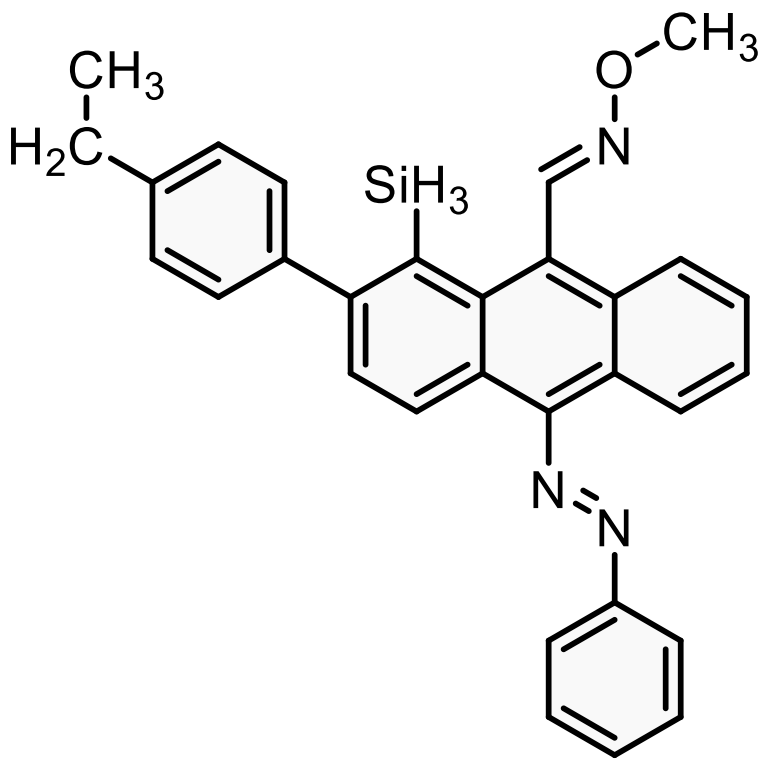	2.17	0.85	0.47
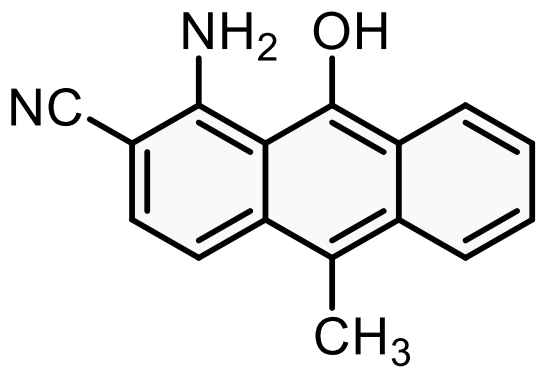	1.97	0.78	0.41	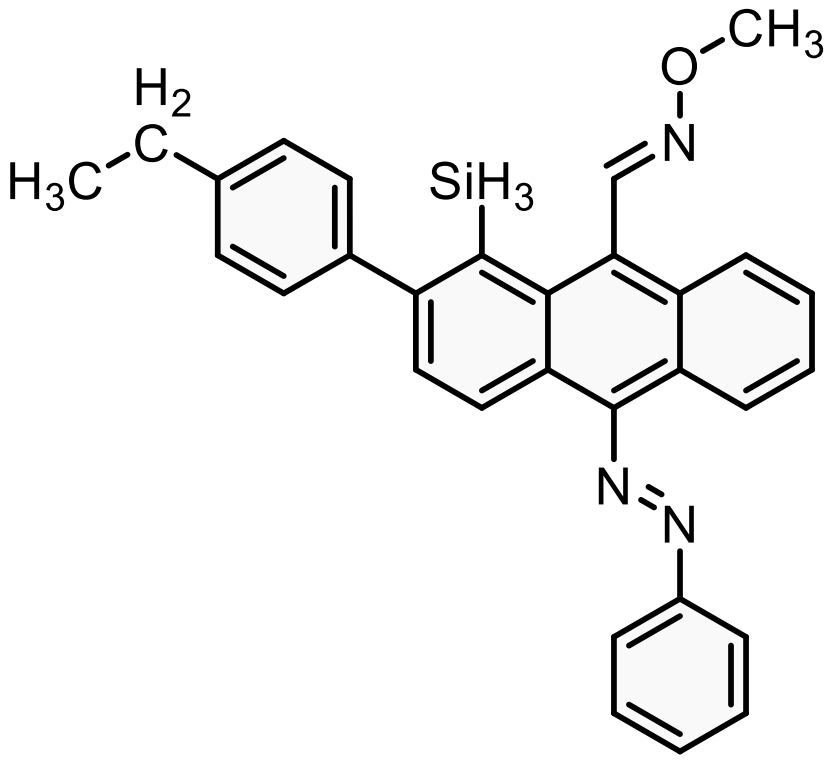	2.17	0.85	0.47
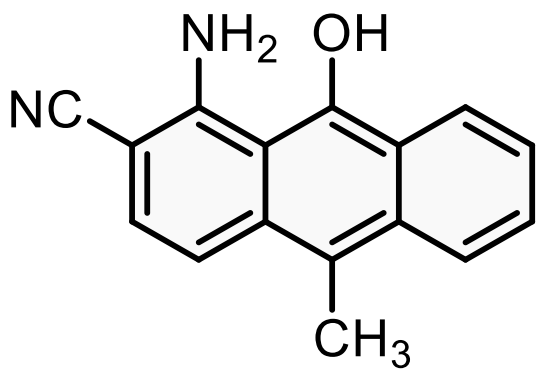	1.97	0.78	0.41	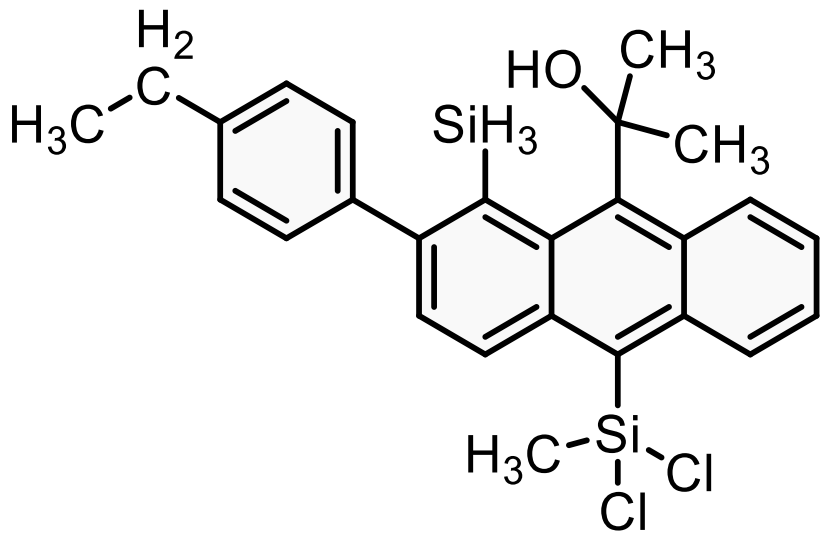	2.84	1.24	0.36
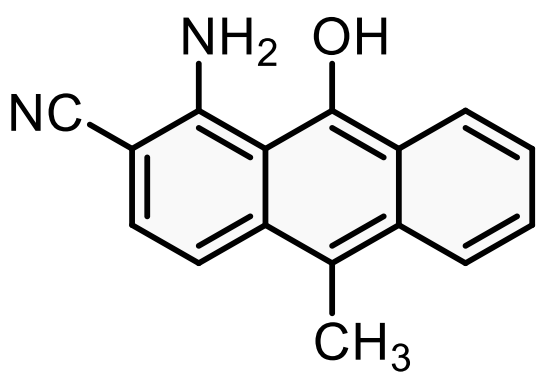	1.97	0.78	0.41	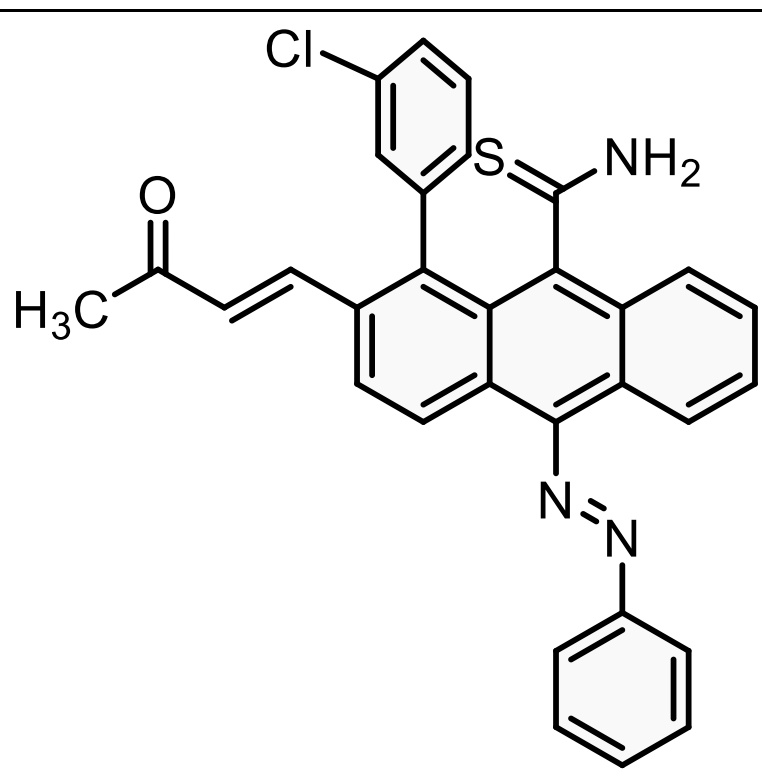	2.16	0.84	0.48

7. Symmetric compounds

Finally, to illustrate how our methodology can facilitate the design of compounds with improved properties, we present a selection of symmetric compounds, which are therefore easier to obtain through synthesis. This aspect further reinforces the idea that our approach not only enables the identification of compounds with exceptional properties, but also those that are synthetically accessible. Given the relevance of symmetry in this context, and although the substituents considered correspond to the original set of ten, the molecular structures are explicitly included.

Table S18. TD-DFT values of candidates obtained through inverse design.

Structure	S_1/eV	T_1/eV	S_1-2T_1/eV
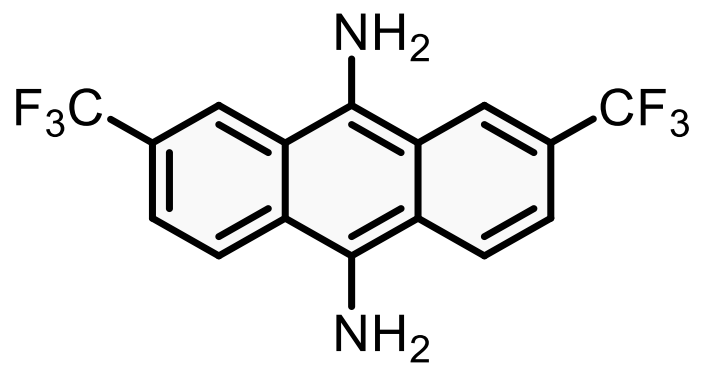	2.47	1.25	-0.02
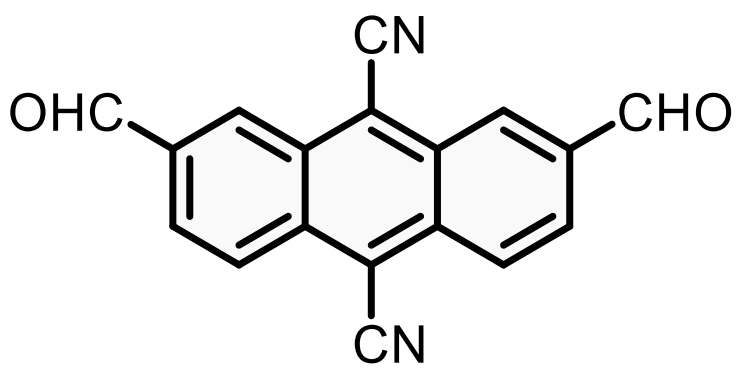	2.64	1.36	-0.08
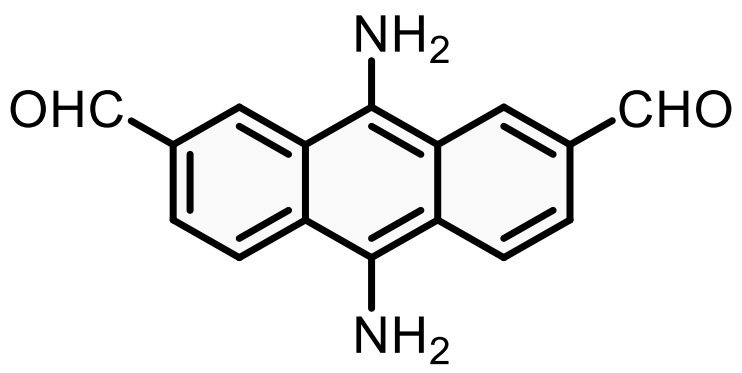	2.07	1.08	-0.09
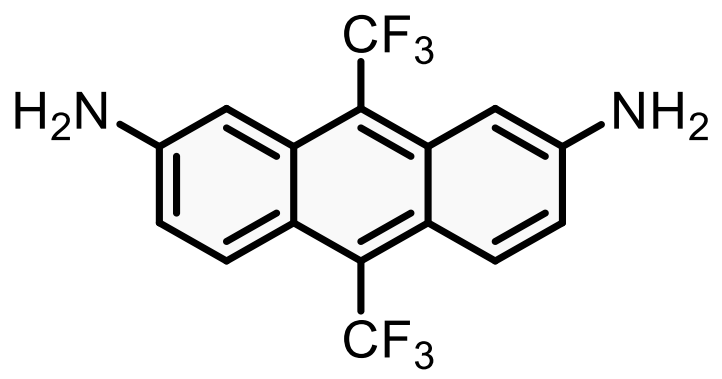	2.93	1.51	-0.09
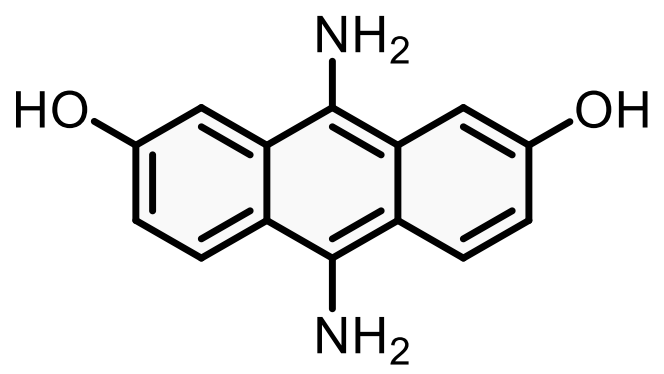	2.45	1.26	-0.07
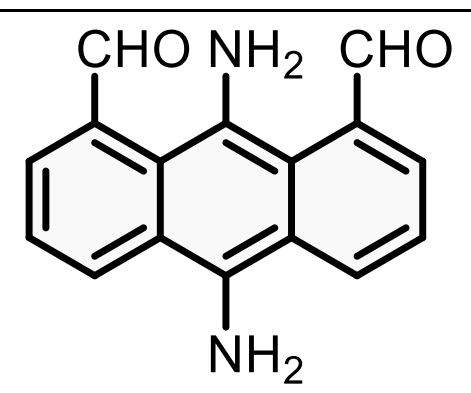	1.64	0.71	0.22
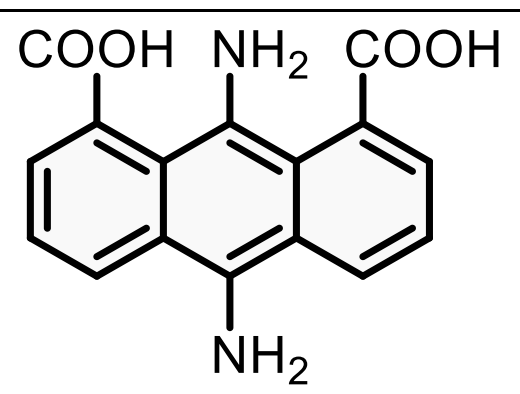	2.24	1.08	0.08

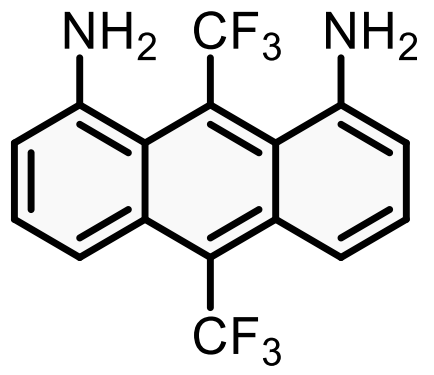	2.08	0.95	0.18
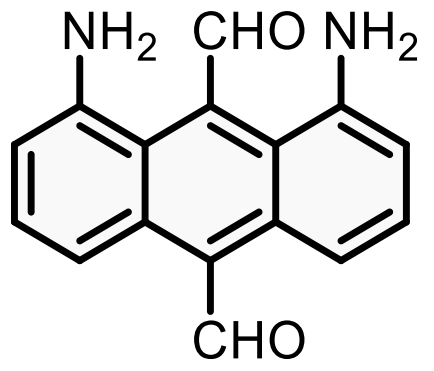	1.71	0.70	0.31
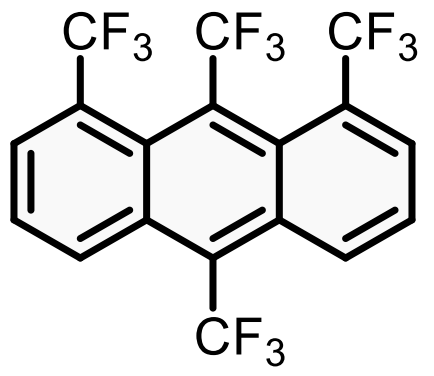	2.89	1.12	0.65
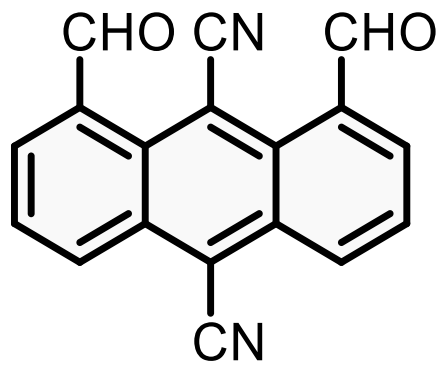	2.68	1.34	-0.01
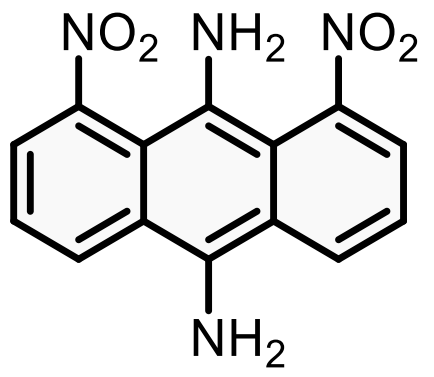	1.80	0.88	0.04
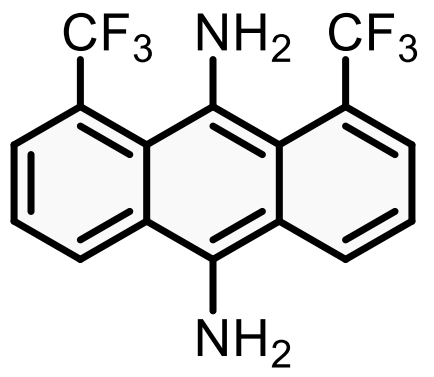	2.13	0.97	0.19
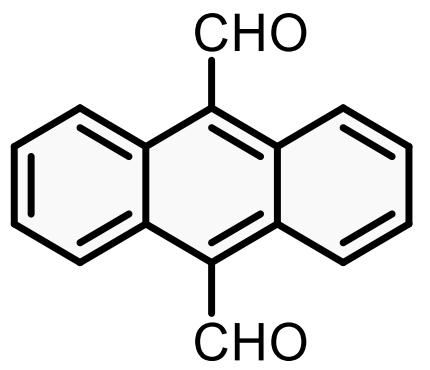	2.58	1.26	0.06
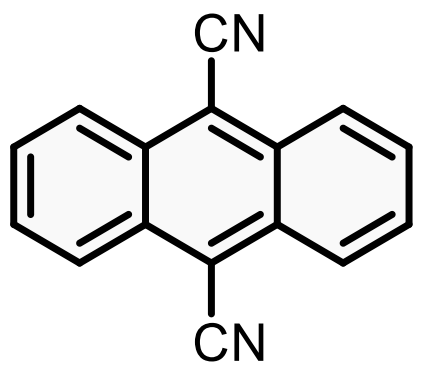	2.84	1.47	-0.10
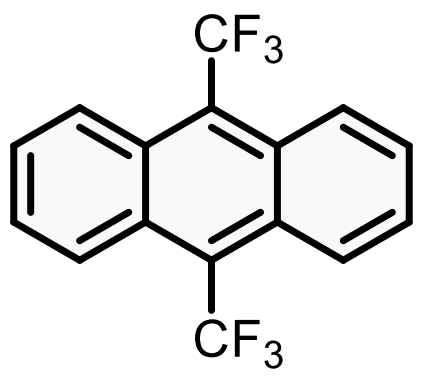	3.03	1.54	-0.05

8. References.

[1] M. J. Frisch, G. W. Trucks, H. B. Schlegel, G. E. Scuseria, M. A. Robb, J. R. Cheeseman, G. Scalmani, V. Barone, G. A. Petersson, H. Nakatsuji, X. Li, M. Caricato, A. V. Marenich, J. Bloino, B. G. Janesko, R. Gomperts, B. Mennucci, H. P. Hratchian, J. V. Ortiz, A. F. Izmaylov, J. L. Sonnenberg, D. Williams-Young, F. Ding, F. Lipparini, F. Egidi, J. Goings, B. Peng, A. Petrone, T. Henderson, D. Ranasinghe, V. G. Zakrzewski, J. Gao, N. Rega, G. Zheng, W. Liang, M. Hada, M. Ehara, K. Toyota, R. Fukuda, J. Hasegawa, M. Ishida, T. Nakajima, Y. Honda, O. Kitao, H. Nakai, T. Vreven, K. Throssell, J. A. Montgomery, Jr., J. E. Peralta, F. Ogliaro, M. J. Bearpark, J. J. Heyd, E. N. Brothers, K. N. Kudin, V. N. Staroverov, T. A. Keith, R. Kobayashi, J. Normand, K. Raghavachari, A. P. Rendell, J. C. Burant, S. S. Iyengar, J. Tomasi, M. Cossi, J. M. Millam, M. Klene, C. Adamo, R. Cammi, J. W. Ochterski, R. L. Martin, K. Morokuma, O. Farkas, J. B. Foresman, D. J. Fox, Gaussian16. Revission C.02., **2019**.

[2] a) A. D. Becke, *J. Chem. Phys.* **1993**, *98*, 5648, b) P.J. Stephens, F.J. Devlin, C.F. Chabalowski, M.J. Frisch, *J.Phys.Chem. 1994*, *98*, 11623.

[3] G. A. Petersson, M. A. Al-Laham, *J. Chem. Phys.* **1991**, *94*, 6081.

[4] a) A. M. Armon, A. Bedi, V. Borin, I. Schapiro, O. Gidron, *Eur. J. Org. Chem.* **2021**, *39*, 5424–5429, b) A. K. Ismael, A. Al-Jobory, *R. Soc. Open Sci.* **2024**, *11*, 23155

[5] C. Hansch, A. Leo, R. W. Taft, *Chem. Rev.* **1991**, *91*, 165–195.

[6] https://docs.pytorch.org/docs/stable/generated/torch.nn.GRU.html

[7] https://docs.pytorch.org/docs/stable/generated/torch.nn.Linear.html

[8] https://docs.pytorch.org/docs/stable/generated/torch.optim.Adam.html

[9] https://deap.readthedocs.io/en/master/

[10] https://deap.readthedocs.io/en/master/examples/pso_basic.html

[11] J. Born, M. Manica, *Nat. Mach. Intell.* **2023**, *5*, 432–444

[12] P. Pracht, F. Bohle, S. Grimme, *Phys. Chem. Chem. Phys*., **2020**, *22*, 7169-7192
